# Safety and immunogenicity of an inactivated whole cell tuberculosis vaccine booster in adults primed with BCG: A randomized, controlled trial of DAR-901

**DOI:** 10.1371/journal.pone.0175215

**Published:** 2017-05-12

**Authors:** C. Fordham von Reyn, Timothy Lahey, Robert D. Arbeit, Bernard Landry, Leway Kailani, Lisa V. Adams, Brenda C. Haynes, Todd Mackenzie, Wendy Wieland-Alter, Ruth I. Connor, Sue Tvaroha, David A. Hokey, Ann M. Ginsberg, Richard Waddell

**Affiliations:** 1 Geisel School of Medicine at Dartmouth, Hanover, New Hampshire, United States of America; 2 Tufts University School of Medicine, Boston, Massachusetts, United States of America; 3 Aeras, Rockville, Maryland, United States of America; Public Health England, UNITED KINGDOM

## Abstract

**Background:**

Development of a tuberculosis vaccine to boost BCG is a major international health priority. SRL172, an inactivated whole cell booster derived from a non-tuberculous mycobacterium, is the only new vaccine against tuberculosis to have demonstrated efficacy in a Phase 3 trial. In the present study we sought to determine if a three-dose series of DAR-901 manufactured from the SRL172 master cell bank by a new, scalable method was safe and immunogenic.

**Methods:**

We performed a single site, randomized, double-blind, controlled, Phase 1 dose escalation trial of DAR-901 at Dartmouth-Hitchcock Medical Center in the United States. Healthy adult subjects age 18–65 with prior BCG immunization and a negative interferon-gamma release assay (IGRA) were enrolled in cohorts of 16 subjects and randomized to three injections of DAR-901 (n = 10 per cohort), or saline placebo (n = 3 per cohort), or two injections of saline followed by an injection of BCG (n = 3 per cohort; 1–8 x 10^6^ CFU). Three successive cohorts were enrolled representing DAR-901 at 0.1, 0.3, and 1 mg per dose. Randomization was performed centrally and treatments were masked from staff and volunteers. Subsequent open label cohorts of HIV-negative/IGRA-positive subjects (n = 5) and HIV-positive subjects (n = 6) received three doses of 1 mg DAR-901. All subjects received three immunizations at 0, 2 and 4 months administered as 0.1 mL injections over the deltoid muscle alternating between right and left arms. The primary outcomes were safety and immunogenicity. Subjects were followed for 6 months after dose 3 for safety and had phlebotomy performed for safety studies and immune assays before and after each injection. Immune assays using peripheral blood mononuclear cells included cell-mediated IFN-γ responses to DAR-901 lysate and to *Mycobacterium tuberculosis* (MTB) lysate; serum antibody to *M*. *tuberculosis* lipoarabinomannan was assayed by ELISA.

**Results:**

DAR-901 had an acceptable safety profile and was well-tolerated at all dose levels in all treated subjects. No serious adverse events were reported. Median (range) 7-day erythema and induration at the injection site for 1 mg DAR-901 were 10 (4–20) mm and 10 (4–16) mm, respectively, and for BCG, 30 (10–107) mm and 38 (15–55) mm, respectively. Three mild AEs, all headaches, were considered possibly related to DAR-901. No laboratory or vital signs abnormalities were related to immunization. Compared to pre-vaccination responses, three 1 mg doses of DAR-901 induced statistically significant increases in IFN-γ response to DAR-901 lysate and MTB lysate, and in antibody responses to *M*. *tuberculosis* lipoarabinomannan. Ten subjects who received 1 mg DAR-901 remained IFN-γ release assay (IGRA) negative after three doses of vaccine.

**Conclusions:**

A three-injection series of DAR-901 was well-tolerated, had an acceptable safety profile, and induced cellular and humoral immune responses to mycobacterial antigens. DAR-901 is advancing to efficacy trials.

**Trial registration:**

ClinicalTrials.gov NCT02063555

## Introduction

Elimination of tuberculosis by 2035 is a major global health priority. This goal cannot be achieved with existing approaches to treatment and prevention [1]. Among newer prevention strategies in development, an improved vaccine strategy against tuberculosis is one of the most promising. Both improved priming vaccines and new booster vaccines are in development; however, modelling indicates that an adolescent and adult booster would have a greater impact on the epidemic over the initial several decades [2–4].

Development of new vaccines against tuberculosis and selection for advancement to human trials has been based largely on molecular discovery and animal challenge models. Candidates include attenuated live vaccines, subunit vaccines and inactivated vaccines [5]. Since no existing animal challenge model predicts vaccine protection in humans, we chose a vaccine candidate based on available clinical observations. Epidemiologic studies indicate that prior infection with either non-tuberculous mycobacteria or *Mycobacterium tuberculosis* itself provides protection against subsequent exposure to tuberculosis [6–8]. Prior tuberculosis vaccine trials have shown that whole cell live bacille Calmette-Guerin (BCG), live *M*. *microti*, and inactivated *M*. *bovis* have each demonstrated efficacy in preventing tuberculosis in humans [9–11].

We therefore hypothesized that human genetic and pathogen antigenic diversity required whole-cell, polyantigenic challenge for protection against tuberculosis [12]. The Dartmouth group began studies with SRL172, an inactivated whole cell BCG booster derived from a heat-inactivated non-tuberculous mycobacterium deposited at the National Collection of Type Cultures (NCTC, London, UK) under accession number 11659. Although originally identified at *Mycobacterium vaccae* by phenotypic methods 16S rRNA gene sequencing of the SRL172 seed strain demonstrates >99.6% homology to the reference 16S rRNA sequence for *Mycobacterium obuense*.

After demonstrating the safety and immunogenicity of a multiple-injection series of SRL172 in Phase 1 and 2 trials we conducted a seven-year, 2,013-subject randomized, controlled Phase 3 trial in Tanzania showing that a five-injection series had acceptable safety, was immunogenic, and reduced culture-confirmed tuberculosis by 39% in HIV-infected persons [13–16]. The agar-based manufacturing method for SRL-172 was not scalable.

We have now developed a new, scalable broth-based manufacturing method using the original master cell bank for SRL172 to produce the inactivated DAR-901 booster vaccine. In the present randomized, controlled dose-escalation trial we sought to evaluate the safety, tolerability and immunogenicity of a three-injection series of up to 1 mg DAR-901 to inform the decision to advance DAR-901 to efficacy trials.

## Materials and methods

### Study design and participants

**Fig 1:**
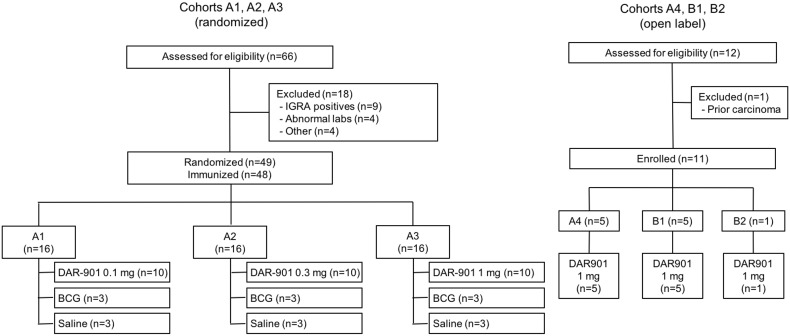
Consort diagram for cohorts A1, A2, A3 A4, B1 and B2.

We recruited and screened healthy adult subjects aged 18–65 with a history of prior BCG immunization for the trial. We obtained written informed consent from all subjects. Subjects for the randomized double-blind, dose-escalation cohorts (A1, A2 and A3 in Table 1) were required to have a negative HIV ELISA (Vitros Anti-HIV 1 + 2 test, Ortho Clinical Diagnostics, Rochester, NY), a negative interferon gamma release assay (IGRA, T SPOT.*TB*, Oxford Immunotec), a normal physical examination, acceptable safety laboratory results, be negative for hepatitis B surface antigen and hepatitis C antibody, and be free of chronic illness. The target sample size for cohorts A1-3 was 48 (Fig 1). We recruited IGRA-positive and HIV-positive subjects for the open-label cohorts (A4, B1 and B2) (Table 1). The target sample size for cohorts A4, B1 and B2 was 14–22 (Fig 1). HIV-infected subjects were recruited from the HIV Care Program of the Dartmouth-Hitchcock Medical Center (DHMC). These subjects were required to have at least one previous positive HIV viral load, to have been on stable antiretroviral therapy (ART) for the past 3 months, not to have had a CD4+T- cell count<200/mm^3^ within the past year and not to have Grade 2 or higher abnormalities on laboratory safety tests. All female subjects were required to have a negative urine pregnancy test before each injection of vaccine or placebo (Clarity hCG, Clarity Diagnostics, Boca Raton, FL, USA).

**Table 1:** Study cohorts by HIV status, IGRA status, and DAR-901 dose level.

Cohort	HIV status	IGRA status	DAR-901		BCG (N)	Placebo (N)	Total
			Dose	(N)			
A1	Neg	Neg	0.1 mg	10	3	3	16
A2	Neg	Neg	0.3 mg	10	3	3	16
A3	Neg	Neg	1 mg	10	3	3	16
A4	Neg	Pos	1 mg	5	-	-	5
B1	Pos	Neg	1 mg	5	–	-	5
B2	Pos	Pos	1 mg	1	–	-	1
**Total**				**41**	**9**	**9**	**59**

Neg, Negative; Pos, Positive

Cohorts A1-A3, randomized, double-blind; Cohorts A4, B1 and B2 open label

### Vaccination materials

DAR-901 was produced by Aeras (Rockville, MD, USA) in compliance with Good Manufacturing Practices and provided in 2 mL individual dose vials containing a 0.3 mL suspension of heat-inactivated organisms. Saline placebo was obtained as Sterile Saline for Injection, USP; BCG was obtained as Tice BCG, 1–8 x 10^6^ CFU (Organon Teknika, Durham, North Carolina, USA).

### Study procedures including randomization and masking

The first subject in cohorts A1, A2, and A3 received open-label DAR-901. When acceptable safety was confirmed 3 days after immunization, the remaining subjects in each cohort were randomized 3:1:1 to receive three injections of DAR-901 (7 x 10^6^ CFU/ for 1 mg; 2 x 10^6^ CFU for 0.3 mg; and 0.7 x 10^6^ CFU for 0.1 mg), three of saline placebo, or two of saline followed by BCG (1–8 x 10^6^ CFU). Computer-generated randomization was performed centrally and provided to the study pharmacist who filled a tuberculin syringe to 0.1 mL with the agent specified. The pre-filled syringe was given to an injection nurse who administered the intradermal injection but was not involved in any subsequent evaluations. Separate study nurses and study physicians conducted all subject assessments to ensure that blinding to treatment allocation was maintained throughout the trial. A three-person expert Dose Review Committee approved escalation to the next doses level after review of 7-day safety data on all subjects in the previous cohort.

**Fig 2:**
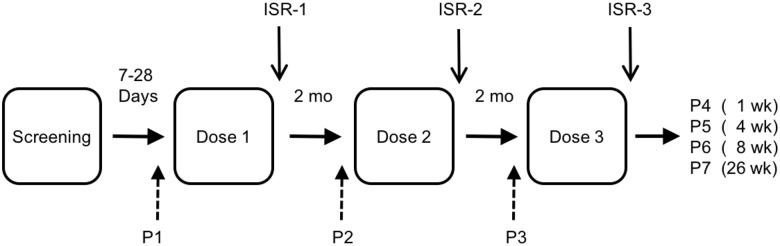
Study design.

We administered the first dose of vaccine or placebo within 28 days of screening; subsequent doses were administered at 2 and 4 months (Fig 2). All doses were administered at Dartmouth-Hitchcock Medical Center as intradermal injections over the deltoid muscle, alternating between the left and right arms.

### Safety assessments

We repeated physical examination, vital signs and safety laboratory tests before each dose of vaccine and at 28 days and 6 months after dose 3 (End of Study, EOS, 180 days). Vital signs were repeated on all subjects 30–60 minutes after each dose. Subjects were seen 7 days after each dose of vaccine for physical examination, vital signs and examination of the injection site (Fig 2). Injection site reactions were measured as mm in the transverse diameter. In addition, after each dose of vaccine, subjects were contacted at days 3, 5 and 7 after injection and twice weekly for three additional weeks to report daily self-measured temperature, self-measured vaccine site reactions, and were questioned regarding local and systemic adverse events (AEs).

Safety laboratory studies included complete blood count, serum creatinine, glucose, liver function tests, CPK and urinalysis. HIV-positive subjects in cohorts B1 and B2 had HIV viral load determinations at the same intervals (COBAS^®^ TaqMan^®^ HIV-1 Test, Roche Molecular Diagnostics, Basel, Switzerland, detection limit = 20 HIV RNA copies/mL).

We graded injection site reactions, abnormal laboratory values, and all other adverse events based on guidelines for vaccine trials from the United States Food and Drug Administration [17], which are appreciably stricter than the scales typically used in clinical trials of therapeutics.

### Immune assays

We collected blood for immune assays from subjects in cohorts A1, A2, and A3 at baseline (pre-dose 1), pre-dose 2, pre-dose 3, and at 7, 28, 56 and 180 days after dose 3 (Fig 2). For subjects in cohorts A4, B1 and B2 we collected samples at baseline and 56 days post dose 3. We repeated IGRA assays on subjects in Cohort A3 at 2–6 months after dose 3.

We isolated peripheral blood mononuclear cells (PBMC) by Ficoll-Hypaque density gradient separation. Cells were cryopreserved and, after thawing, cultured in a 96-well tissue culture plates for 18–24 hrs in RPMI supplemented with 10% fetal bovine serum (Mediatech) with equal volumes of medium (negative control) or assay antigens. Triplicate samples were pooled and the concentration of IFN-γ assessed by ELISA (Affymetrix, Santa Clara, CA, USA). Antigens included phytohemaglutinin at 5 mcg/ml (positive control; Sigma), *M*. *tuberculosis* whole cell lysate (WCL) at 2 mcg/ml, or DAR-901 lysate at 1 mcg/ml. Plates for IFN-γ were read on a micro plate reader (BioTek Synergy2, Winooski, VT, USA). Standard control curves used recombinant protein human IFN-γ. Anti-LAM antibody concentrations were assessed in singlet serum samples by a proprietary ELISA [14] and read on a micro plate reader (BioTek Synergy2, Winooski, VT, USA). A separate report will detail results of multiparameter intracellular cytokine stimulation assays performed using separate aliquots of PBMCs.

### Outcomes and statistical analysis

The objective of the trial was to determine a dose of DAR-901 for further clinical trials that had an acceptable safety profile and induced both humoral and cellular immune responses to mycobacterial antigens. The sample size for the present study was based on prior experience with Phase 1 vaccine studies and represents the number of participants needed to permit preliminary evaluation of safety and tolerability and support advancement of a vaccine development program.

We performed the safety analysis on all enrolled subjects who received at least one injection of study treatment. Solicited and un-solicited AEs were classified by the Medical Dictionary for Regulatory Activities (MeDRA)- preferred term and compared between treatment groups. Injection site reactions were deemed related to immunization. The Principal Investigator assessed other adverse events for their relationship to immunization. Safety laboratory studies with values outside pre-defined reference ranges were assessed for clinical significance.

For immune assays, we compared pre- and post-vaccination responses using Wilcoxon signed rank tests and, secondarily, vaccine vs placebo and vaccine vs BCG responses using the Mann-Whitney U test. GraphPad Prism software was used for the statistical analyses.

For all analyses, we assigned a P-value <0.05 as the cutoff for statistical significance.

The trial is registered with ClinicalTrials.gov as NCT02063555. The study was approved by the Dartmouth Committee for the Protection of Human Subjects.

### Role of the funding source

The Dartmouth and Aeras study teams were involved in the study design, interpretation of data and writing the report. The corresponding author had access to all data and had final responsibility for data analysis and writing the study report.

## Results

### Participants and study treatments

We screened 78 individuals to enroll 59 subjects. For IGRA-negative cohorts A1-A3 a total of 66 subjects were screened to obtain 49 eligible subjects who were randomized; one subject withdrew prior to immunization and was replaced, leaving 48 subjects in the A cohorts. The 18 subjects ineligible for cohorts A1-A3 included 9 who were IGRA-positive, 4 with abnormal laboratory results, 1 who was unable to return for follow-up, 1 without a BCG scar, 1 on systemic steroids, and 1 who was unable to tolerate phlebotomy (41). For IGRA-positive cohort A4 we screened a total of 5 subjects and all were eligible. For HIV-positive cohorts B1 and B2 we screened a total of 7 subjects to obtain 6 eligible subjects. One was ineligible due to a prior diagnosis of carcinoma (Fig 1). Characteristics of study subjects are shown in Table 2.

**Table 2:** Characteristics of study subjects.

Characteristic	Cohorts A1-A4 (N = 53)	Cohorts B1- B2 (N = 6)
HIV status	Negative	Positive
Age, years, median (range)	27 (18–61)	47 (37–61)
Female sex, number (%)	30 (57%)	3 (50%)
Race, number (%)		
Black or African-American	8 (15%)	3 (50%)
Asian	20 (38%)	0 (0%)
White	25 (47%)	3 (50%)
Weight, kg, median (range)	70 (44–112)	71 (46–97)
BMI, median (range)	24 (17–36)	28 (21–31)
CD4+ T cell count, cells/mm3, median (range)	NA	656 (330–1092)

NA, not applicable.

Cohorts A1-A3, randomized, double-blind; Cohorts A4, B1 and B2 open label

In cohorts A1-A3 a total of 47 of 48 subjects received all three study injections. Study investigators withdrew one subject (DAR-901, 1mg) after dose 2 due to hematuria that was subsequently attributed to a new diagnosis of schistosomiasis. All 48 subjects in cohorts A1-A3 completed follow-up through 6 months after immunization. In cohorts A4, B1 and B2 all 11 subjects received all three study injections. One subject withdrew 28 days after dose 3 citing inadequate time to complete study visits. The remaining 10 of 11 subjects completed follow-up through 6 months of immunization. The first subject was enrolled on April 28, 2014 and the last study visit conducted on February 19, 2016.

### Injection site reactions

Erythema and induration at the injection site were common in DAR-901 recipients. All reactions were mild and none met FDA criteria for Grade 1 or higher. Tables 3 and 4 summarize erythema and induration at the vaccine site as measured 7 days after each dose of vaccine in 53 HIV-negative subjects. Reactions were similar for the IGRA-negative (n = 10) and IGRA-positive (n = 5) subjects who received 1mg DAR-901, with median erythema of 10 mm and 12 mm, respectively, and median induration of 8 mm and 12 mm, respectively. Median erythema at 7 days typically increased slightly with increasing DAR-901 dose level and dose number. There was no discernible pattern in median induration by dose level or dose number.

**Table 3:** Erythema (mm) measured 7 days after immunization in 53 HIV-negative subjects.

Treatment	DAR-901	DAR-901	DAR-901	BCG	Placebo
DAR-901 Dose	0.1 mg	0.3 mg	1 mg	–	–
Cohort	A1	A2	A3, A4	A1-A3	A1-A3
Number of subjects	10	10	15	9	9
**Erythema**					
**Overall**					
**N with reaction**	10	9	15	9	0
**Mean (SD)**	7.9 (2.8)	8.3 (1.3)	11.7 (4.7)	39.3 (28.3)	
**Median**	8.0	8.0	10.0	30.0	
**Min, Max**	2, 13	3, 10	3, 20	6, 107	
**Dose 1**					
**N with reaction**	9	9	15	1	0
**Mean (SD)**	5.6 (1.9)	6.8 (1.6)	10.0 (3.8)	6.0	
**Median**	6.0	6.0	10.0	6.0	
**Min, Max**	3, 8	5, 10	4, 20	6, 6	
**Dose 2**					
**N with reaction**	10	8	13	0	0
**Mean (SD)**	6.1 (2.9)	6.4 (2.5)	10.5 (4.8)		
**Median**	6.0	7.0	10.0		
**Min, Max**	2, 10	3, 10	3, 20		
**Dose 3**					
**N with reaction**	10	9	13	9	0
**Mean (SD)**	6.7 (3.5)	7.7 (1.1)	9.8 (4.9)	39.3 (28.3)	
**Median**	7.0	8.0	8.0	30.0	
**Min, Max**	3, 13	6, 10	4, 20	10, 107	

Cohorts A1-A3, randomized, double-blind; Cohorts A4, B1 and B2 open label

**Table 4:** Induration (mm) measured 7 days after immunization in 53 HIV-negative subjects.

Treatment	DAR-901	DAR-901	DAR-901	BCG	Placebo
DAR-901 Dose	0.1 mg	0.3 mg	1 mg	–	–
Cohort	A1	A2	A3, A4	A1-A3	A1-A3
Number of subjects	10	10	15	9	9
**Induration**					
**Overall**					
**N with reaction**	10	10	15	9	1
**Mean (SD)**	6.9 (3.4)	7.0 (2.9)	9.9 (4.0)	34.7 (14.6)	2.0
**Median**	7.5	7.0	10.0	38.0	2.0
**Min, Max**	1, 12	2, 11	1, 16	1, 55	2, 2
**Dose 1**					
**N with reaction**	8	9	15	1	1
**Mean (SD)**	7.4 (2.5)	5.2 (2.5)	7.7 (4.4)	1.0	2.0
**Median**	7.0	4.0	7.0	1.0	2.0
**Min, Max**	4, 12	2, 10	1, 16	1, 1	2, 2
**Dose 2**					
**N with reaction**	10	10	14	0	0
**Mean (SD)**	3.9 (1.7)	6.3 (3.1)	6.5 (3.2)		
**Median**	4.0	6.5	6.0		
**Min, Max**	2, 8	2, 11	2, 14		
**Dose 3**					
**N with reaction**	9	10	13	9	0
**Mean (SD)**	5.1 (3.6)	3.8 (1.6)	7.1 (3.6)	34.7 (14.6)	
**Median**	4.0	3.0	6.0	38.0	
**Min, Max**	1, 10	2, 6	4, 16	15, 55	

Cohorts A1-A3, randomized, double-blind; Cohorts A4, B1 and B2 open label

Among 10 HIV-negative subjects in each of cohorts A1 and A2 receiving 0.1 or 0.3 mg DAR-901 we noted no cutaneous breakdown. Among 10 HIV- negative/IGRA-negative subjects in cohort A3 receiving 1 mg DAR-901 cutaneous reactions included: 1 vesicle (4 mm, dose 1), 1 crust (2 mm, dose 1) and 8 mild desquamation. Among 5 HIV-negative/IGRA-positive subjects in cohort A4 receiving 1 mg DAR-901 cutaneous reactions included: 1 pustule (1 mm, dose 3) and 2 crusts (3 and 5 mm, dose 3)(Table 5). All local reactions healed spontaneously; three of 10 subjects at the 1 mg dose in A3 had visible erythema or scar at the dose 3 site at the end-of-study (EOS) visit (range 5–6 mm).

**Table 5:** Injection site reactions and solicited symptoms in 53 HIV-negative subjects 7 days after immunization.

Treatment	DAR-901	DAR-901	DAR-901	BCG	Placebo
DAR-901 Dose	0.1 mg	0.3 mg	1 mg	–	–
Cohort	A1	A2	A3, A4	A1-A3	A1-A3
Number of subjects	10	10	15	9	9
**Number with reaction (%)**					
Erythema	10 (100%)	9 (90.0%)	15 (100%)	9 (100%)	0
Desquamation	0	0	8 (53%)	4 (44%)	0
Induration	10 (100%)	10 (100.0%)	15 (100%)	9 (100%)	1 (11%)
Vesicle/Blister	0	0	1 (7%)	1 (11%)	0
Pustule	0	0	1 (7%)	0	0
Erosion	0	0	2 (13%)	1 (11%)	0
Crust	0	0	3 (20%)	1 (11%)	0
Axillary Adenitis	0	0	0	0	0
**Number with symptom (%)**					
Feverish	0	0	0	0	0
Malaise	1 (10%)	2 (20%)	0	0	0
Muscle Aches	2 (20%)	0	1 (7%)	0	0
Tenderness	0	1 (10%)	1 (7%)	4 (44%)	0
Sore Arm	0	1 (10%)	0	1 (11%)	0
Itching at Site	0	2 (20%)	3 (20%)	4 (44%)	0
Number with ≥1 symptom	2 (20%)	4 (40%)	5 (33%)	6 (67%)	0

Cohorts A1-A3, randomized, double-blind; Cohorts A4, B1 and B2 open label

For 6 HIV-positive subjects in cohorts B1 and B2 median erythema at Day 7 was 12 mm (range 7–12) and median induration 8 mm (range 7–12). No subjects had pustules, crusts or desquamation at the injection site.

For 9 HIV-negative subjects who received BCG for dose 3 median injection site reactions at 7 days were 30 mm for erythema and 38 mm for induration. By the 28 day visit most subjects had skin breakdown with drainage or ulceration (data not shown).

Solicited symptoms at 7 days for 53 HIV-negative subjects are shown in Table 5. Among the 6 HIV-positive subjects, one reported tenderness, and one pruritus at the vaccine site.

### Adverse events and laboratory results

There were no serious adverse events (AEs). AEs excluding injection site reactions are shown in Table 6. There was no difference in the distribution of AEs between DAR-901 recipients and saline placebo or BCG cohorts. Within the DAR-901 cohorts there were no patterns of reaction within organ systems to suggest an effect of the vaccine.

**Table 6:** Adverse events excluding injection site reactions.

HIV Status	Neg	Neg	Neg	Neg	Neg	Neg	Pos
IGRA Status	Neg	Neg	Neg	Neg	Neg	Pos	–
Treatment	DAR-901	DAR-901	DAR-901	BCG	Placebo	DAR-901	DAR-901
DAR-901 Dose	0.1 mg	0.3 mg	1 mg	–	–	1 mg	1 mg
Cohort	A1	A2	A3	A1-A3	A1-A3	A4	B1,B2
Number of subjects	10	10	10	9	9	5	6
	Number (%) of Subjects						
**SAE**	0	0	0	0	0	0	0
**≥ 1 AE**	6 (60%)	10 (100%)	7 (70%)	8 (89%)	7 (78%)	4 (80%)	5 (83%)
**Related AE**^1^	0	0	2 (20%)	2 (22%)	0	1 (20%)	0

Cohorts A1-A3, randomized, double-blind; Cohorts A4, B1 and B2 open label

AE, adverse event; SAE, serious adverse event

^**1**^ Among the five related AEs, four were headache (mild) and one (a BCG subject) was muscle ache (mild).

One HIV-positive subject with treated hypertension had a Grade 3 increase in blood pressure one hour after phlebotomy and dose 2 injection (from 140/90 to 160/100) on a day when she reported she had failed to take her anti-hypertensive medications. No other Grade 3 changes in vital signs were noted.

Mild CPK elevations were common at baseline in physically active subjects (data not shown). Treatment-emergent CPK elevations Grade 1 or higher were noted in 25 of 59 (42%) subjects during the study including in 14 of 41 (34%) DAR-901 recipients, 6 of 9 (67%) BCG recipients and 4 of 9 (44%) placebo recipients. Grade 3 CPK elevations (3.1 to 10x ULN) were noted in 6 subjects; one Grade 4 reaction occurred in a placebo recipient with a clinical diagnosis of influenza. Treatment was not required for any CPK abnormality. There was one Grade 3 episode of hyperglycemia (random, >200 mg/dL) 2 months after dose 2 in a placebo recipient with a medical history of mild glucose intolerance; dietary treatment was continued. One HIV-positive subject who received 1.0 mg DAR-901 and had a prior history of fluctuating hemoglobin levels had a Grade 3 hemoglobin decrease pre-dose 3 which resolved spontaneously to pre-study levels 28 days later. No other Grade 3 laboratory abnormalities were noted.

HIV viral loads by PCR were <20 copies/mL on all 6 HIV positive subjects at screening. Subjects had 4 additional viral loads determined during the study (pre-1, pre-2, pre-3 and 28 days after dose 3). The value for one subject was 70 copies/mL on Day 1 prior to dose 1, but <20 copies/mL on 3 subsequent determinations. One subject (772) had a viral load of 430 copies/mL pre-dose 3 which was suspected by her primary caregiver to be related to a lapse in taking her anti-retroviral therapy. After counseling her repeat viral load 28 days after dose 3 was <20 copies/mL. All other viral loads were <20 copies/mL on all subjects at all additional time points. Collectively these data confirm that a three dose series of 1 mg DAR-901 does not have an adverse effect on HIV viral load.

### Immune assays

**Fig 3:**
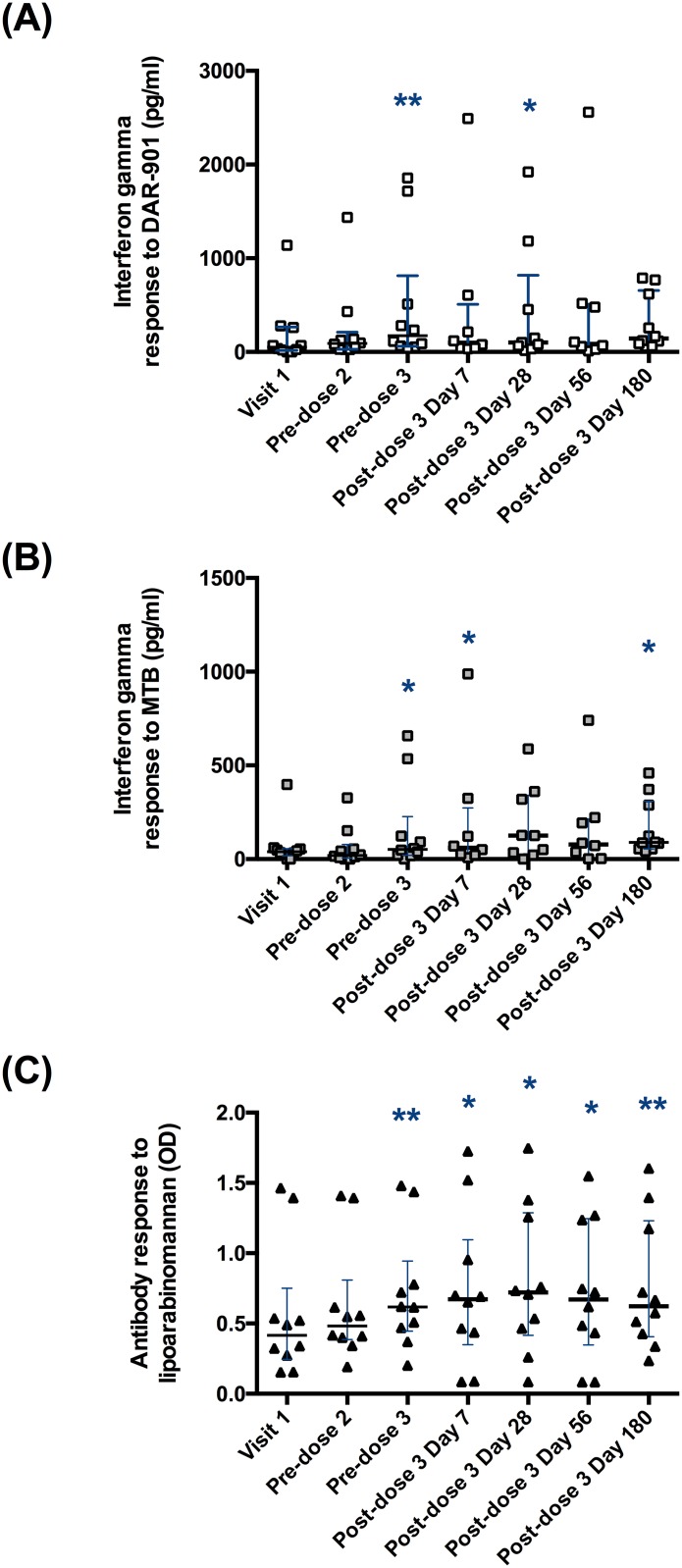
Immunogenicity of DAR-901 among 10 subjects who received three injections of 1 mg DAR-901 (Cohort A3). Samples for Visit 1, pre-dose 2, and pre-dose 3 were collected 2 months apart. (A) Immunization with DAR-901 at the 1 mg dose elicited greater interferon gamma responses (IFN-γ) to DAR-901 lysate at pre-dose 3 and post-dose 3 day 28 compared to pre-vaccination levels. (B) IFN-γ responses to *Mycobacterium tuberculosis* (MTB) whole cell lysate were significantly greater, compared to pre-vaccination levels pre-dose 3, post-dose 3 day 7, and 180 (EOS). (C) Antibody responses to MTB lipoarabinomannan (LAM) were significantly greater than pre-vaccination responses at pre-dose 3, and post-dose 3 day 7, day 28, 56, and 180 (EOS). Graphs depict individual subject values along with error bars indicating median and interquartile range; * indicates P<0.05; ** indicates P<0.01.

Fig 3 depicts immune responses to DAR-901 lysate after immunization with DAR-901 in 10 subjects who received 1 mg DAR-901. For each dose samples were drawn pre-dose and then at Days 7, 28, and 56 post-dose 3, and at 6 months post-dose 3 at the End of Study (EOS). Compared to baseline Visit 1 responses, median IFN-γ responses to DAR-901 lysate among vaccine recipients were significantly greater pre-dose 3 (52 vs. 173 pg/ml, P = 0.005), and post dose-3 at day 28 (52 vs. 102 pg/ml, P = 0.011) with a trend toward significance at day 56 (52 vs. 87 pg/ml, P = 0.050) (Fig 3A). Compared to pre-dose 1 responses IFN-γ responses to MTB whole cell lysate in DAR-901 recipients were significantly greater pre-dose 3 (40 vs 52 pg/ml, P = 0.0469), day 7 post dose 3 (40 vs 60 pg/ml, P = 0.0499), and at 6 month EOS (40 vs. 89 pg/ml, p = 0.0469) (Fig 3B). Compared to pre-dose 1 responses anti-LAM antibody responses to DAR-901 lysate among vaccine recipients were significantly greater at pre-dose 3 (optical density [OD] 0.416 vs. 0.618, P = 0.005), and post-dose 3 at day 7 (OD 0.416 vs. 0.673, P = 0.013), day 28 (OD 0.416 vs. 0.721, P = 0.013), day 56 (OD 0.416 vs 0.671, P = 0.037), and at 6 month EOS (OD 0.416 vs. 0.622, P = 0.005) (Fig 3C). See also S1 Fig. There was no significant increase in IFN-γ response to DAR-901 lysate from baseline to any subsequent timepoint in subjects who received DAR-901 at the 0.1 mg (Cohort A1) or 0.3 mg (Cohort A2) doses.

There were no statistically significant differences at any study visit in absolute or ordinal IFN-γ responses to DAR-901 lysate or anti-LAM responses between vaccine and placebo subjects (n = 3 per cohort), nor between HIV-negative and HIV-positive study subjects or between IGRA-negative and IGRA- positive subjects.

There were no differences in IFN-γ responses to DAR-901 lysate between the 10 subjects in the 1 mg A3 cohort and the 9 subjects in the BCG cohort. Median IFN-γ responses to MTB lysate were significantly greater among 9 BCG subjects compared to 10 subjects in the 1 mg DAR-901 A3 cohort pre-dose 1 (284 vs. 40 pg/ml, p = 0.028), pre-dose 2 (304 vs 19 pg/ml, p = 0.003), and post-dose 3 at day 28 (2,624 vs. 125 pg/ml, p = 0.005), day 56 (922 vs. 79 pg/ml, p = 0.002) and 6 month EOS (862 vs. 89 pg/ml, p = 0.004). See also S2 Fig. There were no differences in the levels of anti-LAM antibody response at any visit between the 9 BCG subjects or the 10 subjects in the 1 mg A3 DAR-901 cohort.

Repeat IGRA assays 2–6 months after dose 3 on 10 baseline IGRA-negative subjects who received 1 mg DAR-901 were all negative. The subject with schistosomiasis received two doses of DAR-901: Dose 2 was received on January 2, 2015; the initial repeat IGRA on July 10, 2015 was positive at 0.70; repeat on August 14, 2015, was negative at 0.00.

## Discussion

Development of DAR-901 has been based on clinical and epidemiologic studies in humans. Animal models remain an imperfect simulation of the complex natural history of *M*. *tuberculosis* infection and vaccine-induced protection in humans. Nor is there a validated *in vitro* correlate of tuberculosis vaccine efficacy to justify the use of immune assays in selecting optimal vaccine constructs. Epidemiologic skin test studies demonstrate that prior infection with either *M*. *tuberculosis* or non-tuberculous mycobacteria confers protection against disease from subsequent tuberculosis exposure [6–8]. Prior vaccine trials indicate that live BCG, live *M*. *microti*, inactivated *M bovis* and inactivated non-tuberculous mycobacterial reagents all confer protection against tuberculosis in humans [9–11]. Although variations in the efficacy of BCG have been observed, efficacy has been high in all studies where mycobacteria-naïve infants were immunized at birth [18]. In all of these studies immune protection against tuberculosis has only been observed in response to polyantigenic, whole-organism exposure or infection. Of note, such protection is not species-specific within the genus. A whole cell vaccine derived from a non-tuberculous mycobacterium was therefore selected to simulate the whole organism exposures known to confer immune protection against tuberculosis in humans.

We have shown previously that immunization with agar-produced SRL172, an inactivated whole cell non-tuberculous mycobacterial vaccine has an acceptable safety profile and is well tolerated. In a Phase 3 trial in HIV-infected patients in Tanzania SRL172 was both immunogenic and effective in preventing culture-confirmed tuberculosis [13]. That trial was designed with 5 doses before we had immune response data from a Phase 2 trial in Finland which demonstrated significant immune responses after 3 doses [15]. DAR-901 is the scalable, broth-produced formulation made from the SRL172 Master Cell Bank. In the present Phase 1 study we demonstrate that 3 doses of DAR-901 have an acceptable safety profile, are well tolerated as a BCG booster, and produce injection site reactions comparable to those observed with SRL172. In addition DAR-901 induces both cellular and humoral responses to polyantigenic mycobacterial lysates as observed previously with agar-manufactured SRL172 [14]. This is the only inactivated whole cell investigational vaccine against tuberculosis to have shown efficacy in humans.

The subjects in this Phase 1 trial represent a spectrum of subjects who will be candidates for DAR-901 boosting in tuberculosis-endemic countries including HIV-negative, HIV-positive, IGRA-negative and IGRA-positive persons. Extensive safety monitoring, including day-7 visits after each dose, twice weekly phone contact for 28 days after each dose, and a final visit 6 months after the last dose, indicate that DAR-901 has an acceptable safety profile and is well-tolerated. Other than injection site reactions the only adverse event judged possibly related to DAR-901 was mild headache observed in 3 of 21 subjects who received the 1 mg dose. There were no clinically significant treatment-emergent vital signs or laboratory abnormalities related to DAR-901.

Measured reactions at the injection site were typically limited to mild erythema and induration and were not associated with significant discomfort. Superficial desquamation or erosion was noted in a minority of subjects either at 7 days or later over the course of the study. These reactions were comparable to those observed with a 1 mg dose of SRL172 in the Phase 3 study [13]. In contrast BCG was associated with drainage or skin breakdown in most subjects 1–2 months after immunization.

The immune assays in the present study showed that 1 mg DAR-901 induced IFN-γ responses to the vaccine lysate and to *M*. *tuberculosis* lysate compared to baseline. Mean OD values for antibody to LAM increased significantly prior to dose 3, comparable to the responses seen after 5 doses of SRL172 [14]. IFN-γ responses to *M*. *tuberculosis* lysate and antibody responses to LAM remained statistically significant 6 months after immunization. In this Phase 1 study with a small sample size we did not demonstrate statistically significant differences in immune response between the 3 placebo recipients and 10 vaccine recipients in any of the three blinded dose cohorts. In order to avoid type II errors we did not adjust our threshold for statistical significance to correct for multiple comparisons in our analyses of immune responses.

IFN-γ responses to MTB lysate among BCG recipients in this trial were substantially higher than those observed after three doses of DAR-901. However, SRL172 has been shown effective as a booster while two large randomized trials have shown that BCG boosters are not effective in the prevention of tuberculosis [19,20]. Indeed, in the Malawi booster trial there was a trend toward a higher rate of tuberculosis among BCG recipients than control recipients [20]. This raises the interesting question of whether BCG recipients might have had excessive IFN-γ responses, which have been shown to be detrimental in immune control of tuberculosis in in experimental studies [21].

The target product profile for DAR-901 is a booster vaccine for BCG-primed adolescents and adults living in tuberculosis-endemic countries. SRL172 is the only new tuberculosis vaccine shown effective in humans, but the agar-based production method was not suitable for mass distribution. DAR-901 is produced from the same seed strain as SRL172, but manufactured by a scalable, broth-grown procedure. In the present trial we have shown that DAR-901 has an acceptable safety profile, is well-tolerated in a spectrum of BCG-primed adults representing groups who will be candidates for boosting in tuberculosis-endemic countries and induces significant IFN-γ and antibody responses to polyantigenic mycobacterial antigens. Importantly, DAR-901 did not result in IGRA conversion, indicating that it can be studied in a prevention of infection trial. DAR-901 is now entering a fully powered, Phase 2b randomized, controlled, prevention of infection trial among adolescents in Tanzania.

## Supporting information

S1 Fig Immunogenicity of DAR-901 by individual subject in cohort A3. Immunogenicity of DAR-90 among 10 subjects who received three injections of 1 mg DAR-901 (Cohort A3). Samples for Visit 1, pre-dose 2, and pre-dose 3 were collected 2 months apart. (A) Interferon gamma responses (IFN-γ) to DAR-901 lysate. (B) IFN-γ responses to *Mycobacterium tuberculosis* (MTB) whole cell lysate. (C) Antibody responses to MTB lipoarabinomannan (LAM). Graphs depict responses of individual subjects at each study visit.
(TIFF)

S2 Fig Immunogenicity of 1 mg DAR-901 vs BCG. Interferon gamma (IFN-γ) responses to *Mycobacterium tuberculosis* (MTB) whole cell lysate among 10 subjects who received three injections of 1 mg DAR-901 (Cohort A3) compared to 9 subjects who received BCG 1-8x10^6^ organisms in 0.1 mL. Samples for Visit 1, pre-dose 2, and pre-dose 3 were collected 2 months apart and were obtained prior to dose 1, 2 and 3 respectively. Bacille Calmette Guerin (BCG) recipients exhibited greater IFN-γ responses to MTB lysate at multiple timepoints after dose 3. Graphs depict individual data points along with median values for all subjects at that timepoint. Gray-shaded circles represent IFN-γ responses to DAR-901 and black-shaded circles IFN-γ responses to BCG. B and BCG, bacille Calmette Guerin 1-8x10^6^ organisms in 0.1 mL; D or DAR, DAR-901 1 mg dose; S, saline.
(TIFF)

S1 File CONSORT checklist. (DOC)

S2 File DAR-901-MDES protocol v 4.1 March 30, 2015. (PDF)
