# Supplementary figures and images for: Safety and immunogenicity of an inactivated whole cell tuberculosis vaccine booster in adults primed with BCG: A randomized, controlled trial of DAR-901
Source: PLoS One. 2017 May 12;12(5):e0175215. doi: 10.1371/journal.pone.0175215 (PMC5429024; doi:10.1371/journal.pone.0175215)

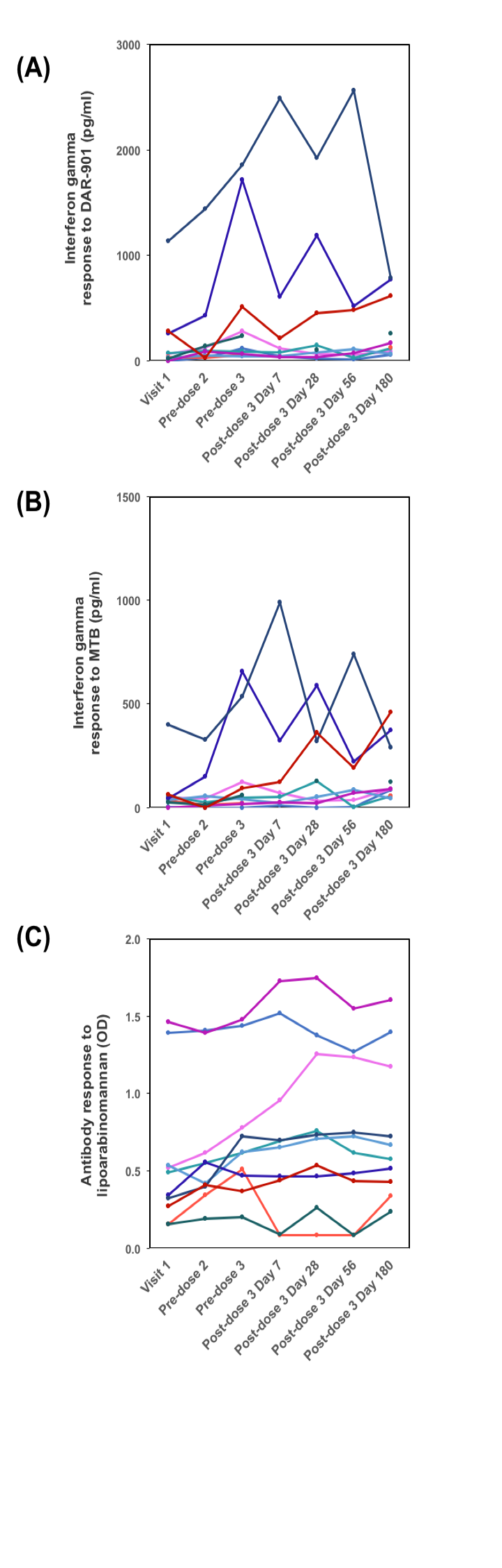

Supplement: S1 Fig — Immunogenicity of DAR-90 among 10 subjects who received three injections of 1 mg DAR-901 (Cohort A3). Samples for Visit 1, pre-dose 2, and pre-dose 3 were collected 2 months apart. (A) Interferon gamma responses (IFN-γ) to DAR-901 lysate. (B) IFN-γ responses to Mycobacterium tuberculosis (MTB) whole cell lysate. (C) Antibody responses to MTB lipoarabinomannan (LAM). Graphs depict responses of individual subjects at each study visit. (TIFF) [file pone.0175215.s001.tiff]

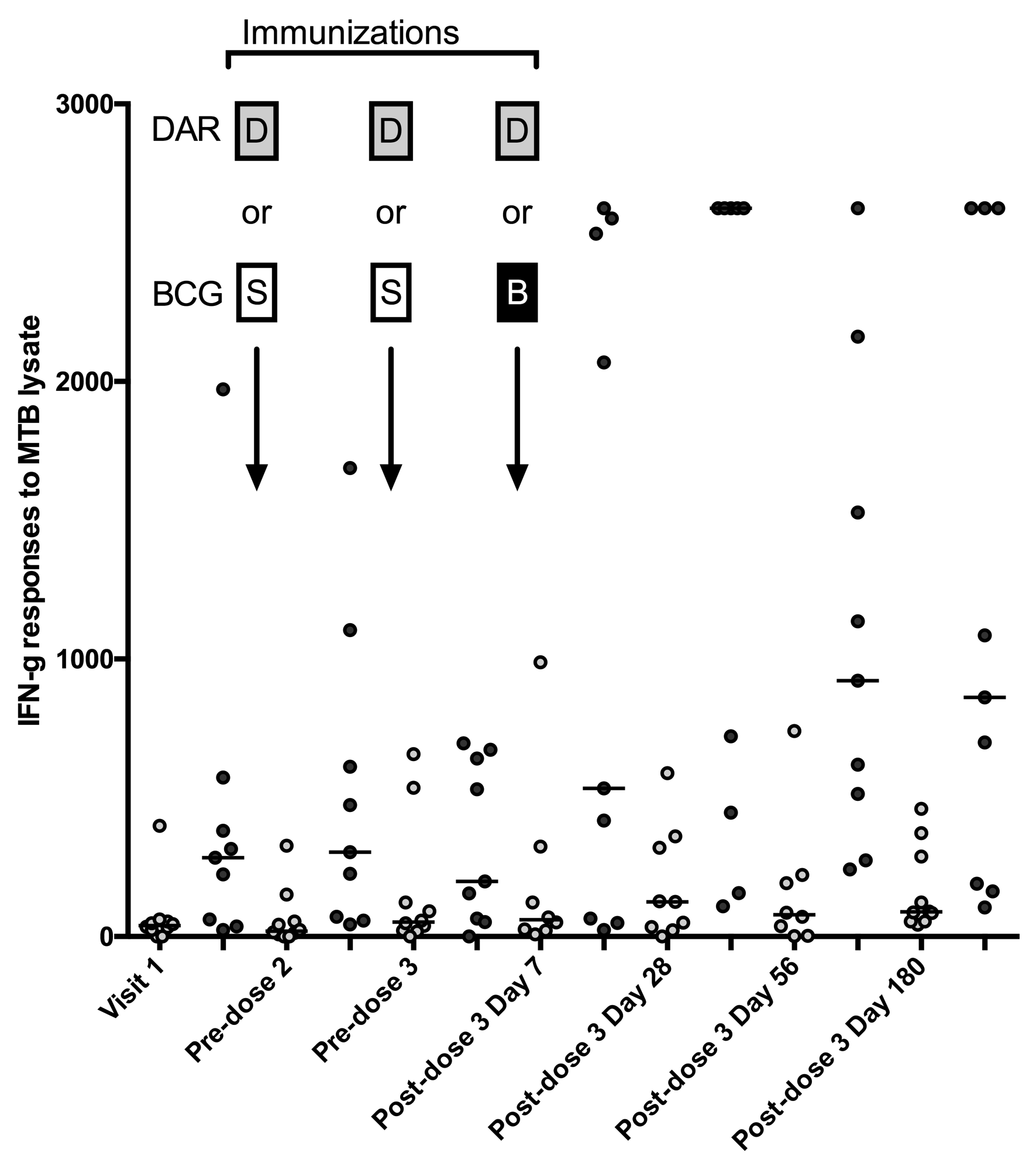

Supplement: S2 Fig — Interferon gamma (IFN-γ) responses to Mycobacterium tuberculosis (MTB) whole cell lysate among 10 subjects who received three injections of 1 mg DAR-901 (Cohort A3) compared to 9 subjects who received BCG 1-8x10^6^ organisms in 0.1 mL. Samples for Visit 1, pre-dose 2, and pre-dose 3 were collected 2 months apart and were obtained prior to dose 1, 2 and 3 respectively. Bacille Calmette Guerin (BCG) recipients exhibited greater IFN-γ responses to MTB lysate at multiple timepoints after dose 3. Graphs depict individual data points along with median values for all subjects at that timepoint. Gray-shaded circles represent IFN-γ responses to DAR-901 and black-shaded circles IFN-γ responses to BCG. B and BCG, bacille Calmette Guerin 1-8x10^6^ organisms in 0.1 mL; D or DAR, DAR-901 1 mg dose; S, saline. (TIFF) [file pone.0175215.s002.tiff]
