# Supplementary material for: Safety and immunogenicity of an inactivated whole cell tuberculosis vaccine booster in adults primed with BCG: A randomized, controlled trial of DAR-901
Source: PLoS One. 2017 May 12;12(5):e0175215. doi: 10.1371/journal.pone.0175215 (PMC5429024; doi:10.1371/journal.pone.0175215)
Supplement: S2 File — (PDF) [file pone.0175215.s004.pdf]

1. PROTOCOL DAR-901-MDES

**Study Title**	A Phase 1, Randomized, Placebo-Controlled, Double-Blind, Multiple-Dose, Dose-Ranging Study of the Safety and Immunogenicity of DAR-901, a Killed, Non-Tuberculous Mycobacterial Vaccine, in HIV-negative and HIV-positive Adults Who Have Previously Received BCG
**Investigational Drug:**	DAR-901
**IND Number:**	15838
**EudraCT**	not applicable
**Sponsor:**	C. Fordham von Reyn MD<br>Professor of Medicine<br>Infectious Disease and International Health<br>Geisel School of Medicine at Dartmouth<br>Lebanon, NH 03756
**Collaborator**	Aeras
**Protocol Number:**	DAR-901-MDES
**Protocol Version:**	4.1
**Date:**	30 March 2015

Statement of Confidentiality

This document contains the Sponsor's privileged or confidential information and is provided to you as an investigator, potential investigator or consultant, solely for review by you, your staff and applicable institutional review board(s). The information is not to be disclosed to others without written authorization from the Sponsor.

Protocol Approval Page

Study Title A Phase 1, Randomized, Placebo-Controlled, Double-Blind, Multiple-Dose, Dose-Ranging Study of the Safety and Immunogenicity of DAR-901, a Killed, Non-Tuberculous Mycobacterial Vaccine, in HIV-negative and HIV-positive Adults Who Have Previously Received BCG

Protocol Number: DAR-001-MDES

Protocol Version: 4.1

Date: 30 March 2015

Approved for the Sponsor by:

C. Fordham Von Reyn, M.D.
Dartmouth College

February 27, 2015

Signature Date

Revision History

Ver. No.	Date	Comment
1.0	20 December 2013	Submission to IRB
2.0	27 December 2013	Technical corrections; addition of memory aid diary and phone script
3.0	28 February 2014	Technical corrections re HIV testing
4.0	27 February 2015	Changes in A4, B1 and B2 cohorts
4.1	30 February 2015	Addition of repeat IGRA after Dose 3

2. PROTOCOL SYNOPSIS

2.1 Protocol Information

**Protocol Number:**	DAR-901-MDES
**Protocol Title**	A Phase 1, Randomized, Placebo-Controlled, Double-Blind, Multiple-Dose, Dose-Ranging Study of the Safety and Immunogenicity of DAR-901, a Killed, Non-Tuberculous Mycobacterial Vaccine, in HIV-negative and HIV-positive Adults Who Have Previously Received BCG
**Sponsor:**	C. Fordham von Reyn, MD
**Name of Finished Product**	DAR-901 for Injection
**Name of Active Ingredient**	DAR-901
**Phase of Development**	1
**Indication (Target)**	Prevention of tuberculosis in HIV-negative and -positive adolescents and adults, including persons with latent tuberculosis, previously vaccinated with BCG.
**Number of Subjects**	70 in planned dose groups
**Number of Sites**	One site in US (Dartmouth)

2.2 Study Objectives

2.2.1 Primary Objective

To evaluate the safety and tolerability of multiple doses of DAR-901, at different dose levels, administered to healthy HIV-negative and –positive adults by intradermal injection.

2.2.2 Secondary Objectives

To characterize the immunogenicity of multiple doses of DAR-901 at different dose levels, with emphasis on the cytokine responses to DAR-901 soniciate (i.e., IFN-γ secretion and Elispot).

2.3 Rationale for the Current Study

Development of an improved vaccine for the prevention of tuberculosis in both HIV-negative and HIV-positive persons is a major international health priority. In countries with endemic tuberculosis, BCG (bacillus Calmette-Guerin) is almost universally administered in childhood . However, in HIV-negative adolescents and young adults resistance to active infection wanes and in those co-infected with HIV the risk of disease is markedly increased. A major current emphasis is the development of a vaccine that would enhance BCG-induced immunity in both these populations. DAR-901 represents a candidate vaccine designed to fulfill this "prime-boost" strategy in HIV-negative persons, as well as being safe in those co-infected HIV or otherwise immunocompromised.

DAR-901 is a heat-inactivated, whole-cell vaccine derived from an environmental non-tuberculous mycobacterium. It represents a new manufacturing method (see details below for the prior product designated SRL 172. In previous studies, we demonstrated the safety, immunogenicity, and efficacy of a 5-dose series of SRL 172. Phase I safety studies with SRL 172 were conducted in HIV-negative adults and HIV-positive adults and children in the United States, and Phase II safety and immunogenicity studies in HIV-positive adults in Finland in and Zambia. An NIH-sponsored Phase III efficacy trial of SRL 172 was initiated in Tanzania in 2001 (hereafter referred to as the "DarDar Trial"). A total of 2013 HIV subjects with prior BCG were randomized 1:1 to receive vaccine (1 mg in 0.1 mL) or placebo (buffered saline alone) administered intradermally in the deltoid at 0, 2, 4, 6, and 12 months. Subjects were followed every 3 months for the development of tuberculosis. The vaccine was safe and well-tolerated with minimal local reactions, 0.3% vaccine site sterile abscesses, and 0.4% self-limited generalized rashes. Compared to placebo recipients, vaccine recipients showed significant increases in

IFN-γ responses to the vaccine antigen and significant increases in antibody to lipoarabinomannan (LAM). In 2008 the trial was stopped after the DSMB concluded that SRL 172 had shown significant protection against the secondary endpoint of active tuberculosis (defined as "definite" tuberculosis supported by smear or culture evidence of disease).

SRL 172 was prepared using agar-grown organisms, a manufacturing method that scaled poorly. A new broth-based manufacturing process was developed at Aeras, starting from the Master Cell Bank for SRL 172. The broth-produced product, now designated DAR-901, has completed non-clinical immunogenicity and toxicology studies (detailed in Section 5.3) and is now advancing to clinical trials. Note that GMP-grade SRL 172 for human use is no longer available.

2.4 Study Design

This is a Phase 1, randomized, controlled, double-blind, multiple-dose, dose-ranging study of DAR-901 to be conducted in HIV-negative and HIV-positive adults previously vaccinated with BCG. The goals of the trial are to determine the safety, tolerability, and immunogenicity of multiple doses of the vaccine at different dose levels, ranging from 0.1 to 1 mg. The maximum dose level (1 mg) corresponds to the dose of SRL 172 used in the successful DarDar trial. A dose of 0.3 mg DAR-901 provided maximal immunogenicity in mice; the 0.1 mg dose is included to provide further dose response data and assure a safe starting dose level.

Doses will be administered by intradermal injections in the deltoid area at 0, 2, and 4 months (Day 1, Day 53-67, and Day 113 to 127, respectively). In the Phase 2 study of SRL 172 conducted in Finland, this regimen showed significant immunogenicity. The intervals between injections and route of administration are consistent with all previous studies of SRL 172.

Table 2-1 displays the dose groups by HIV status and dose level (mg) per injection. All cohorts include placebo (saline) controls; in addition, the HIV-negative cohorts (A1–A3) include positive controls (a single dose of BCG as Dose #3, with saline at preceding doses).

Ford von Reyn 5/31/15 10:25 AM
Deleted: Table 2-1

Table 2-1. Dose Groups by HIV Status, IGRA status, and Dose Level

Dose Group	HIV Status	IGRA status	DAR-901 (Dose Level)	DAR-901 (N)	BCG (N)	Placebo (N)	Total
A1	Neg	Neg	0.1 mg	10	3	3	16
A2	Neg	Neg	0.3 mg	10	3	3	16
A3	Neg	Neg	1 mg	10	3	3	16
A4	Neg	Pos	TBD	4-6*	-	0	4-6
B1	Pos	Neg	TBD	5-8*	–	-	5-8
B2	Pos	Pos	TBD	5-8*	–	-	5-8
**Total**				**44-52**	**9**	**9**	**62-70***

TBD, to be determined. The dose level will be selected based on the preceding experience within the protocol as detailed in text.
* Ranges represent minimum and maximum number of subjects per cohort.

All subjects will be screened by Interferon-gamma release assay (IGRA; QuantiFERON®-TB Gold, Cellestis) for evidence of latent TB infection (LTBI). IGRA-positive subjects will be excluded from the initial dose escalation cohorts (A1–A3).

Based on the results obtained in the HIV-negative dose-escalation, a target dose level will be selected and administered to one additional HIV-negative cohort:

- Cohort A4: HIV-negative, IGRA-positive subjects; open label.

In addition, the protocol provides for administering the target dose to two cohorts of HIV-positive subjects, contingent on assessment of the vaccine's safety, tolerability and immunogenicity in the HIV-negative cohorts and the availability of resources:

- Cohort B1: HIV-positive, IGRA-negative subjects; open label.

- Cohort B2: HIV-positive, IGRA-positive subject; open label.

2.4.1 Enrollment Process

As part of the overall risk management plan (see below for additional provisions), enrollment will proceed as follows:

- The study will start with dose group A1 (HIV-negative, IGRA-negative; 0.1 mg per dose).
- The enrollment procedure comprises the following steps:
  - The first subject will be dosed with vaccine (Day 1) open-label, observed for 1 hr, and examined on Day 3 (~48 hr post-dosing). If there have been no significant safety-related events, enrollment will proceed.
  - For the remaining 15 subjects in the dose group, treatment will be assigned by randomization for A1-A3 and will be open label for A4, B1 and B2
  - A maximum of five subjects may be randomized per calendar day.
- After all subjects in the dose group have completed Study Day 7 follow-up visit, all available data will be reviewed by the Dose Review Committee (DRC; see Section 7.7 for details). Escalation to the next dose level will be contingent on their recommendation.
- If the DRC approves dose-escalation, then enrollment in dose group A2 will commence, using the same process as described for group A1. Similarly, a dose-escalation review will be conducted after all group A2 subjects complete Day 7 follow-up visit; and, if approved, enrollment in dose group A3 will proceed as detailed above.
- After all subjects in dose group A3 have completed the 7d safety evaluation after dose #3 the available safety data including injection site reactions will be conducted by the DRC, supplemented by the PI and, possibly, additional consultants with relevant expertise (e.g., immunology, tuberculosis).
- If the DRC concludes that the A3 dose level of 1 mg is safe then dose group A4 (HIV-negative, IGRA-positive) will be enrolled and administered the 1 mg dose level . If the DRC determines that the 1 mg dose is not safe then they will recommend one of the lower dose levels from A1 or A2 for A4. Subjects may be enrolled as available, up to a maximum of four subjects per calendar day.
- The dose selection review will also assess the vaccine's safety with regard to its administration to HIV-positive subjects. Progression to Part B will be contingent on that review and on the availability of resources.
- Dose group B1 will comprise HIV-positive, IGRA-negative subjects scheduled to receive DAR-901 at the same dose as the A4 cohort. This dose group will be enrolled using the same dose procedure steps detailed above for dose group A4.
- Dose group B2 will comprise HIV-positive, IGRA-positive subjects scheduled to receive DAR-901 at the same dose as the A4 cohort.. Enrollment in this dose group may commence following a satisfactory Day 3 safety check for the first subject in dose group A4. Dose group B2 will be enrolled using the same procedure steps detailed above for group A4.

2.4.2 Risk Management

The subjects in this Phase 1 trial are not expected to benefit from the treatment.

In addition to the staggered enrollment and dose escalation procedures detailed above, the following steps have been taken to minimize the risk of participation in this study.

2.4.2.1 Stratification by IGRA

As part of the screening procedures all subjects will have an IGRA to determine prior exposure to TB.

- Subjects with negative IGRA may be enrolled in the cohorts A1, A2, A3 and B1.

- Subjects with an IGRA reported as "indeterminate" (as defined by the manufacturer) will not be enrolled in the study and will be provided the IGRA result and referred to their personal physician.
- Subjects with a positive IGRA may be enrolled in cohorts A4 or B2, subject to the following conditions.
  - Prior to enrollment all IGRA-positive subjects will have chest x-ray and medical evaluation to exclude signs or symptoms of active TB.
  - Subjects currently taking preventive therapy for latent TB, or intending to start it imminently, will not be enrolled (HIV positive subjects who are IGRA positive will have all been offered preventive therapy by their primary physicians).

2.4.2.2 Dosing procedures

- All doses of study treatment (DAR-901, BCG, and placebo) will be prepared by a research pharmacist.
- All intradermal injections of study treatment in dose groups A1, A2 and A3will be administered by a blinded research nurse who will not participate in other clinical study activities. All evaluations will be performed by blinded study personnel not involved in preparation or administration of study treatment.
- Intradermal injections of DAR-901 in open label dose groups A4, B1 and B2 will be performed by the main study nurse
- All subjects will be observed for at least 30 minutes following all injections.
- The maximum dose is based on prior experience in >1000 adults with SRL-172, a killed mycobacterial vaccine prepared from the same strain. As noted above, there were 0.3% injection site sterile abscesses and 0.4% self-limited generalized rashes (see Table 5-5).

Ford von Reyn 5/31/15 10:25 AM
Deleted: Table 5-5

2.4.2.3 Adverse event monitoring

- At all visits subjects will be asked about adverse events and concomitant medications and a detailed injection site assessment will be performed.
- Between scheduled visits, subjects will be monitored by phone contacts twice weekly through +day 28 post-injection. Subjects will be provided a thermometer to measure body temperature and a ruler to measure injection site reactions. To assure accurate reporting at the scheduled phone calls, subjects will be provided a memory-aid diary to record findings and symptoms daily.
- Digital photographs will be obtained of injection site reactions that include skin breakdown, pustules or abscess
- Subjects whose clinical course suggests secondary complications, e.g., sterile or infected abscesses, will be asked to return to the clinic for evaluation by the investigator.
- Safety laboratory tests will be performed as detailed below. Subjects with treatment-emergent abnormalities meeting predefined toxicity grades will have study treatment discontinued (see Section 7.5.1).
- Subjects will be discontinued from study treatment (see Section 7.6.1) if they experience a dose-limiting toxicity (DLT) event, either systemic, local to the injection site, or demonstrated on laboratory tests (see Section 7.5.1 for details).

2.4.2.4 Other risk management procedures

- For HIV-positive subjects data on compliance with anti-retroviral therapy will be collected at each visit.
- All HIV-positive subjects will have a baseline chest x-ray to exclude active tuberculosis.
- Given the exploratory nature of this study, the sample size is based not on power calculations but rather on prior Phase 1 experience indicating that cohorts of this size are the minimum required to provide sufficient data for safety, tolerability, immunogenicity, and dose response assessment for advancing the development process.

- The trial will be conducted in compliance with the protocol, GCP, and applicable human studies and regulatory requirements.

2.4.3 Study Structure

2.4.3.1 Screening through Day 74

- Table 2-2. Schedule of Study Events – Screening Through Day 74
- Screening will be done within 28 days before the first injection of study drug.
- Subjects will receive the first dose as assigned on Day 1.
- Subjects may be discharged from clinic following completion of scheduled procedures.
- Subjects will be seen in clinic for scheduled procedures 7 days after dosing.
- Subjects will be contacted by phone for adverse event monitoring, including injection site reactions, twice weekly thru +28 days after dosing.
- Dose #2 will be administered 60 ± 7 days after Dose #1.
- Subjects will be seen in clinic for scheduled procedures 7 days after dosing.
- Subjects will be contacted by phone for adverse event monitoring, including injection site reactions, twice weekly thru +28 days after dosing.

2.4.3.2 Dose #3 through End-of-Study

- Table 2-3. Schedule of Study Events – Dose #3 to End-of-Study Visit
- Dose #3 will be administered 120 ± 7 days after Dose #1.
- Subjects will be seen in clinic at 7, 28, and 56 days after dosing for scheduled procedures.
- Subjects will be contacted by phone for adverse event monitoring, including injection site reactions, twice weekly thru +28 days after dosing.
- The End-of-Study (EOS) visit, the final study event, is scheduled for 180 ± 7 days after Dose #3.
- Total duration of involvement is ~330 days (11 months).

2.4.4 Potential protocol adjustments

The protocol is written with flexibility to accommodate the dynamic nature of Phase 1 clinical trials. An additional cohort may be enrolled, either to repeat a dose level or to administer a dose below or between the levels specified. In no event will the proposed maximum exposures be exceeded.

2.5 Subject selection

2.5.1 Inclusion Criteria – HIV-negative Subjects

To be eligible for this study, a subject must meet ***all*** of the following inclusion criteria:

1. Be age 18 to 65 years, inclusive;
2. Have completed the informed consent procedure (see Section **15.3**), including signing and dating the informed consent form;
3. Have received BCG as documented by presence of a scar consistent with immunization or a contemporary medical record;
4. Have two negative ELISA assays for antibody to human immunodeficiency virus type 1 and 2 (HIV-1, HIV-2);
5. Female subjects must have a negative serum pregnancy test at screening and negative urine pregnancy test within 24 hr prior to each dose of study drug;
6. Female subjects of childbearing potential (see Section **8.3**) must agree to use effective birth control (contraception; see Section **8.3**) from Screening through the treatment period and for 3 months after the last injection of study drug.

2.5.2 Exclusion Criteria – HIV-negative Subjects

A person who meets ***any*** of the following exclusion criteria will ***not*** be enrolled in the study:

1. Is nursing;
2. Has body weight <50 kg;

Ford von Reyn 5/31/15 10:25 AM
Deleted: Table 2-2. Schedule of Study Events – Screening Through Day 74

Ford von Reyn 5/31/15 10:25 AM
Deleted: Table 2-3. Schedule of Study Events – Dose #3 to End-of-Study Visit

Ford von Reyn 5/31/15 10:25 AM
Deleted: 15.3

Ford von Reyn 5/31/15 10:26 AM
Formatted: Font:Bold

Ford von Reyn 5/31/15 10:26 AM
Formatted: Font:Bold

Ford von Reyn 5/31/15 10:25 AM
Deleted: 8.3

Ford von Reyn 5/31/15 10:25 AM
Deleted: 8.3

Ford von Reyn 5/31/15 10:26 AM
Formatted: Font:Bold

3. Has a history of active tuberculosis;
4. Has previously received another investigational vaccine against tuberculosis;
5. Has received any investigational drug during the prior three (3) months;
6. Has had surgery requiring general anesthesia during the prior three (3) months;
7. Has lost >500 ml of blood (by any process, including donation) during the prior three (3) months;
8. Has received systemic immune suppressive or stimulatory prescription drugs during the prior three (3) months;
9. Has participated in any biomedical research protocol during the prior one (1) month;
10. Has used any prescription or over-the-counter medication during the prior one (1) month, unless approved by both the Investigator and the Sponsor;
11. Has had an illness consistent with acute viral or bacterial infection within the prior (2) two weeks;
12. If IGRA-positive, has a chest x-ray with any findings consistent with active tuberculosis;
13. Has a positive test for hepatitis B surface antigen (HBsAg) or for antibody to hepatitis C virus (HCV) [HIV-negative subjects only; HIV-positive subjects are not excluded for prior hepatitis B or C]
14. Has confirmed results on a screening laboratory test (hematology, coagulation, chemistry, urinalysis) that represent Grade 1 or greater abnormalities (tests with abnormalities may be repeated once);
15. Has a calculated creatinine clearance of <80 mL/min (based on the Cockcroft-Gault equation);
16. Has, within the past 10 years, had evidence of or required treatment for cancer (except treated basal or squamous cell carcinoma of the skin, or cured cervical carcinoma-in-situ);
17. Has other significant medical disease (chronic or active within the past 6 months), including, but not limited to: cardiac disease (e.g., unstable angina, myocardial infarction, congestive heart failure, ventricular arrhythmia), uncontrolled seizure disorder, liver disease, autoimmune or antibody-mediated diseases (e.g., lupus, rheumatoid arthritis), organ transplantation, chronic infection, uncontrolled diabetes; diseases judged by the Investigator as not clinically significant or as fully resolved will be reviewed with the Medical Monitor;
18. Has clinically significant abnormal findings on vital signs or physical examination;
19. Is expected to have surgery requiring general anesthesia during the study period;
20. Is, in the judgment of the Investigator, not suitable to participate in this clinical study.

2.5.3 Additional / Alternative Inclusion Criteria for HIV-positive Subjects

1. Has a previously documented positive HIV-1 viral load;
2. Has been on a stable combination anti-retroviral therapy (ART) for the past three (3) months;
3. Has been enrolled in regular HIV care for the past year, including a documented clinic visit within the past eight (8) months.
4. If IGRA-positive, has completed prophylaxis for latent TB, has a contraindication to prophylaxis, or has refused prophylaxis treatment.

2.5.4 Additional / Alternative Exclusion Criteria for HIV-positive Subjects

1. Has a history of an AIDS-defining opportunistic infection;
2. Has had a CD4 count <200/uL at any time in the past year;
3. Has confirmed results on a screening laboratory test (hematology, coagulation, chemistry, urinalysis) that represent Grade 2 or greater abnormalities (tests with abnormalities may be repeated once).

2.6 Treatments

All treatments will be administered as scheduled by intradermal injection in the deltoid.

2.6.1 Investigational Treatment

Different doses of DAR-901 (see Table 2-1) administered in a dose volume of 0.1 mL (see Section 9.5).

Ford von Reyn 5/31/15 10:25 AM
Deleted: Table 2-1

2.6.2 Comparator treatment

Active comparator: Tice BCG, Organon, Teknika, 1-8x10^6 organisms in 0.1 mL.
Inactive comparator: 0.1 mL of Sterile Saline for Injection (placebo).

2.7 Assessments

2.7.1 Safety Assessments

The safety and tolerability of DAR-901 will be assessed using reported and observed adverse events and scheduled safety observations, including quantitative measurements of injection site reactions, digital photography of injection site reactions, patient-recorded temperatures, vital signs, physical examination, laboratory tests (hematology, chemistry, and coagulation), urinalysis.

2.7.2 Immunogenicity Assessments

The following assays will be performed using isolated fresh or fresh frozen peripheral blood mononuclear cells (PBMCs) or serum, as indicated, using antigens prepared in the laboratory and previously used in non-clinical immunogenicity studies. Additional assays may be developed and applied as long as the blood volume required does not exceed that specified in Table 2-4.

Ford von Reyn 5/31/15 10:25 AM
Deleted: Table 2-4

- Antigens:
  - Standard: DAR-901 sonicate; MTb lysate; Ag 85, ESAT
  - Exploratory: mycobacterial peptides specific to DAR-901 and BCG
  - Controls: positive (PHA, PMA/ionomycin, or SEB as appropriate); negative (saline or media).
- Assays - primary:
  - Antigen-specific IFN-γ production by PBMCs in vitro by IFN-γ ELISA (Dartmouth)
  - IFN-γ Elispot – PBMCs stimulated with antigen and assessed for IFN-γ secreting cells (Aeras)
- Assays - additional:
  - Serum antibody to lipoarabinomannan (LAM) by ELISA (Dartmouth)
  - Antigen-specific 17-color intracellular cytokine staining (ICS) assay (Aeras)
  - RNA expression assay (Aeras)
  - Mycobacterial growth inhibition assay (MGIA) on PBMCs (Aeras)

2.8 Statistical Analyses

2.8.1 Analysis Populations

Within each population (HIV-negative and –positive), analyses will be performed comparing subjects by dose level and by treatment assignment (DAR-901 vs. Placebo). Where appropriate, data for different dose levels may be pooled.

- *Safety Population* — all subjects who received at least one injection of study medication.
- *Immunogenicity Populations* — all subjects with evaluable immunogenicity data, based on protocol compliance, adequate numbers of samples, and successful sample assays.

2.8.2 Safety Analyses

Tabulation and descriptive statistics of adverse events, vital signs, physical examinations, and laboratory tests.

2.8.3 Efficacy Analyses

Not applicable

2.8.4 Immunogenicity Analyses

Data will be analyzed using descriptive statistics and graphical displays.

2.8.5 Determination of Sample Size

The size of the study is based on prior experience with Phase 1 vaccine studies and represents the number of patients that will permit preliminary evaluation of safety and tolerability (primary objective) and, thereby, support the advancement of the vaccine development program.

2.9 Schedules of Study Events

Table 2-2. Schedule of Study Events – Screening Through Day 74

Event / Evaluation[1] Dose #		#1							#2					
Day[2]	≤-28	1			+3[3]	+5[4]	+7[5]	+10 to +28[4]	53-67			+3,5[4]	+7	+10 to +28[4]
Hour		Pre	0	1					Pre	0	0.5			
Informed Consent	X													
Medical History	X													
Physical examination[6]	X	X												
Vital signs	X	X		X	X[3]		X		X		X		X	
Injection Site Assessment[7]					X[3]		X		X				X	
Phone Contact					X	X		X				X		X
Safety Laboratory Tests	X	X[20]							X					
HIV Monitoring Tests[8]	X	X[20]							X					
Urinalysis	X								X					
Pregnancy test (F only)	X	X							X					
IGRA test, Chest X-ray[9]	X													
Serology[10]	X													
Immunogenicity Studies		X[19]							X					
Dose administration			X							X				

Monitoring AE & Con Med	-------------------------- From Screening to End of Study Visit ------------------------------

[1] For details of study evaluations see Section 10.
[2] Day is relative to the calendar day of the first injection of study drug, designated Day 1; time (Hour) is relative to time of injection, designated 0 hr. "Pre" indicates within 2 hr prior to injection.
[3] Day 3 visit only for first subject in each cohort. All other subjects will be contacted by phone on Day 3.
[4] "+3", "+5", "+10 to +28" – indicates phone contacts for adverse event monitoring, including injection site reactions, to be performed on days 3 and 5 post-dosing and at least twice weekly through day 28 post-dosing.
[5] "+7" indicates visit to be performed 6 to 8 days after actual day of dosing.
[6] Physical exam includes weight and, at screening only, height.
[7] Assessment of injection site area comprises a structured examination of the injection site and measurements of any reaction (see Section 12.5).
[8] HIV monitoring tests – HIV viral load, CD4 counts; to be performed only on HIV-positive subjects
[9] IGRA will be performed on all subjects. All IGRA positive subjects will have a chest x-ray; see Section 2.4.2 for provisions regarding preventive therapy in those subjects.
[10] Serology includes hepatitis B surface antigen, antibody to hepatitis C virus, and antibody to HIV-1 and -2; see Section 10.3.3 for details.

Table 2-3. Schedule of Study Events – Dose #3 to End-of-Study Visit

Event / Evaluation[11] Dose#		#3							EOS[12]
Day[13]	113-127			+3,5[14]	+7[15]	+10 to +28[14]	+28[15]	+56	293-307
Hour	Pre	0	0.5						
Informed Consent									
Medical History									
Physical examination[16]	X						X		
Vital signs	X		X		X		X	X	X
Injection Site Assessment[17]					X		X	X	X
Phone contact				X		X			
Safety Laboratory Tests	X						X		
HIV Monitoring Tests[18]	X						X		
Urinalysis							X		
Pregnancy test (females only)	X						X		
IGRA test, Chest X-ray								(X)[21]	X[21]
Serology									
Immunogenicity Studies	X				X		X	X[19]	X
Dose # administration		X							

Monitoring AE & Con Med	From Screening to End of Study Visit

[11] For details of study evaluations see Section 10.
[12] EOS, End-of-Study visit
[13] Day is relative to the calendar day of the first injection of study drug, designated Day 1; time (Hour) is relative to time of injection, designated 0 hr. "Pre" indicates within 2 hr prior to injection.
[14] "+3", "+5", "+10 to +25" – indicates phone contacts for adverse event monitoring, including injection site reactions, to be performed on days 3 and 5 post-dosing and at least twice weekly between the visits scheduled for days 7 and 28 post-dosing.
[15] "+7", "+28", "+56" indicate visits to be performed 6 to 8 days, 26 to 30 days, and 54 to 60 days, respectively, after actual day of dosing.
[16] Physical exam includes weight and, at screening only, height.
[17] Assessment of injection site area comprises a structured examination of the injection site and measurements of any reaction (see Section 12.5).
[18] HIV monitoring tests – HIV viral load, CD4 counts; to be performed only on HIV-positive subjects
[19] Immune assays for HIV positive subjects are only performed at baseline and at Day 56 after dose #3
[20] Labs not repeated if subject dosed within 2 weeks of screening and has no intervening change in health status
[21] IGRA repeated at Day 56 or EOS visit for subjects in cohort A3 (3 mL blood)

Table 2-4. Estimation of Blood Volumes Required[19]

Event Day /Evaluation[20]	Vol (mL)	Scrn	1	53-67	113-127	+7	+28	+56	EOS 293-307	Approx Total Vol (mL)
Dose #			#1	#2	#3					
Safety Lab Tests	15	X	X	X	X		X		X	78
HIV Monitoring Tests	10	X	X	X	X		X			50
Pregnancy test (F only)[21]		X	X				X			0
Immunogenicity (cells)	40		X	X	X	X	X	X	X	280[22]
Immunogenicity (serum)	5		X	X	X	X	X	X	X	35[22]
RNA expression	3		X	X		X			X	12
Serology (Hep B, C, HIV)	10	X								10
**Total mL blood drawn**		35	73	73	70	48	70	45	51	**465**[22]

[19] The volumes shown are estimates; final volumes may vary, but will not be more than 15% greater than shown.
[20] For details of study evaluations see Section 10.
[21] Pregnancy test does not require additional blood volume.
[22] Limit of two immunogenicity assays for HIV positive subjects in cohorts B1 and B2 reduces total volume of phlebotomy by 225 mL to 237 mL

Table 2-5. Overall Study Timeline

**Cohort**	**A1**	**A2**	**A3**	**A4**	**B1**	**B2**
**HIV status**	Neg	Neg	Neg	Neg	**Pos**	**Pos**
**IGRA status**	Neg	Neg	Neg	**Pos**	Neg	**Pos**
**Dose Level**	**0.1 mg**	**0.3 mg**	**1 mg**	**TBD**	**TBD**	**TBD**
Study Week						
1	Dose #1					
2	7d Visit					
3	Safety review					
4		Dose #1				
5		7d Visit				
6		Safety review				
7			Dose #1			
8			7d Visit			
9	Dose #2					
12		Dose #2				
15			Dose #2			
17	Dose #3					
20		Dose #3				
23			Dose #3			
24						
25						
…26			Dose Safety Review			
27				Dose #1	Dose #1	
28				7d Visit	7d Visit	Dose #1
29				Safety review	Safety review	
30						
35				Dose #2	Dose #2	
36						Dose #2
38						
41	End of Study					
43				Dose #3	Dose #3	
…44		End of Study				Dose #3
46						
…47			End of Study			
...52						
…55						
…67				End of Study	End of Study	
…68						End of Study

TBD, dose for A4, B1, and B2 to be determined based on safety review; see text for details.

3. TABLE OF CONTENTS

Section **Page**

1. PROTOCOL DAR-901-MDES ... 1
2. PROTOCOL SYNOPSIS ... 3
2.1 Protocol Information ... 3
2.2 Study Objectives ... 3
2.2.1 Primary Objective ... 3
2.2.2 Secondary Objectives ... 3
2.3 Rationale for the Current Study ... 3
2.4 Study Design ... 4
2.4.1 Enrollment Process ... 5
2.4.2 Risk Management ... 5
2.4.3 Study Structure ... 7
2.4.4 Potential protocol adjustments ... 7
2.5 Subject selection ... 7
2.5.1 Inclusion Criteria – HIV-negative Subjects ... 7
2.5.2 Exclusion Criteria – HIV-negative Subjects ... 7
2.5.3 Additional / Alternative Inclusion Criteria for HIV-positive Subjects ... 8
2.5.4 Additional / Alternative Exclusion Criteria for HIV-positive Subjects ... 8
2.6 Treatments ... 8
2.6.1 Investigational Treatment ... 8
2.6.2 Comparator treatment ... 9
2.7 Assessments ... 9
2.7.1 Safety Assessments ... 9
2.7.2 Immunogenicity Assessments ... 9
2.8 Statistical Analyses ... 9
2.8.1 Analysis Populations ... 9
2.8.2 Safety Analyses ... 9
2.8.3 Efficacy Analyses ... 9
2.8.4 Immunogenicity Analyses ... 9
2.8.5 Determination of Sample Size ... 10
2.9 Schedules of Study Events ... 11
3. TABLE OF CONTENTS ... 15
4. ABBREVIATIONS ... 20
5. BACKGROUND ... 21
5.1 Rationale for Investigation of DAR-901 ... 21
5.2 Overview of DAR-901 ... 21
5.3 Nonclinical Studies Conducted with DAR-901 ... 21
5.3.1 Provenance. ... 21

5.3.2 Preparation. 22
5.3.3 Immunogenicity. 23
5.3.4 Toxicology. 24
5.4 Clinical Experience with DAR-901 25
5.5 Clinical Experience with Vaccines Derived from the Same Genotypic Strain 25
5.5.1 SRL 172, Study 001 26
5.5.2 SRL 172, Study 002 27
5.5.3 SRL 172, Study 003 27
5.5.4 SRL 172, Study 004 28
5.5.5 SRL 172, Study 005 29
5.5.6 SRL 172, Study 006 (DarDar Trial) 30
6. OBJECTIVES 34
6.1 Primary: 34
6.2 Secondary: 34
7. INVESTIGATIONAL PLAN 35
7.1 Overall Study Design 35
7.2 Rationale for Treatment Regimens 35
7.2.1 Dose Levels 35
7.2.2 Dose Number and Dosing Interval 35
7.3 Rationale for Study Design 35
7.4 Maximum Exposures and Maximum Number of Subjects 36
7.5 Definitions Applicable to Managing the Study 36
7.5.1 Definition of a Dose-Limiting Toxicity (DLT) Event 36
7.5.2 Definition of a Completed Patient 36
7.5.3 Definition of a Completed Dose Group 36
7.6 Procedures for Managing the Study 36
7.6.1 Discontinuing Study Treatment in Individual Patients 36
7.6.2 Replacement of Subjects 37
7.7 Dose Review Committee (DRC) 37
7.8 Discontinuation of the Study 37
8. SUBJECT SELECTION 38
8.1 Source of Subjects and Recruitment Methods 38
8.2 Subject Disclosures and Restrictions during the Conduct of the Study 38
8.3 Definitions 38
8.3.1 Non-childbearing potential 38
8.3.2 Effective birth control (contraception) methods 38
8.4 Subject Selection Criteria 38
8.4.1 Observed Variances 38
9. STUDY TREATMENTS 39

9.1 Study Treatments to be Administered ... 39
9.2 Identity of the Investigational Product ... 39
9.3 Preparation and Handling of DAR-901 for Injection by Dose Level ... 39
9.3.1 0.1 mg DAR-901 for Injection ... 39
9.3.2 0.3 mg DAR-901 for Injection ... 39
9.3.3 1 mg DAR-901 for Injection ... 40
9.4 Reference and Blinding Therapy ... 40
9.5 Administration of Study Treatments ... 40
9.5.1 Variances in Dose Administration Schedule ... 40
9.6 Method of Assigning Patients to Treatment Groups ... 40
9.7 Selection of Dose Levels in the Study ... 40
9.8 Selection of Dose Number and Interval ... 40
9.9 Blinding ... 40
9.9.1 Procedures for Unblinding Individual Patients during the Study ... 40
9.10 Prior and Concomitant Therapy ... 41
9.11 Treatment Compliance ... 41
9.12 Accountability of Investigational Drug Supplies ... 41
10. STUDY EVALUATIONS ... 42
10.1 Medical History and Physical Exam ... 42
10.2 Vital Signs ... 42
10.3 Laboratory Studies ... 42
10.3.1 Safety Laboratory Tests ... 42
10.3.2 HIV Monitoring Tests ... 42
10.3.3 Screening Serologic Tests ... 42
10.3.4 Pregnancy Tests ... 43
10.4 Reporting of Safety Laboratory Tests ... 43
10.4.1 Repeating Abnormal Laboratory Tests ... 43
10.5 Immunogenicity Assessments ... 43
10.5.1 Preparation and storage of Peripheral Blood Mononuclear Cells (PBMCs) ... 43
11. STUDY EVENTS ... 45
11.1 Screening ... 45
11.2 End-of-Study Visits For Subjects who Terminate Prematurely ... 45
12. SAFETY EVALUATIONS ... 46
12.1 Definitions ... 46
12.1.1 Adverse Event (AE) ... 46
12.1.2 Pregnancy ... 46
12.1.3 Serious Adverse Event (SAE) ... 46
12.1.4 Suspected, Unexpected Serious Adverse Reaction (SUSAR) ... 47
12.1.5 Investigator Assessment of Safety Laboratory Tests ... 47

12.2 Collecting and Recording Adverse Events ... 47
12.3 Characterizing Adverse Events ... 48
12.3.1 Description of Event ... 48
12.3.2 Date and Time of Onset ... 48
12.3.3 Relationship to Study Drug ... 48
12.3.4 Intensity (Severity) ... 49
12.3.5 Management of Study Drug ... 50
12.3.6 Actions Taken for Management of AE ... 50
12.3.7 Outcome ... 50
12.3.8 Date and Time of Outcome ... 51
12.4 Reporting of Serious Adverse Events ... 51
12.4.1 Where to Report SAEs ... 51
12.4.2 Procedures for Reporting SAEs to the Sponsor ... 51
12.4.3 Requirements for Expedited and Periodic Reporting of Adverse Events ... 52
12.4.4 Notification of SAEs to the Investigator by the Sponsor ... 52
12.5 Sponsor Guidance for Grading of Injection Site Reactions ... 52
12.5.1 Definitions of the Most Commonly Expected Systemic Vaccine-related Symptoms ... 52
12.5.2 Definitions of the Most Commonly Expected Injection Site Symptoms ... 52
12.5.3 Definitions of the Most Commonly Expected Injection Site Findings ... 52
12.5.4 Capturing and Grading Injection Site Reactions and Other Post-Dosing Events ... 53
12.5.5 Subject Monitoring of Injection Site Reactions ... 54
12.6 Grading of Specific Laboratory Safety Tests for Reporting and Analysis ... 54
13. DATA QUALITY ASSURANCE ... 56
13.1 Compliance ... 56
13.2 Training and Qualifications of Site Personnel ... 56
13.3 General Procedures for Completing Data Collection ... 56
13.4 Case Report Forms ... 56
13.5 Source Documents ... 56
13.6 Protocol Deviations ... 57
13.7 External Review of the Study Conduct at Participating Sites ... 57
13.8 Resolution of Deficiencies ... 58
13.9 Study Closeout ... 58
13.10 Record Retention ... 58
13.11 Data Management ... 59
14. STATISTICAL METHODS ... 60
14.1 Power and Sample Size ... 60
14.2 Analysis Populations ... 60
14.3 Statistical Methods ... 60
14.4 Safety Assessments ... 60
14.5 Immunogenicity Analyses ... 60

14.5.1 IFN-γ assay by ELISA ... 60
14.5.2 ELISpot Assay ... 61
14.5.3 Intracellular Cytokine Staining (ICS) ... 61
14.5.4 Mycobacterial Growth Inhibition Assay ... 61
14.5.5 LAM assay ... 62
14.6 Identification of Study Event Days and Times ... 62
14.7 Handling Missing Data ... 62
14.8 Changes in the Planned Analyses ... 62
15. ETHICAL CONSIDERATIONS ... 63
15.1 Independent Ethics Committee (IEC) ... 63
15.2 Ethical Conduct of the Study ... 63
15.3 Subject Information and Consent ... 63
15.3.1 Obtaining Informed Consent from Subjects Who Are Not Literate ... 64
15.3.2 Special Informed Consent Situations Not Applicable to This Protocol ... 64
15.4 Protection of Subject Information ... 64
16. STUDY ADMINISTRATION ... 65
16.1 Registration of Study ... 65
16.2 Changes in the Conduct of the Study ... 65
16.3 Confidentiality ... 65
16.4 Financial Disclosure ... 65
16.5 Communication (Publication) Policies ... 65
16.5.1 Authorship and Acknowledgement ... 66
17. CONTACT INFORMATION ... 67
18. REFERENCES ... 68
19. INVESTIGATOR AGREEMENT ... 70

4. ABBREVIATIONS

Abbreviation	Definition
ADL	activities of daily living
AE	adverse event
ALT	alanine amino-transferase
ANC	absolute neutrophil count
aPTT	activated partial thromboplastin time
AST	aspartate amino-transferase
CRF	case report form
CRO	Contract Research Organization
DNA	deoxyribonucleic acid
ECG	Electrocardiogram
FDA	Food and Drug Administration (U.S.)
GCP	Good Clinical Practice
hCG	human chorionic gonadotropin
HIPAA	Health Insurance Portability and Accountability Act
HIV	human immunodeficiency virus
HPF	high-power field
HR	heart rate
ICF	informed consent form
ICH	International Conference on Harmonisation
ICMJE	International Committee of Medical Journal Editors
IFN	Interferon
IL	Interleukin
INR	international normalized ratio
IP-10	interferon-inducible protein 10
IRB	institutional review board
LDH	lactate dehydrogenase
LFT	liver function test
MCB	master cell bank
MCH	mean corpuscular hemoglobin
MCHC	mean corpuscular hemoglobin concentration
MCV	mean corpuscular volume
PBMC	peripheral blood mononuclear cell
PI	principal investigator
PK	pharmacokinetics
PPT	partial prothrombin time
PT	prothrombin time
RANTES	regulated on activation, normal T cell expressed and secreted (a cytokine)
RBC	red blood cell
rIFN-α	recombinant interferon alpha
SAE	serious adverse event
SGOT	serum glutamic oxaloacetic transaminase
SGPT	serum glutamic pyruvic transaminase
TNF	tumor necrosis factor
ULN	upper limit of normal
WBC	white blood cell

5. BACKGROUND

5.1 Rationale for Investigation of DAR-901

Development of an improved vaccine for the prevention of tuberculosis is a major international health priority. BCG (bacillus Calmette-Guerin) is almost universally administered in childhood in countries with endemic tuberculosis. However, resistance to active infection wanes in adolescents and young adults and the risk of disease is markedly increased in HIV-infected persons. A major current emphasis is the development of a vaccine that would enhance immunity in these populations. DAR-901 represents a candidate vaccine designed to fulfill this "prime-boost" strategy with minimal risk in immunecompromised persons.

In previous studies, we have demonstrated the safety, immunogenicity, and efficacy of a 5-dose series of SRL 172, a whole-cell vaccine derived from a environmental non-tuberculous mycobacterium and prepared using organisms grown on agar and then heat-inactivated. Phase I safety studies were conducted in HIV-negative adults and HIV-positive adults and children in the United States, and Phase II safety and immunogenicity studies in HIV-positive adults in Zambia and in Finland.

An NIH-sponsored Phase III efficacy trial of SRL 172 was initiated in Tanzania in 2001 (hereafter referred to as the "DarDar Trial")[1]. A total of 2013 HIV-positive subjects with prior BCG were randomized 1:1 to receive a 5-dose series of vaccine (1 mg in 0.1 mL) or placebo (buffered saline alone) administered intradermal in the deltoid) at 0, 2, 4, 6, and 12 months. Subjects were followed every 3 months for the development of tuberculosis. The vaccine was safe and well-tolerated with modest local reactions; the incidence of sterile abscesses at the vaccine site was 0.3% and of self-limited, generalized rashes, 0.4%. Compared to placebo recipients, vaccine recipients showed significant increases in IFN-γ responses to the vaccine antigen and significant increases in antibody to lipoarabinomannan (LAM). In 2008 the trial was stopped after the DSMB concluded that SRL 172 had shown significant protection against active tuberculosis (defined as "definite" tuberculosis supported by smear or culture evidence of infection).

The agar-based manufacturing method used to prepare SRL 172 scaled poorly. A new broth-based manufacturing process was developed at Aeras, starting from the Master Cell Bank for SRL 172. The broth-produced product, now designated DAR-901, is similarly a heat-inactivated, whole cell preparation. DAR-901 has completed non-clinical immunogenicity and toxicology studies (detailed in Section 5.3) and is now advanced to clinical trials. Note that GMP-grade SRL 172 for human use is no longer available.

5.2 Overview of DAR-901

5.3 Nonclinical Studies Conducted with DAR-901

5.3.1 Provenance.

DAR-901 is prepared from GMP stocks used to prepare SRL 172; the designation has been changed because DAR-901 is prepared with a different growth process (broth) by a new manufacturer (Aeras, Rockville, MD). The provenance of DAR-901 is summarized below.

- A strain of non-tuberculous mycobacteria was cultured from soil in Uganda by Dr. John Stanford in 1971 and a stable, rough variant was isolated on subculture [2].
- The rough variant was deposited in 1984 with National Culture Type Collection as NCTC 11659.
- An aliquot of NCTC 11659 was used to prepare a Master Cell Bank, designated MS/01/93 (Public Health England, Porton Down, UK) under a contract with SR Pharma, London, UK.
- MCB MS/01/93 was used by the Centre for Applied Microbiology and Research (CAMR) (Salisbury, UK) to prepare SRL 172 Clinical Trial Material, an agar-grown, heat-inactivated, whole cell vaccine for SR Pharma (London, UK). This was the material investigated in the "DarDar" trial (C. Ford von Reyn, Principal Investigator)..

- MCB MS/01/93 was used by Eden Biodesign Ltd. (Liverpool, UK) to prepare MCB lot C001-07-001.
- Aliquots of MCB lot C001-07-001 were provided to Aeras (Rockville, MD) and used to prepare MCB lot 12-107M-001.
- MCB 12-107M-001 was used by Aeras to prepare DAR-901 Drug Product, a broth-grown, heat-inactivated, whole-cell vaccine, lot 12-107F-001.

Aeras performed 16S rRNA gene sequencing of MCB lot #12-107M-00 and confirmed it was 100% identical to MCB MS/01/93, MCB C001-07-001, and SRL 172 CTM, which was used in the DarDar trial.

The 16s rRNA gene sequencing indicates that the MS/01/93 MCB, C001-07-001 MCB, and 12-107M-001 MCB have >99.6% identity to the reference 16S rRNA sequence for *Mycobacteria obuense* and <95% with *M. vaccae*.

5.3.2 Preparation.

DAR-901 is manufactured by Aeras by fermentation of the bacterial strain, heat inactivation and distribution of bulk drug substance as a 0.3-0.4 mL suspension into 2 mL vials at a concentration of 1 mg/mL. The manufacturing process is summarized below:

Figure 5-1. DAR-901 Bulk Drug Substance Manufacturing Flow Diagram

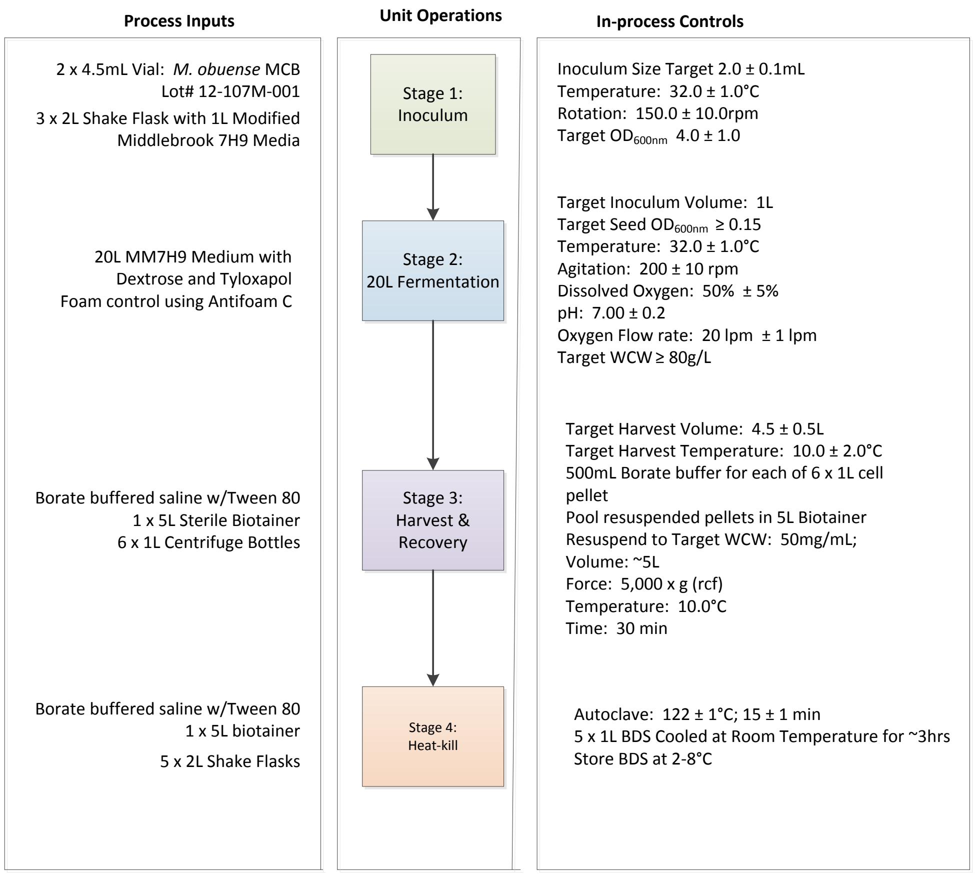

5.3.3 Immunogenicity.

Objective. To determine the immunogenicity of a 3-administration series of DAR-901 at different dose levels in 2 species of mice. The DAR-901 used in the immunogenicity studies was prepared as per the procedures outlined in the vaccine preparation section above (5.3.2)

Design. A total of 105 C57BL/6 and 105 BALB/c mice received 3 administrations of intradermal DAR-901 at 2 week intervals at the following doses (mg): .01, .03, .1, .3, 1.0, 2.5. A total of 70 animals were sacrificed 2 weeks after each dose. Spleens and blood were collected for the following assays: in vitro stimulation assay for IFN-γ, ELISpot for IFN-γ and antibody ELISA. Antigens included DAR-901 sonicate and CFP; SRL-172 lysate and CFP; *M. tuberculosis* lysate and CFP; *M. tuberculosis* PPD and hsp. Antibody to the same antigens and to *M. tuberculosis* lipoarabinomannan (LAM) was also assayed at a single time point 2 weeks after dose 3.

Results. IFN-γ responses to the vaccine antigen, DAR-901 sonicate, were induced after dose 1 and increased progressively with doses 2 and 3 in both mouse species with both IFN-γ assays. Maximum responses were typically observed at the 0.3 mg dose. IFN-γ responses to the DAR-901 CFP followed a

similar pattern. Similar, though lesser magnitude responses were induced by SRL-172. IFN-γ responses to *M. tuberculosis* lysate and CFP were also induced, most notably in BALB/c mice.

Figure 5-2. IFN-γ responses based on in vitro stimulation after 3 immunizations at various dose levels.

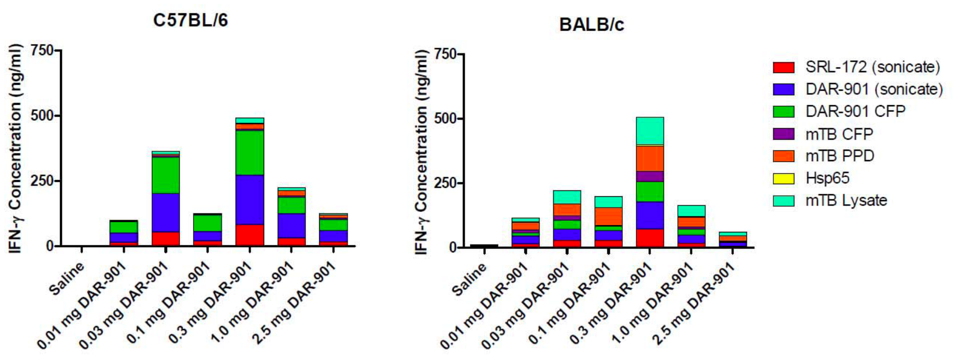

Antibody was induced to DAR-901 sonicate, SRL-172 lysate and DAR-901 CFP. There were no detectable responses against LAM.

Conclusions

Mycobacteria-naïve mice showed both cellular and humoral immune responses to a 3-administration series of DAR-901, with increasing responses after each of 3 doses. Responses were maximal at a dose of 0.3 mg.

5.3.4 Toxicology.

Objective. A GLP repeat dose intradermal toxicity study conducted in C57BL/6NHsd mice. The purpose of this study was to investigate the local and systemic toxicity and immunogenicity of DAR-901. The toxicology study exposure exceeded the schedule proposed for the human dose escalation study as follows:

- Number of doses: five (5) in the toxicology study vs three (3) in the human trial
- Dose level: 2.5 mg in the toxicology study vs. in the human trial, the starting dose is 0.1 mg and the maximum dose is 1 mg
- Dose volume per total subject weight: 0.05 mL per 20 gram mouse in toxicology study vs. 0.1 mL per 70 kilogram (average) adult in the human trial
- dosing interval intensity: two (2) week intervals in toxicology study vs eight (8) week intervals in the human trial.

Methods. Two (2) groups of C57BL/6NHsd, specific pathogen free mice (30/sex/group) received a 50 uL intradermal injection of test (Group 2) or control (formulation buffer; Group 1) article on Days 0, 14, 28, 42, and 56. At each interval 2.5 mg of test article was injected in a separate injection site in the back. The DAR-901 used in the repeat dose intradermal toxicity studies was prepared as per the procedures outlined in the vaccine preparation section above (5.3.2)

Toxicity was assessed based on clinical observations, physical exams, administration site evaluation, body weights, hematology (5/sex/group/interval), coagulation (5/sex/group/interval), serum chemistry (5/sex/group/interval), organ weights, and macroscopic and microscopic pathology evaluation. Inflammatory response was assessed by measuring serum fibrinogen levels. Local (injection site) reactions were evaluated using a modified Draize score (Draize et al., 1944). Thirty animals per sex per

group were sacrificed at Day 59 (End of Treatment Period) and the remaining animals (thirty per sex per group) were sacrificed at Day 70 (End of Recovery Period).

Results. Confirmation of vaccine take was performed using ELISA for serum immunoglobulin (IgG) to culture filtrate protein (CFP) from the mycobacterial strain from which DAR-901 was prepared. Serology data confirmed induction of an immune response against the test article in Group 2 animals.

Adverse observations related to the test article were restricted to changes at the injection site. These were similar in both treated males and females. The changes include:

- Erythema and/or edema – seen at the majority of injection sites beginning 1 to 2 days post-injection and typically lasting for 4 to 6 days.
- Induration – seen at the majority of injection sites, typically with delayed onset at the sites injected for doses 1, 2, and 3 (days 0, 14, and 28, respectively), with induration first noted at the exams on day 47 to 49. For doses 4 and 5 (days 42 and 56, respectively), the onset of induration of induration was more rapid and was noted as early as 1 week post-injection. Thus, as the study progressed, induration was frequently noted at locations separate from the most recent injection site. The size of the induration was typically 1 to 10 mm; occasional larger induration reactions appeared to represent the confluence of induration at two adjacent injection sites.
- Ulceration – a subset of injection sites developed ulceration, which was described as minimal or moderate except in three animals where ulceration was described as severe at one to three injection sites each.
  - Overall, 20 (33%) of 60 test-article animals had ulceration at one or more injections sites during the course of the study. Most ulcerations resolved by the time of sacrifice.
  - Among 40 animals sacrificed at day 60, 4 (10%) had ulceration at one to four injection sites; in three of the four animals the sites from dose 1 or 2 (day 0 and 14, respectively) were ulcerated.
  - Among the 20 animals sacrificed on day 70, 3 (15%) had ulceration at two or three injections sites; in all cases the involved sites represented doses 3, 4 or 5 (days 28, 42, and 56, respectively).

Minor changes in clinical pathology were noted, consistent with an inflammatory response, but none were considered adverse. Microscopic examination confirmed an inflammatory response at the injection site in animals with cutaneous ulceration.

Conclusions. Injection site reactions were observed in animals who received five (5) administrations of DAR-901 at a dose 2.5x higher than the maximum human dose proposed in Phase I dose escalation study. Most reactions were resolving at the time of necropsy. The reactions at early injection sites were delayed and were more frequent and intense after administration of multiple doses, consistent with the induction of a strong cutaneous delayed hypersensitivity response after multiple immunizations.

These reactions are consistent with observations in human studies involving injection of live or heat-inactivated mycobacterial vaccines. The proposed exposure in the proposed human trial is appreciably less intense with respect to dose, interval and total exposure. Specifically, the starting dose in the human dose escalation trial represents 4% of the dose used in the animal toxicity study; the interval between doses is increased 4-fold; and the maximum number of doses is three. There is provision for careful and regular assessment of injection site reactions both local and systemic, after each dose.

5.4 Clinical Experience with DAR-901

This study will be the first clinical experience with DAR-901.

5.5 Clinical Experience with Vaccines Derived from the Same Genotypic Strain

As detailed in Section 5.3.1, SRL 172 was a heat-inactivated, whole-cell vaccine derived from a rough variant of an environmental mycobacterium. Lot MV 001 of SRL 172 was used for a series of six human studies described below (Table 5-1). All doses were administered as an 0.1 mL intradermal injection over

Ford von Reyn 5/31/15 10:25 AM
Deleted: Table 5-1

the deltoid containing 1 mg SRL 172 in borate buffered saline (estimated to represent 10^9 colony forming units based on wet weight). All studies were investigator-initiated, conducted in accordance with applicable regulatory requirements, and published in peer-review journals as indicated.

Table 5-1. Studies conducted using SRL 172 (Lot MV 001)

Study Number	Title	Reference
001	A Phase 1 Study of Five Doses of SRL 172 in Healthy Adults	3, 4
002	A Phase 1 Study of Five Doses of SRL 172 in HIV-infected Adults	5
003	A Phase 1 Randomized, Controlled, Phase 2 Study of Three Doses of SRL 172 in HIV-infected Children	6
004	A Phase 2 Open-Label, Controlled Study of Five Doses of SRL 172 in BCG-positive and BCG-negative, HIV-infected Adults in Zambia	7
005	A Phase 2 Randomized, Controlled Trial of Five Doses of SRL 172 in HIV-negative, BCG-positive and HIV- positive, BCG-positive Adults in Finland	8
006	A Phase 3, Randomized, Placebo-Controlled Trial of Five Doses of SRL 172 for Protection against Tuberculosis in BCG-primed, HIV-infected Adults in Tanzania (the DarDar Trial)	1

Table 5-2. Key characteristics of studies conducted using SRL 172 (Lot MV 001)

Study Number	Period Conducted	HIV status	BCG status	N SRL 172[a]	N PLA	No. Doses	Dosing (months)
001	1994–1995	Neg	Neg[b]	10	–	5[c]	0, 2, 10, 16, 18
002	1995	Pos	Neg	12	–	5[d]	0, 2, 4, 25, 26
003[e]	1994–1997	Pos	Neg	23	12[f]	3	0, 2, 4
004	1996	Pos<br>Pos	Neg<br>Pos	11<br>11	11<br>11	5	0, 2, 4, 12, 16
005	1997	Neg<br>Pos	Pos<br>Pos	10<br>19	–<br>20[f]	5	0, 2, 4, 6, 12
006	2001–2008	Pos	Pos	1006	1007	5	0, 2, 4, 6, 12

a. In all adult studies the dose of SRL 172 was 1 mg / 0.1 mL administered intradermally in the deltoid (dose for children < 5 was 0.05 mL).
b. 1 subject was BCG-positive.
c. 3 subjects received only 3 doses.
d. 4 subjects received only 3 doses.
e. Study 003 was conducted in children (ages 6 mo to 13 yr). All other studies were in adults, ages 18 to 70 yr.
f. Controls received hepatitis B vaccine.

5.5.1 SRL 172, Study 001

Study overview:

- Subjects: 10 healthy (HIV-negative) adults, age 23-68, in US; 9 BCG negative, 1 BCG positive
- Dose schedule: 0, 2 and 10 months (10 subjects); 16 and 18 month (7 subjects)

Vaccine site reactions: Assessed at 2d, 14d, 2 months (photographs). Vaccine reactions noted over the first three doses (0, 2 and 4 months; 10 subjects) were:

- Erythema and induration: Noted in all subjects after each dose, maximal at 2 days. Median (range) induration at 2d post-dose were: dose one, 9 mm (6-25 mm), dose two, 8 mm (6-13 mm), dose three, 7 mm (4-17 mm).
- Drainage: Three subjects noted scant drainage: in subject 2, after doses one and two but not three; in subject 6 [BCG positive], after dose one [then withdrew because of pregnancy]; in subject 7, after doses two and three.

- Pain: “Sore arm” was reported by 2-5 subjects after each dose.

Other Safety Assessments:

- Temperature was recorded daily at home for 14 days after each dose and at study visits at 2d, 14d, 2 mos after each dose. All recorded temperatures were normal (<38.0°C)
- Laboratory: There was no clinically significant abnormal laboratory tests.
- Patient-reported adverse events: Patients were asked about interim and current symptoms at all visits. 1 feverish sensation and 1 mild malaise were reported after dose one; 1 headache, 1 feverishness and 1 malaise after dose two; 1 headache after dose three.

Three patients were lost to follow-up. Among the 7 patients who received additional doses at 16 and 18 months (total 14 doses administered):

- Median vaccine site induration, 8 mm (range 6-11) after dose 4; 7 mm (range 4-13) after dose 5.
- Fever was self-reported after 2 (14%) of 14 doses.
- “Sore arm” was reported after 2 (14%) of 14 doses.

Immunogenicity:

- Lymphocyte proliferation assay:
  - SRL 172 sonicate: 6 of 7 subjects had stimulation index >2
  - *M. avium* sensitin: No significant change in stimulation indices
- Antibody to *M. tuberculosis* lipoarabinnomannan (MTB LAM): 4 of 10 subjects had >2x increase

5.5.2 SRL 172, Study 002

Study overview:

- Subjects: 12 HIV-infected adults, CD4 ≥300 in US; all BCG-negative
- HIV-related characteristics: 3 on ART (1 or 2 drugs) at baseline, 5 on ART by end of study
- Dose schedule: 0, 2, 4 mos (12 subjects); 25 and 26 months (8 subjects)

Vaccine site reactions: Assessed at 2d, 14 d, 2 mos after each dose.

- Induration: maximum at 2 days, median 6 mm

Safety:

- Temperature was recorded daily at home for 14 days after each dose. All were normal,
- No systemic side effects were reported after any dose.
- CD4: Mean change from baseline to post-dose 3 was +28 (range: -137 to +137)
- HIV viral load: Mean $\log_{10}$ change from baseline to post-dose 3 was + 0.4 (range: -0.3 to +1.5)

Immunogenicity:

- Lymphocyte proliferation:
  - SRL 172: Four subjects had stimulation index >2 after dose 3 (baseline not available)
  - *M. avium* sonicate: No increases in response from baseline to post-dose 3.
- Antibody to MTB LAM: No change in antibody titer

Four patients were lost to follow-up. Among the 8 subjects who received 2 additional doses at 25 and 26 months:

- Erythema with or without induration: 4 (50%) of 8 patients (diameter not available).
- No systemic symptoms were reported.
- Stimulation indices to SRL 172 and *M. avium* were generally higher in vaccine recipients than in 7 unimmunized HIV-positive controls.

5.5.3 SRL 172, Study 003

Study overview:

- Subjects: 35 HIV-infected children, ages 6 mo to 13 yr

- Subject characteristics: CD4 ≥300, age 1-8; ART encouraged (data not available)
- Treatments: 23, SRL 172; 12, intradermal hepatitis B (control)
- Dose schedule: 0, 2, 4 mos

Vaccine site reactions:

- median induration, 5 mm at 2 days; 3 mm at 14 days; 0 mm at 2 mos; 2 subjects had 4-5 mm induration at end of study (1 at dose one site, 1 at dose two site)
- crusting: present after 2 (3%) of 68 doses
- "sore arm" reported after 19 (28%) of 68 doses

Safety:

- Fever: recorded after 9 (13%) of 68 SRL 172 doses vs 3 (9%) of 35 HB doses
- CD4: median change SRL 172 = -99, HB = + 89 [p=0.50]
- Viral load: median change = - 0.1 $\log_{10}$ in both groups

Immunogenicity:

- 1 SRL 172 recipient had 2x increase in Ab to LAM; no subjects had increased lymphocyte proliferation response to LAM

5.5.4 SRL 172, Study 004

Study overview:

- Subjects: 44 HIV-infected adults in Zambia, CD4; 22 BCG positive, 22 BCG negative; 31 male, 13 female; ages 21 to 51 yr.
- HIV characteristics: CD4 ≥200; none on ART
- Study design: open label
- Treatment, N, BCG status, and Dose schedule
  - SRL 172: 11 BCG-pos, 11 BCG-neg; 0, 2, 4, 12, 14 mo (5 doses)
  - borate buffered saline (control): 11 BCG-pos, 11 BCG-neg; 12, 14 mo only (2 doses)

Vaccine site reactions:

- induration
  - BCG pos: range over doses one to four: 11-14 mm at 2d, 0-3 mm at 14d;
    after dose five: median 5 mm at 2d, 3 mm at 14d
  - BCG neg: range over doses one to four: 8-11 mm at 2d, 0-3 mm at 14d;
    after dose five: median 6 mm at 2d, 3 mm at 14d
- drainage after 3 (3%) of 110 doses
- sore arm after 4 (4%) of 110 doses,

Safety:

- Temperature (measured daily for 15 days by subjects using digital thermometer): not fever noted
- Other symptoms: headache after 3 (3%) of 110 doses; malaise after 1 (0.9%) of 110 doses
- Viral load:
  - BCG neg: pre-dose 4 to post-dose 5: no significant differences compared to controls
  - BCG pos: baseline to post-dose 3, 0.5 log decrease (p=0.007);
    pre-dose 4 to post-dose 5: no significant differences compared to controls

Immunogenicity:

Table 5-3. Change in median lymphocyte stimulation index to SRL 172 sonicate, pre-dose 4 to post-dose 5

	SRL 172	Control	p-value
BCG negative	incr: 2.3 -> 6.0	decr: 3.7 to 2.6	<0.05
BCG positive	incr: 4.3 -> 8.8	unch: 1.9 to 1.9	<0.001

5.5.5 SRL 172, Study 005

Study overview:

- Subjects: 10 healthy HIV-negative adults, 39 HIV-infected adults with CD4≥200; all BCG-positive, in Finland
- Treatments:
  - SRL 172 at 0, 2, 4, 6, 12 months
  - Control vaccine (CV), hepatitis B vaccine at 0, 2, 12 mo; borate-buffered NaCl at 4, 6 mo (all intradermal)
- The 39 HIV-positive subjects were randomized between the two different treatments. Subject characteristics and outcomes are shown in Table 5-4.
- The 10 HIV-negative subjects all received SRL 172 at 0, 2, 4, 6, and 12 months. The key observations in these subjects were:
  - The five-dose schedule of SRL 172 was safe, well-tolerated and immunogenic.
  - Post-dose 5, LPA both to MTB whole cell lysate and to SRL 172 sonicate both were significantly greater than baseline ($p = 0.02$ and 0.008 respectively).
  - Post-dose 3, IFNg to SRL 172 sonicate was greater than baseline ($p = 0.06$).
- Discontinuations. Among 29 subjects who received SRL-172 no subject withdrew before dose 3. Two subjects withdrew after dose 3: subject P29 from 30 mm erythema at the injection site, subject P38 because of 25 mm erythema and drainage lasting 5 weeks (this subject had a chest x-ray consistent with prior TB). Three subjects withdrew after dose 4: N1 for arthralgias, P11 for musculoskeletal discomfort and P27 for a sterile abscess.

Ford von Reyn 5/31/15 10:25 AM
Deleted: Table 5-4

Table 5-4. SRL 172, Study 005: Characteristics, Safety, and Immunogenicity of HIV-positive Subjects by Treatment Group

Characteristics	SRL 172	Control Vaccine (CV)[a]
N	19[b]	20
Male (N, %)	17 (89%)	18 (80%)
Age (median)	40 yr	41 yr
HIV status	HIV-positive	HIV-positive
Combination anti-retroviral therapy	17	10
CD4 (median)	559 /mm^3	631 /mm^3
**Safety**		
ISR: induration 2d medians (range)	4-7 mm (0-30)	0-10
ISR: erythema 2d means (range)	10-17 mm (5-26 mm)	0-3 mm (0-10)
ISR: skin breakdown	11 – 37% post each dose	10 – 30% post each dose
ISR: drainage	5 – 11% post each dose	0 – 5% post each dose
ISR: sterile abscess	1 (5%)	0
ISR: "sore arm"	16 – 37% post each dose	10 – 30% post each dose
Fever	5% post each dose	5% post each dose
Adenopathy	5% post each dose	5% post each dose
Malaise	5 – 11% post each dose	5 – 11% post each dose
CD4 count (2 mo after dose 5)	no change from baseline<br>no significant diff c/w CV	no change from baseline
HIV viral load (2 mo after dose 5)	no change from baseline<br>no significant diff c/w CV	no change from baseline
**Serious adverse events**	0	0
**Discontinuations** [b]	5	0
After 3 doses	2	
After 4 doses	3	
**Immunogenicity**		
LPA to SRL 172 sonicate	increased c/w CV post dose 3, dose 5 and 1 year	
LPA to SRL 172 sonicate	post dose 5: median cpm 12,560 vs 22,547 in HIV-neg; p=0.17	
LPA to MTB sonicate	increased c/w CV post dose 3	
IFN-γ to SRL 172 sonicate	increased c/w CV post dose 3, dose 5 and 1 year	
IFN-γ to MTB sonicate	no significant difference c/w CV	

a. CV = intradermal hepatitis B vaccine at 0, 2, and 12 mo; borate buffered saline at 4 and 6 mo.
b. Five subjects withdrew due to adverse events: one each with (a) injection site sterile abscess post dose 4; patient had apical scarring consistent with prior TB; (b) injection site induration and drainage post dose 3; (c) injection site prolonged drainage post dose 3; and (d) musculoskeletal pain, temporally related to immunization after dose 4 and (e) arthralgias after dose 4..

5.5.6 SRL 172, Study 006 (DarDar Trial)

The DarDar Trial of SRL 172 was a 7-year Phase III, randomized, controlled, GCP-compliant trial conducted in Dar es Salaam, Tanzania, initiated in 2001, and sponsored by the National Institutes of Health (von Reyn, Principal Investigator).

Study overview (Table 5-5):

- Subjects: HIV infected adults (age ≥18) with prior BCG (by scar) and CD4 ≥200
- N: 2013; randomized (1:1) to SRL 172 (V) or placebo (P; borate-buffered saline)
- Dose schedule: 0, 2, 4, 6, and 12 mo (5 doses)
- Follow-up: Seen every 3 months; median 3.3 years.
- Endpoints – all reviewed by a blinded 3-person expert panel using pre-defined criteria.

Ford von Reyn 5/31/15 10:25 AM
Deleted: Table 5-5

- Primary endpoint: disseminated tuberculosis with positive blood culture
- Secondary endpoint: definite tuberculosis (all culture-positive tuberculosis)

Results. SRL 172 was safe, well-tolerated, and induced T cell responses against the vaccine antigen and antibody to lipoarabinomannan. The trial was stopped at 7 years when the Data Safety Board determined that the vaccine was effective based on a significant reduction in all culture positive tuberculosis and a trend in the reduction of disseminated tuberculosis.

Table 5-5. SRL 172, Study 006 (DarDar Trial): Subject Characteristics and Outcomes by Treatment Group

Characteristics	SRL 172	Placebo	P value
N	1006	1007	
Female (%)	774 (77%)	766 (76%)	NS
Age (median)	33 yr	33 yr	NS
CD4 (median)	428 /mm^3	404 /mm^3	NS
History of prior TB	9%	8%	NS
PPD ≥5 mm	31%	32%	NS
Current ART	3%	3%	NS
**Safety**			
ISR[1,2] induration d+7	6.2 mm (0 – 30)	0.1	
d+28	4.4 mm (0 – 30)	0.1	
ISR[2]: skin breakdown d+7	36 – 58%	1-3%	
d+28	17 – 23%	0-1 %	
ISR[2]: drainage d+7	22 – 49%	0-1 %	
d+28	11 – 20%	0%	
ISR: sterile abscess[3]	3 (0.3%)	0	
Fever (T ≥38°C)	1%	1%	NS
Feverish	7 – 14%	4 – 13%	NS
Headache	5 – 17%	4 – 13%	NS
Malaise	13 – 23%	11 – 18%	NS
CD4 count change[2]	-57	-58	0.76
Log $_{10}$ HIV viral load change[2]	+ 0. 08	+ 0.42	0.01
**Serious adverse events[4]**	209	232	0.24
**Discontinuations[5]**	46	31	0.08
**By MD**	11	1	
**By subject**	35	30	
**Immunogenicity[6]**	**Baseline to post-dose 5**	**SRL 172 vs Placebo at post-dose 5**	
LPA to SRL 172 sonicate	Increased	V increased	
LPA to ESAT, Ag85, or MTB whole cell lysate	NSD	NSD	
IFN-γ to SRL 172 sonicate	NSD (trend only)	V increased	
IFN-γ to ESAT, Ag85, or MTB whole cell lysate	NSD	NSD	
Antibody to LAM	Increased	V increased	
**Vaccine Efficacy[7,8]**			
Disseminated TB	7	13	HR 0.52; p = 0.16
Definite TB	33	52	HR 0.61; p = 0.027
Probable TB	48	40	HR 1.17; p = 0.46

NS, P value not significant; NSD, no significant difference.
1. ISR data are median (range) for all doses at day +28 post-dosing unless otherwise indicated. Maximum reactions typically after dose 3.
2. Data from Substudy A (162 subjects). CD4 and viral load change is from baseline to 2 mos post-dose 5
3. Injection site sterile abscesses occurred after the first dose in one patient and after the third dose in two patients (both with a history of prior TB); all abscesses drained spontaneously and resolved with routine wound care and oral antibiotic therapy.
4. None considered related to immunization.
5. Immunization was discontinued by the MD investigator in 12 (1.2%) subjects because of adverse reactions considered possibly or probably vaccine-related: 3 vaccine site abscesses five other local reactions and four generalized rashes. An additional 65 subjects (35 SRL-172, 30 placebo) withdrew themselves from the trial

before completing the trial citing a switch to alternative medicine, perceived vaccine side effects or inconvenience. All reactions were considered mild or moderate and resolved after discontinuation of immunization.

6. Immunogenicity assay results compare (a) baseline to post-dose 5 among SRL 172 recipients; (b) SRL 172 vs Placebo, post-dose 5. Only significant effects noted, defined as $p \leq 0.05$. NSD, no significant difference
7. Vaccine efficacy endpoints were prospectively defined; data were reviewed and outcomes assigned by blinded expert panel.
8. Disseminated TB, defined as a positive blood culture (Primary Endpoint). Definite TB, stringent laboratory-defined criteria (Secondary Endpoint). Probable TB, lesser laboratory findings or only clinical findings.

6. OBJECTIVES

6.1 Primary:

To evaluate the safety and tolerability of multiple doses of DAR-901, at different dose levels, administered to healthy HIV-negative and –positive adults by intradermal injection.

6.2 Secondary:

To characterize the immunogenicity of multiple doses of DAR-901 at different dose levels, with emphasis on the cytokine responses to DAR-901 soniciate (i.e., IFN-γ secretion and Elispot).

7. INVESTIGATIONAL PLAN

7.1 Overall Study Design

Overall study design is presented in Section 2.4, including

- Dose Groups (Table 2-1)
- Enrollment Process (Section 2.4.1)
- Study Structure (Section 2.4.2)
- Potential protocol adjustments (Section 2.4.4)
- Risk Management (Section 2.4.2)

Ford von Reyn 5/31/15 10:25 AM
Deleted: Table 2-1

7.2 Rationale for Treatment Regimens

7.2.1 Dose Levels

The proposed dose levels (0.1, 0.3, 1 mg per dose) are based on

- prior clinical experience with SRL172, a killed whole-cell vaccine prepared from the same genotypic strain grown on agar, and
- mouse immunogenicity studies conducted with DAR-901

Clinical studies with SRL172. All clinical studies by Dartmouth investigators were conducted with an adult intradermal dose of 1 mg in a volume of 0.1mL (dose in children < 5 = 0.05 mL). This dose was established in initial studies by British investigators using vaccine prepared by SR Pharma (London). In the DarDar Trial of HIV-infected patients this dose was found to be safe, immunogenic, and effective in the prevention of tuberculosis (see Section 5.5.6). This will be the maximal dose of DAR-901 in the present Phase I study.

Mouse immunogenicity studies with DAR-901.

7.2.2 Dose Number and Dosing Interval

The dose number and dosing interval are based on

- prior experience with SRL172, a killed whole-cell vaccine prepared from the same genotypic strain grown on agar, and
- published experience with other inactivated, whole-cell vaccines

Dose number. Inactivated whole cell vaccines are typically administered in a 2- or 3-dose schedule. Studies by Dartmouth investigators with SRL172 have shown safety and immunogenicity with both a 3-dose (Study 005, Table 5-4) and 5-dose schedule (Study 006, Table 5-5). A 3 dose schedule will be employed in the present study and immune assays will also be conducted after 2-doses.

Dosing interval. Prior studies by Dartmouth investigators have used 2 month dosing intervals for 3 doses or the first 3 doses (Studies 002-006, Table 5-2). Safety and immunogenicity have been demonstrated with a 3-dose schedule at 0, 2 and 4 mos (Study 005, Table 5-4)

Ford von Reyn 5/31/15 10:25 AM
Deleted: Table 5-4

Ford von Reyn 5/31/15 10:25 AM
Deleted: Table 5-5

Ford von Reyn 5/31/15 10:25 AM
Deleted: Table 5-2

Ford von Reyn 5/31/15 10:25 AM
Deleted: Table 5-4

7.3 Rationale for Study Design

The study design – randomized, placebo-controlled, double-blind – is consistent with both the study objectives and current principles for the evaluation of multiple dose courses of investigational treatments. In particular, double-blinding avoids bias by the Investigator and subjects in assessing the subjective aspects of the study, particularly adverse events. The first subject is dosed open-label consistent with risk management for a phase 1 study of an immunogenic vaccine.

The study includes both HIV-negative and –positive adults because the target populations for the vaccine include both populations, but their immune response to the vaccine may be qualitatively and/or quantitatively different.

7.4 Maximum Exposures and Maximum Number of Subjects

The maximum exposures to DAR-901, the investigational agent, will be:

- Dose level: 1 mg per dose (see Table 2-1)
- Number of doses: 3 doses over 4 months (120 days)
- Number of subjects: 36 HIV-negative and 16 HIV-positive subjects in planned cohorts

Ford von Reyn 5/31/15 10:25 AM
Deleted: Table 2-1

7.5 Definitions Applicable to Managing the Study

Treatment-emergent is defined as onset after active engagement in the trial; that is, after the administration of the first injection of study treatment. It is anticipated that treatment-related events may be observed after each injection of study treatment.

7.5.1 Definition of a Dose-Limiting Toxicity (DLT) Event

A DLT *systemic clinical event* is defined as a treatment-emergent systemic clinical event that meets ***all*** of the following criteria:

- is assessed by the investigator as related or possibly related to study drug (see Section 12.3.3);
- is of severity (see Section 12.3.4) Grade 2 or higher;
- is of duration >48 hours.

A DLT *injection site reaction* (ISR) is defined as an event that meets ***both*** of the following criteria:

- occurs at the injection site;
- is of severity Grade 3 or higher.

A DLT *laboratory event* is defined as a confirmed, treatment-emergent laboratory finding that meets ***both*** of the following criteria:

- is ***not*** considered consistent with a concurrent clinical event that is assessed as not related or unlikely related to study drug (e.g., an accidental injury, new ART); ***and***
- based on pre-specified toxicity criteria (see Section 12.5.5), represents an increase of two Grades or more compared to pre-treatment baseline value.

A subject who experiences a DLT event will not receive further doses of study drug. Unblinding may be performed to determine if a DLT event has occurred in a subject receiving DAR-901.

7.5.2 Definition of a Completed Patient

A **subject** is considered complete when s/he meets ***either*** of the following criteria:

- received all scheduled doses of DAR-901 and completes the study visit at Dose #3 +56 days; ***or***
- received at least one dose of DAR-901 ***and*** had treatment discontinued due to a DLT event.

7.5.3 Definition of a Completed Dose Group

A dose group is complete when ***either*** of the following criteria are met:

- At least 80% of the DAR-901 subjects in the dose-group are complete (that is, 8 of 10 or 5 of 6 subjects, as appropriate; to be determined by an unblinded statistician); ***or***
- the dose-group is terminated under the procedures detailed in Section 7.7.

7.6 Procedures for Managing the Study

7.6.1 Discontinuing Study Treatment in Individual Patients

Study treatment may be discontinued in an individual patient for ***any*** of the following reasons:

- The subject withdraws from study participation by their own decision ("withdrawal of consent"); this may happen at any time and for any reason without prejudice for their continued care.
- The subject has a DLT event as defined in Section 7.5.1.
- The Investigator determines, based on their judgment, that discontinuation of study treatment is in the subject's best interest, e.g., due to an adverse event, noncompliance, or any reason, whether or not related to study drug or study procedures.

If study treatment is discontinued prematurely, the reasons will be recorded and, if possible, the EOS visit will be performed as specified (see Section 11). If a subject cannot be seen, attempts will be made to contact the subject by telephone.

7.6.2 Replacement of Subjects

A subject who does not meet criteria for "complete" (e.g., withdrawal of consent) may be replaced to assure the requirements for completing a cohort are met. A replacement subject will be identified by a distinctive subject number and will receive the same treatment as the subject being replaced.

7.7 Dose Review Committee (DRC)

As part of the comprehensive risk management program, the Sponsor will establish a Dose Review Committee (DRC) comprising two independent physicians with clinical trials experience (preferably including early phase and/or vaccine studies) and a representative of the Sponsor who has prior experience with SRL-172. One of the independent members of the DRC may also serve as Medical Monitor for consultation by the Investigator.

The DRC will serve the following functions:

- Perform the reviews specified in Section 2.4.1, including dose escalation, dose selection, and progression to HIV-positive subjects as specified in a Charter. Majority approval by the DRC is required to proceed.
- Be notified of and review promptly any Serious Adverse Events. If the DRC considers that the SAE may be treatment-related, they may request to be unblinded in order to proceed to a recommendation regarding the ongoing conduct of the Study.
- Be notified and review promptly if two (2) or more subjects in a dose group experience a DLT event. The DRC may request to be unblinded if they judge that necessary in order to provide the Sponsor recommendations regarding the ongoing conduct of the Study.
- Recommendations regarding the conduct of the study may include, but are not limited to, increasing safety monitoring procedures or tests; de-escalating to an intermediate dose level; terminating study treatment in one or more ongoing cohorts.

7.8 Discontinuation of the Study

The Sponsor may terminate the study at any time for any reason. Subjects would still be followed for safety. In the event the study is terminated, the IECs and appropriate regulatory authorities will be notified of the decision.

8. SUBJECT SELECTION

8.1 Source of Subjects and Recruitment Methods

Following appropriate Human Studies review and approval (see Section 15), the Investigator may initiate and manage subject recruitment. To reach an economically and socially diverse population, the study may be announced in newspapers and on relevant Internet websites.

Ford von Reyn 5/31/15 10:25 AM
Deleted: 15

8.2 Subject Disclosures and Restrictions during the Conduct of the Study

This is a Phase 1 study of healthy subjects and subjects with HIV infection. In the interest of their safety and to facilitate accurate assessment of the data, the subjects will agree to the disclosures and restrictions detailed in Table 8-1 for the duration of their participation in the study, i.e., screening visit to End-of-Study (EOS) visit.

Ford von Reyn 5/31/15 10:25 AM
Deleted: Table 8-1

Table 8-1. Subject disclosures and restrictions during the conduct of the study

Item / Activity	Action	Comment
Prescription medication	Disclosure	Prescription medications in use at the time of screening will be reviewed in detail. Medications subsequently prescribed by physicians other than the Investigator will be disclosed promptly.
Over-the-counter medication	Disclosure	Over-the-counter medications in use at the time of screening will be reviewed in detail. Over-the-counter medications subsequently initiated by the subject will be disclosed promptly.
Herbal products[a]	Prohibited	Patients will be instructed about the range of products containing St. John's Wart and other herbals.
High-dose Vitamins[b]	Prohibited	Vitamins and minerals in doses substantially exceeding recommended daily requirements.
Blood donation	Prohibited	—

a. Patients will be instructed about the range of products containing St. John's Wart, other herbals, caffeine or xanthines (including chocolate).
b. Vitamins and minerals in doses substantially exceeding recommended daily requirements.

8.3 Definitions

8.3.1 Non-childbearing potential

Non-childbearing potential is defined as meets ***one*** of the following two criteria:

- documented hysterectomy or bilateral oophorectomy.
- age ≥50 years, no menses for at least 3 years, **and** post-menopausal FSH level;

8.3.2 Effective birth control (contraception) methods

Effective birth control (contraception) methods means strict abstinence or use of ***two*** of the following methods: hormonal contraceptive (oral, injectable, implanted [e.g., Implanon™], or intravaginal ring), condom, diaphragm, spermicide, or an intra-uterine device.

8.4 Subject Selection Criteria

Subject selection criteria are detailed in Section 2.4, including

- Inclusion and Exclusion Criteria – HIV-negative Subjects (Sections 2.5.1 and 2.5.2, respectively)
- Additional Inclusion / Exclusion Criteria for HIV-positive Subjects (Section 2.5.3 and 2.5.4)

8.4.1 Observed Variances

Subjects meeting inclusion and exclusion criteria at screening may be scheduled for enrollment. If on Day 1 prior to the first dose, clinical variances are noted compared to screening enrollment may proceed with the approval of the Medical Monitor.

9. STUDY TREATMENTS

9.1 Study Treatments to be Administered

Table 9-1. Study treatments

Role in Study	Identity of treatment	Comments
Investigational	DAR-901	Administered by ID injection
Active Comparator	Tice BCG	Organon, Teknika, 0.1 mL intradermal, diluted to 1-8 x 10^6 CFU
Placebo Control	Sterile Saline for Injection	Administered by the same route and in the same dose volume.

9.2 Identity of the Investigational Product

Table 9-2. Physical and Chemical Properties of Active Ingredient (Drug Substance)

**Name**	DAR-901
**Vaccine Class**	whole-cell, heat-killed organisms
**Appearance**	Slightly turbid yellow suspension

Table 9-3. Formulation of DAR-901 for Injection (Drug Product)

**Name**	DAR-901 for Injection
**Active ingredient**	DAR-901
**Excipients**	Borate-buffered 0.9% NaCl
**How supplied**	Sterile 2 mL vial containing 0.35 ± 0.05 mL of 10 mg/mL DAR-901
**Storage**	2-8°C
**Preparation and handling**	Preparation varies by dose level; see Section 9.3 for details.
**Administration**	The dose is administered as a single intradermal (ID) injection in the deltoid using a fresh sterile needle (approximately 26g).

Table 9-4 Formulation of Tice BCG for Injection (Positive Control)

**Name**	Tice BCG
**Active ingredient**	M. bovis, BCG
**How supplied**	Freeze dried preparation in vial containing 1-8 x 10^8 CFU BCG
**Storage**	2-8°C
**Preparation and handling**	Reconstituted with sterile water to achieve concentration of 1-8 x 10^6 CFU BCG/0.1mL
**Administration**	The dose is administered as a single intradermal (ID) injection in the deltoid using a fresh sterile needle (approximately 26g).

9.3 Preparation and Handling of DAR-901 for Injection by Dose Level

9.3.1 0.1 mg DAR-901 for Injection

- Gently agitate the vial (0.3-0.4 mL at 10mg/mL) to assure an even suspension.
- Withdraw 0.22 mL from the vial.
- Add 0.22 mL to a 2 mL vial of Sterile Saline for Injection, USP.
- Gently agitate the vial to assure an even suspension.
- Withdraw 0.1 mL from the vial for administration.

9.3.2 0.3 mg DAR-901 for Injection

- Add 0.65 mL of Sterile Saline for Injection, USP to the vial.
- Gently agitate the vial to assure an even suspension.

- Withdraw 0.1 mL from the vial for administration.

9.3.3 1 mg DAR-901 for Injection

- Gently agitate the vial to assure an even suspension
- Withdraw 0.1 mL from the vial for administration.

Detailed dose-preparation instructions and flow-sheets will be provided in the Pharmacy Manual.

9.4 Reference and Blinding Therapy

Tice BCG is used as the active comparator. Sterile Saline for Injection is used as the placebo control and for blinding (i.e., the first and second injections in BCG subjects).

9.5 Administration of Study Treatments

Each dose of DAR-901, BCG or placebo will be administered as a single intradermal (ID) injection. The recommended site for injection is the upper deltoid region of the arm, with sequential doses administered in the opposite arm. In the event this recommendation cannot be accommodated due to injury or other issue, the same arm may be used successively, placing the injection at least 5 cm apart.

9.5.1 Variances in Dose Administration Schedule

The protocol permits variance in dose administration of plus or minus seven (7) days from the nominal scheduled timepoint. Variances that would exceed plus or minus seven (7) days should be discussed with the Medical Monitor.

9.6 Method of Assigning Patients to Treatment Groups

The first subject in dose group will be assigned active treatment (DAR-901)(see Section 2.4.1). For subsequent subjects within the dose group treatment will be assigned by computer-generated randomization for A1-A3 and open label for A4, B1 and B2. Suitable randomization procedures will be established by the CRO data management and clinical services performing the protocol and approved by the Sponsor. The research pharmacist will be provided a list of treatment assignments.

9.7 Selection of Dose Levels in the Study

See Section 7.2.1.

9.8 Selection of Dose Number and Interval

See Section 7.2.2.

9.9 Blinding

Subjects randomized to placebo regimen will receive an injection of Sterile Saline for Injection at the same dose volume as active treatment. The pharmacist is responsible for maintaining the blind, that is, assuring that treatment allocation is not revealed to other study staff or the patients.

9.9.1 Procedures for Unblinding Individual Patients during the Study

There are no specific treatments for the effects of DAR-901; the Investigator should manage patients symptomatically based on any changes observed. Consequently, it is not expected that the treatment allocation for a particular subject will need to be revealed (i.e., unblinded).

If the Investigator needs to unblind the treatment assignment for a particular subject, prior approval by the Medical Monitor should be obtained and the following information entered into the medical record:

- date and time of the last injection,
- reasons study drug was discontinued,
- name of the Medical Monitor who approved unblinding,
- reasons the subject's treatment allocation was unblinded.

In the event of a true medical emergency in which the Investigator judges that the subject cannot be managed safely without unblinding, the Investigator may obtain the treatment allocation directly from the

pharmacist at the site. All steps above will be followed, including contacting the Medical Monitor as soon as possible and not more than 24 hours afterwards.

9.10 Prior and Concomitant Therapy

See Section 8.2. Treatments prohibited prior to enrollment are prohibited for the duration of the study.

9.11 Treatment Compliance

All doses will be administered by study personnel.

9.12 Accountability of Investigational Drug Supplies

The Investigator at each study site will identify trained and experienced personnel to handle the study drug in accordance with the protocol and appropriate GCP and GMP principles. This includes:

- storing the drug in a secure, limited access facility and under the appropriate conditions;
- dispensing and administering study drug only in accordance with the protocol;
- maintaining drug accountability records;
- at the completion of the study, returning or destroying unused study drug in compliance with the written instructions of the Sponsor.

Detailed procedures will be provided in a separate Pharmacy Manual for this study.

10. STUDY EVALUATIONS

Detailed schedules of evaluations are shown in Table 2-2 and Table 2-3.

Ford von Reyn 5/31/15 10:25 AM
Deleted: Table 2-2
Ford von Reyn 5/31/15 10:25 AM
Deleted: Table 2-3

10.1 Medical History and Physical Exam

The Investigator will perform a comprehensive history and physical examination at the Screening visit; any new findings observed at subsequent scheduled and unscheduled examinations will be recorded.

10.2 Vital Signs

Vital signs include temperature, heart rate (HR), respiratory rate (RR) and blood pressure (BP). Where feasible, vital signs should be measured before blood is drawn and after the patient has been sitting comfortably for ~5 min with the BP cuff in place (preferably on the non-dominant arm). BP and HR measurements may be done manually or by automated recorder. Temperature will be obtained using an electronic (rapid reading) device. Respiratory rate will be determined by observation for at least 30 sec.

10.3 Laboratory Studies

The laboratory tests indicated below will be performed by a CLIA-approved laboratory proposed by the Investigator at each site and approved by the Sponsor. Details of procedures for collecting, processing, storing and shipping the blood samples will be provided in a separate Laboratory Manual.

The Investigator may order additional local laboratory tests consistent with their routine standard of care.

10.3.1 Safety Laboratory Tests

Table 10-1. Hematology Panel

Hemoglobin	White blood cell differential (if WBC abnormal)
Hematocrit	- Neutrophils
Total red blood cell count	- Lymphocytes
Mean cell hemoglobin	- Monocytes
Mean cell hemoglobin concentration	- Eosinophils
Mean cell volume	- Basophils
White blood cell count	
Platelet count	

Table 10-2: Clinical Chemistry Panel

Glucose	Alanine aminotransferase (ALT)	Total bilirubin
Urea	Aspartate aminotransferase (AST)	Total protein
Creatinine	Alkaline phosphatase (AP)	Albumin
	Lactate dehydrogenase (LDH)	
	Creatine phosphokinase (CK)	

Table 10-3. Coagulation Panel

International normalized ratio (INR)

Table 10-4. Urinalysis

Specific gravity, pH, ketones, glucose, protein, blood (commercial dipstick may be used); microscopic examination, including quantitation of WBC and RBC;

10.3.2 HIV Monitoring Tests

Quantitative HIV viral load (sensitivity, 20 copies/mL; Ampliprep Taqman®, Roche or comparable commercial assay approved by the Sponsor); CD4 lymphocyte count.

10.3.3 Screening Serologic Tests

The following serology tests will be performed at screening: Hepatitis B surface antigen (HBsAg), antibody to hepatitis C virus, antibody to HIV-1 and HIV-2.

Examples of suitable HIV antibiody tests include: Vitros Anti-HIV 1 + 2 test, Ortho Clinical Diagnostics, Rochester, NY; or Clearview HIV1/2 STAT-PAK (Alere North America, Inc; Orlando, FL. Additional tests may be approved at the discretion of the Sponsor.

10.3.4 Pregnancy Tests

For all females, pregnancy (β-hCG) testing will be conducted as scheduled. A negative pregnancy test result must be reported within 24 hours prior to the first dose of study drug.

10.4 Reporting of Safety Laboratory Tests

The results of Safety Laboratory Tests will be returned to the Investigator as quickly as possible, typically within 48 hours. Reference ranges (lower limit of normal, upper limit of normal; by sex and age, if appropriate) will be provided for all laboratory analytes.

Procedures for the investigator assessment of laboratory results are detailed in Section 12.1.5.

Procedures for the analysis of laboratory data are described in Section 14.4.

10.4.1 Repeating Abnormal Laboratory Tests

Laboratory tests showing abnormal or exclusionary values at screening may be repeated no more than once. After dosing, abnormal laboratory tests assessed as “clinically significant” values may be repeated as often as deemed clinically necessary by the Investigator until the test values return to clinically acceptable limits or until an explanation other than drug effect is given.

10.5 Immunogenicity Assessments

10.5.1 Preparation and storage of Peripheral Blood Mononuclear Cells (PBMCs)

Whole blood will be collected as scheduled. PBMCs will be isolated using Ficoll density centrifugation within 2 hours of collection. Isolated cells may be used directly for immunogenicity assessments or stored frozen at -70C or lower.

Table 10-5. Immunogenicity assays

Assay [ref]	Purpose / Rationale	Antigens[1]	Estimated PBMC/Assay	Estimated blood vol
Multiplex (13 color) intracellular cytokine staining (ICS) assay for IFN-γ, TNF-α and other cytokines [9]	Sensitive assay for cytokine responses to major antigens; patterns define functional T cell response and phenotype	DAR-901 lysate<br>MTb whole cell lysate<br>Medium<br>SEB	4 x 10^6	10 mL
ELISA of 5-day cell culture assayed for IFN-γ [10,11][12]	Assay of cytokine response to major antigens<br><br>ELISA for IFN-γ used in DarDar trial.[b]	DAR-901 lysate<br>MTb whole cell lysate<br>Ag 85, ESAT<br>Medium<br>Phytohemagglutinin	3 x 10^6	5 mL
IFN-γ ELISpot [13]	Standard assay that allows scalable assessment of antigen specificity of responses	Medium<br>Phytohemagglutinin<br>DAR-901 lysate<br>MTb whole cell lysate<br>*and/or*<br>Panel of mycobacterial peptides and lipopeptides	2 x 10^6<br><br>up to 15 x 10^6 for panel of multiple specific antigens	5 mL
Mycobacterial growth inhibition assay (MGIA) [14,15,16]	Functional assay of PBMC capacity to inhibit growth of mycobacteria.[a]	N/A	2 x 10^6	5 mL

RNA analysis (transcriptional profiling)	Defines changes in expression of immune response related-genes		Pax-gene tube	3 mL
Antigen-specific Serum IgG responses by ELISA [10]	Highly sensitive, reproducible assay; assesses T-cell dependent humoral immunity	Lipoarabinomannan	N/A (serum only)	7 mL (for serum)

a. The mycobacterial growth inhibition assay (MGIA) is being evaluated as a potential "marker" of effective vaccination in clinical trials of new TB vaccines. Studies using BCG vaccine in human subjects show that PBMCs from vaccinated individuals result in growth inhibition of live BCG or *Mtb* as measured by the Becton Dickinson MGIT.

b. Among the placebo subjects in the DarDar trial, baseline responses in the IFN-γ cytokine release assay were associated with decreased risk of incident definite tuberculosis.

Note: Aeras will receive PBMCs from 20 mL of blood (PBMCs from each 10 mL into separate cryovial) and Dartmouth from 5 mL of blood. Pax gene tube will also be processed by Aeras

Estimated blood volume requirements. The full panel of cellular assays would require ~24 x 10^6 PBMCs. For healthy adults, ficoll density gradient centrifugation typically yields 1.3 x 10^6 PBMCs per mL of whole blood. Yields from HIV-infected subjects are more variable, depending on current anti-retroviral therapy and other clinical factors, but are usually 0.6 to 1.1 x 10^6 per mL. Thus, the proposed 40 mL would be expected to yield, for healthy volunteers, 52 x 10^6 PBMCs, and for HIV-positive subjects, 24 to 44 x 10^6. Any unused cells will be retained for up to two years for supplemental assays.

Comments. In assessing immunogenicity, primary emphasis will be given to two antigen-specific assays:

- IFN-γ stimulation by ELISpot in response to mycobacterial antigens
- IFN-γ production in response to DAR-901 lysate in PBMC culture supernatants

The 13-color ICS assay and the IFN-γ ELISpot assay will be conducted at Aeras' immunology laboratory using frozen PBMC. The reagents, equipment, and training of personnel have been standardized, and both assays are being qualified for use in vaccine trials. Conducting these assays in single laboratory reduces assay variability and is particularly critical for determining the correlation between the two assays.

Cytokine release by cultured PBMCs was the primary immunologic assessment used in the DarDar trial (see Section 5.5.6)[10, 11].

RNA transcriptional analysis is an exploratory assessment at this time. It offers the potential to assess changes in the expression of genes related to both innate and adaptive immunity and to identify the activation of specific intracellular pathways.

The mycobacterial growth inhibition assay (MGIA) is currently being developed by Aeras with the potential for qualification as a new method to directly assess the ability of PBMCs to inhibit mycobacterial growth. Assays from all 6 timepoints will be conducted at the same time on frozen PBMCs.

11. STUDY EVENTS

Detailed schedules of evaluations are shown Section 2.9 ; Table 2-4 gives an estimate of blood volumes required. The schedule is presented relative to the day and time of dosing. All Days are relative to day of first injection of study drug, designated Day 1; all times are relative to the most recent injection, designated 0 hr.

Ford von Reyn 5/31/15 10:25 AM
Deleted: Table 2-4

Monitoring for adverse events and concomitant medications will be performed on an ongoing basis from screening through End-of-Study visit.

11.1 Screening

The screening evaluation may be performed up to 28 days prior to dosing. The subject will sign an Informed Consent Form before any study-specific procedures are performed. The IRB approved Informed Consent Form (ICF) will be provided on electronic tablets which hold the source documents for the study.

The subjects will be able to review the informed consents on the tablet or they can receive a paper copy of the form to review. Signatures on the ICF will be made on a printed copy and then uploaded to the electronic tablets. The subjects will receive a printed version of the signed informed consent for their records. A PDF of the informed consent will be added to the subject's Electronic Health Record. Data from each subject that signs an informed consent form will be maintained within the electronic Source system (Clinical Ink).

11.2 End-of-Study Visits For Subjects who Terminate Prematurely

Subjects who terminate prematurely, for any reason, should have a final safety visit completed at approximately 30 days after the last dose received, or if that timepoint is already passed, as soon as possible. This final visit should include the procedures scheduled for Day +28 after Dose #3. If they cannot complete a visit, safety follow-up should be conducted by phone.

12. SAFETY EVALUATIONS

Clinical trials sponsored by Dartmouth College will be conducted in accordance with Good Clinical Practices for collecting and reporting safety information. Safety and tolerability will be evaluated based on AEs, vital signs, physical exams, laboratory tests and other assessments.

12.1 Definitions

12.1.1 Adverse Event (AE)

An Adverse Event is any untoward medical occurrence temporally associated with the use of a medical product in a subject, ***whether or not*** the event is considered causally related to the medical product.[22] An AE can be a new occurrence or an existing process that increases significantly in intensity or frequency.

An AE in a clinical trial may be ***any*** of the following:

- Unfavorable and unintended *symptom reported by the subject* — subjects will be encouraged to report treatment-emergent AEs spontaneously; general, non-directed questioning may also be used to elicit reports of AEs;
- Clinical *sign detected by the Investigator* — observations by other study personnel will be reported to the Investigator for evaluation;
- Is a treatment-emergent new or increased abnormal result from a *laboratory study* or other *diagnostic procedure.*

12.1.2 Pregnancy

Pregnancy is not an AE. Pregnancy testing will be performed as scheduled. If a female subject becomes pregnant during a study, the Medical Monitor must be notified in writing within five days. Follow-up information regarding the outcome of the pregnancy and any postnatal sequelae in the infant will be obtained.

12.1.3 Serious Adverse Event (SAE)

An AE is **serious** when the subject outcome is one or more of the following:

- Death.
- Life-threatening, meaning that the subject was at immediate risk of death from the event at the time that the event occurred. It does not include an event which hypothetically might have caused death if it occurred in a more severe form.
- Hospitalization, initial or prolonged, meaning that a hospital admission and/or prolongation of a hospital stay was required for the treatment of the AE, or occurred as a consequence of the event. It does not include a pre-planned elective hospital admission for treatment or diagnostic procedures, or, in general, a hospital admission of less than 24 hours duration.
- Disability or incapacity that is persistent or significant.
- Congenital anomaly or birth defect.
- Important medical event that, although not immediately life-threatening, requires intervention in order to prevent one of the other serious outcomes listed above. Examples of such events are allergic bronchospasm requiring intensive treatment in an emergency room or at home; blood dyscrasias or convulsions that do not result in hospitalization; or the development of drug dependency or drug abuse.

[22] A medical product may be a drug or a device being used either prior to or after regulatory approval. The medical product in this protocol will hereafter be referred to as study drug (synonym: investigational agent).

12.1.4 Suspected, Unexpected Serious Adverse Reaction (SUSAR)

A SUSAR is defined as an SAE that meets ***both*** the following criteria with respect to study drug:

- *Suspected* — is assessed as related or possibly related to study drug (see Section 12.3.3);
- *Unexpected* — compared to the study drug-related AEs described in Investigator's Brochure, the event meets ***any*** of the following criteria:
  - The event was not previously described;
  - The event is now characterized as more severe (see Section 12.3.4);
  - The event is now characterized more specifically (e.g., an event of "interstitial nephritis" in a subject receiving an agent previously described as associated with "acute renal failure").

In clinical trials involving ill patients, events considered related to the natural history of the disease under study or to lack of efficacy (that is, the event is considered more likely related to those factors than to other factors, including study drug) are not considered "unexpected". Lack of efficacy is recorded as specified elsewhere in the Protocol.

12.1.5 Investigator Assessment of Safety Laboratory Tests

The Investigator will review the results of all Safety Laboratory tests (see Section 10.3.1 and Section 10.3.2) and designate any results outside of the reference range as ***either*** of the following:

- Abnormal, not clinically significant (NCS)
- Abnormal, clinically significant (CS).

In making this judgment, the Investigator will consider all available information, including the patient's clinical condition, all available laboratory results (central and local), and the potential for false positive test results. In addition, laboratory studies that result in the actions specified in Section 12.1.1 will be classified as "abnormal, clinically significant".

Any result assessed as "abnormal, clinically significant" will be recorded as an AE *unless* it is consistent with one or more of the following:

- Process noted in the medical history.
- Ongoing adverse event already recorded;
- Expected course of the primary disease under study (if applicable);

12.2 Collecting and Recording Adverse Events

Procedures for the collection and recording of AEs are as follows:

- From obtaining informed consent through administration of the first dose of study drug (Day 1), there will be active surveillance to identify all AEs; events will be recorded in the medical history.
- Both at study visits and scheduled phone calls subjects will questioned using both a scripted checklist to elicit anticipated vaccine-related adverse events, as well as open-ended queries to elicit unanticipated events.
- From administration of the first and second doses of vaccine through the phone calls scheduled for day 28 post-dosing, and from the third dose of vaccine through the visit scheduled for day 28 post-dosing, there will be active surveillance to identify all AEs; events will be recorded in the ISR or AE portions of the CRF, as appropriate.
- At the visit for administration of Doses 2 and 3 and at the End-of-Study visit (Day 173-187), subjects will be asked about AEs with onset >28 days after the most recent dose and all events qualifying as SAEs will be recorded.
- After the EOS, surveillance will be passive (only events brought to the investigator's attention will be considered) and only events assessed as SUSARs will be recorded (see Section 12.4).

12.3 Characterizing Adverse Events

For each AE recorded the following characteristics will be noted.

12.3.1 Description of Event

The diagnosis or description will be as specific and complete as possible (i.e., “lower extremity edema”, rather than just “edema”). Whenever possible, signs and symptoms due to a common etiology will be reported as an integrated diagnosis; for example, cough, runny nose, sneezing, sore throat and head congestion would be reported as “upper respiratory infection”.

12.3.2 Date and Time of Onset

The date and time at which the event was first apparent. Table 12-1 summarizes the basis for reporting the date and time of onset for the different types of AEs described in Section 12.1.1.

Ford von Reyn 5/31/15 10:25 AM
Deleted: Table 12-1

Table 12-1. Reporting the Date and Time of Onset of AE for Different Types of Events

Type of Event	Examples	Source of Date and Time of Onset
Symptom	Headache, feverish, paresthesias	When first experienced by the patient
Sign (Finding)	Elevated BP, enlarged liver on physical exam	When first observed by the Investigator or other study staff
Laboratory / diagnostic result	Neutropenia, hyperglycemia, lesions on brain scan	When lab sample was obtained or diagnostic study performed

The time of onset of symptoms may be appreciably earlier than the date and time the Investigator becomes aware of the event. Some events may be apparent to the patient and Investigator independently, and information from each may contribute to the final report. For example, a patient may report the onset of a rash two days before being seen by a physician who makes a diagnosis of herpes zoster based on appearance and laboratory confirmation. In that case, there is a single AE, with the date of onset based on the date of the initial observation by the patient and a specific description (herpes zoster) based on the clinical exam and tests.

12.3.3 Relationship to Study Drug

This determination is based on the Investigator’s clinical judgment and the Medical Monitor’s clinical judgement regarding the likelihood that the study drug caused the AE and may include consideration of some or all of the following factors:

- Alternative possible causes of the AE, including the subject’s underlying disease or co-morbid conditions, other drugs, other host and environmental factors;
- Temporal sequence between the exposure to study drug and the AE;
- Whether the clinical or laboratory manifestations of the AE are consistent with known actions or toxicity of the study drug;
- Whether the AE resolved or improved with decreasing the dose or stopping the study drug (i.e., dechallenge); or recurred or worsened with re-exposure to the drug (i.e., rechallenge).

The relationship between the study drug and the AE will be described using one of the following categories:

- ***Related*** — the study drug is more likely the cause of the AE than other factors;
- ***Possibly related*** — there is a *reasonable* possibility that the study drug is the cause of the AE, including that the study drug and another factor(s) are equally likely as causes of the AE;
- ***Unlikely related*** — another factor is considered more likely the cause of the AE than the study drug;
- ***Not related*** — another factor is considered to be the cause of the AE.

Related or possibly related AEs may result during the use of the study drug as planned (per protocol), or from abuse, withdrawal or over-dosage of the agent.

12.3.4 Intensity (Severity)

The intensity (synonym: severity) of clinical AEs (i.e., symptoms reported by the patient and/or signs observed by the investigator) will, in general, be assessed by the Investigator using the five-level grading system (Table 12-2; adapted from CTCAE v4.02 [17]). The system reflects the duration of the event, its impact on the subject's activities, the level of medical intervention required, and, for events assessed as related or possibly related to study drug, the action taken with study drug. The Table below is intended to provide guidance; the investigator should use judgment in assigning an intensity grade to an event. In some instances a single characteristic may determine the grade; in other instances, the overall pattern may be considered more appropriate.

Ford von Reyn 5/31/15 10:25 AM
Deleted: Table 12-2

For this purpose, activities of daily living (ADL) are classified into two subsets:

- ***Instrumental ADL*** — e.g., preparing meals, shopping for groceries or clothes, using the telephone, managing money;
- ***Self-care ADL*** — e.g., bathing, dressing and undressing, feeding self, using the toilet, taking medications, and not being bedridden.

Note that the Sponsor provides specific guidelines for grading injection site reactions (see Section 12.5).

Table 12-2. Guidelines for grading the intensity (severity) of AE

**Event Characteristic**	**Event Grade** [a]			
	**1 (Mild)**	**2 (Moderate)**	**3 (Marked)**	**4 (Extreme)** [b]
**Duration of symptoms**	Transient, typically <48 hrs	Up to 2 weeks	>2 weeks, reversible	Symptoms / disabilities may be permanent
**Impact on ADL**	No limitations in ADL;	Some limitations in age-appropriate instrumental ADL	Some limitations in self-care ADL	Limitations in all activities; significant assistance required
**Medication intervention**	None or only OTC meds	OTC or prescription meds; provide relief	Prescription meds required; relief may be partial	Multiple meds required
**Interventions other than medication**	Minimal, local, or non-invasive	Minimal, local, or non-invasive	May be hospitalized <24 hr	Hospitalization >24 hr; surgery
**Typical action with study drug** [c]	No adjustment in study treatment regimen required	Study drug may or may not be continued	Study treatment may be discontinued	Study drug is discontinued

[a]. **Grade 5** is death (fatal) and is reserved for the particular AE that is assessed as the primary cause of death. Alternative terminology in use for Grade 3 includes "Severe"; for Grade 4 includes "Life-Threatening".
[b]. Typically "Life-Threatening," that is, imminent risk of death, urgent or significant intervention required.
[c]. Applicable only if event assessed as related or possibly related to study drug.

12.3.4.1 Relationship Between "Intensity" (Severity) and Seriousness in Characterizing Clinical AEs

Intensity (severity) and seriousness are distinct and independent items, with some interrelationship. By definition, clinical events assessed as SAEs meeting criteria for "death" would be Grade 5 and those meeting criteria for "hospitalization" or "life-threatening" would be Grade 4. Typically, events assessed as SAEs based on associated "significant disability" or being "medically important" would be Grade 3 or Grade 4. However, a clinical event may be assessed as Grade 3 and *not* qualify as an SAE; for example, a patient with a history of migraine headaches could have an episode that restricted them to bed for several hours but responded to the usual treatment, ran its usual course and had no sequelae.

12.3.5 Management of Study Drug

For each AE the Investigator will indicate which one of the following actions regarding the administration of study drug (study treatment) was taken because of that AE:

- ***Drug withdrawn (discontinued)*** — study drug was stopped permanently due to the AE;
- ***Drug interrupted*** — study drug regimen was modified temporarily, including one or more doses were not administered, but drug was not stopped permanently;
- ***Dose reduced (decreased)*** — study drug regimen was modified by subtraction, i.e., by decreasing the frequency, strength or amount;
- ***Dose Increased*** — study drug regimen was modified by addition, i.e., by increasing the frequency, strength or amount;
- ***Dose not changed (No action taken)*** — no change in the administration of study mediation.

12.3.6 Actions Taken for Management of AE

AEs will be followed and managed by the Investigator, including obtaining any supplemental studies needed to define the nature and/or cause of the event (e.g., laboratory tests, diagnostic procedures, consultation with other health care professionals).

For each AE the Investigator will categorize as follows the actions taken to manage the AE:

- ***Concomitant medication*** — one or more medications (prescription or over-the-counter) were started or increased in dose; non-medication actions may *also* have been ordered.
- ***Other action*** — *only* non-medication action(s) were ordered as management of the AE (e.g., bed placed in Trendelenburg position, warm compresses applied to IV access site).
- ***No action*** — no actions were ordered for management of the AE.

12.3.7 Outcome

Follow-up of AEs. If possible, AEs will be followed until resolved (synonyms: recovered, recuperated, ended) either with or without sequelae, including for subjects who prematurely discontinue study participation. For AEs that are assessed as not drug-related and are not resolved at the End-of-Study visit, follow-up may be limited with the approval of the Medical Monitor.

Outcome of AEs. The outcome of each event will be described using the following categories:

- ***Resolved (recovered) without sequelae*** — the event resolved and subject returned to baseline;
- ***Resolved (recovered) with sequelae*** — the event resolved but the subject is left with residual problems (e.g., functional deficits, pain);
- ***Resolving (recovering)*** — at the last observation, the event was improving;
- ***Not Resolved (not recovered)*** — at the last observation, the event was unchanged;
- ***Death (Fatal)*** — to be used for the *one* AE which, in the judgment of the Investigator, was the *primary* cause of death;
- ***Unknown*** — there were no observations after the onset (initial observation or report) of the event.

Note: Resolving and Not Resolved may also be used for AEs that were unresolved at the time a subject died, but were *not* assessed as the primary cause of death.

12.3.8 Date and Time of Outcome

For each class of outcome as defined above, Table 12-3 indicates the date and time to be recorded. As discussed in detail for date / time of onset (see Section 12.3.2), determining the date / time an event resolved (ended) should reflect the type of event and the source of the information.

Ford von Reyn 5/31/15 10:25 AM
Deleted: Table 12-3

Table 12-3. Date and Time of Outcome for AE by Outcome Class

Outcome assigned to AE	Date and Time to be Recorded
Resolved (with or without sequelae)	Date and time event observed or reported as resolved
Death	Date and time of death
Resolving or Not Resolved	Date and time of last observation
Unknown	None (see definition above)

12.4 Reporting of Serious Adverse Events

12.4.1 Where to Report SAEs

SAE reporting forms with detailed instructions will be provided during training. Serious adverse events will be entered immediately into the electronic Source Tablet prompting automatic notification of all relevant study personnel, including the Medical Monitor. The CRO will work with the Site to collect any additional data needed to further evaluate the SAE. Reports and supporting materials relating to SAEs should be submitted as scanned documents. The Site will enter the data required into the electronic Source Tablets. Data received from outside sources such as local labs or a Discharge Summary will be stored electronically.

The Investigator will notify the IRB of SAE's based on reporting requirements. Contact information is provided in Section 17.

12.4.2 Procedures for Reporting SAEs to the Sponsor

The ***initial notification*** should be completed in the eSource for each SAE within 24 hours of the time the Investigator (or the Investigator's designee) becomes aware that the event has occurred and will include the following items of information (any items not available should be explicitly noted):

- protocol number, study site, subject number;
- Investigator's name, address, and contact information (phone, fax, email);
- description of the event (i.e., date and time of onset, initial assessment, treatments and course);
- current status of the subject and the event;
- criteria by which the event was assessed as serious;
- date of the first injection of study drug;
- date of the last injection of study drug prior the event;
- assessment of relationship of study drug to the event;
- whether the study drug was discontinued or adjusted as a result of the event.

The ***initial full report***, signed by the Investigator, will be submitted within two days for death and life-threatening events and within four days for all other SAEs; the report will include all of the above information *plus* the following items:

- narrative summary of the event — to include specific information that will assist in understanding the event, e.g., relevant medical history, co-morbid conditions, physical exam, diagnostics, assessment, treatments (including concomitant medications), response to treatment, course, and outcome (if known);
- copy of the completed AE page of the CRF (or completion of online data entry);
- copies of relevant medical reports — including diagnostic procedures (e.g., laboratory, ECG, x-ray), surgical procedures, and consultations.

Thereafter, signed ***supplemental reports*** will be submitted as any additional information (e.g., more definitive outcome regarding events previously reported as ongoing or unknown outcome) becomes available to the Investigator (either directly or as a result of investigation into a query).

12.4.3 Requirements for Expedited and Periodic Reporting of Adverse Events

SUSARs are required to be reported rapidly to the DRC, regulatory authorities and to EC/IRBs (within seven days for fatal or life-threatening SUSARs; within 15 calendar days for all other SUSARs). There are varying requirements for periodic (annual or semi-annual) reporting of all SUSARs and, in some cases, all SAEs. The Sponsor and the Investigator will work together to meet these reporting requirements.

12.4.4 Notification of SAEs to the Investigator by the Sponsor

In accordance with regulatory requirements, the Sponsor will notify the Investigator of the occurrence of SUSARs reported by other Investigators in this or in other studies involving the study drug. The Investigator will promptly inform his/her IEC of such communications from the Sponsor and will document that notification in the Investigator's Regulatory Binder.

12.5 Sponsor Guidance for Grading of Injection Site Reactions

As detailed in Section 5.5, the adverse events associated with intradermal injection of SRL 172 were primarily injection site reactions similar to, but generally milder than, reactions to BCG vaccination, which has been used world-wide for over 70 years. Further, DAR-901 is a heat-inactivated vaccine with no living organisms, so the rare invasive BCG complications of lymphangitic or hematogenous spread are not under consideration, even in HIV-infected persons.

12.5.1 Definitions of the Most Commonly Expected Systemic Vaccine-related Symptoms

- Fever: elevated temperature documented by any route during a visit or by the subject at home.
- Feverish: subjective fever reported by the subject, but not documented.
- Malaise, myalgia, "flu-like" symptoms: will be defined consistent with routine clinical practice.

12.5.2 Definitions of the Most Commonly Expected Injection Site Symptoms

- Tenderness: discomfort elicited when the area is touched either intentionally or accidently.
- Pain: discomfort or unpleasant feeling (e.g., headache, stubbed toe) experienced while at rest or with activity; in addition to location, the patient's description may include intensity as well a distinctive quality (e.g., burning, stabbing). In the SRL 172 trials (see Section 5.5), these events were reported as "sore arm."
- Pruritus (itch): an unpleasant sensation that evokes the desire or reflex to scratch. (In contrast, pain and tenderness evoke a reflex to withdraw.)

12.5.3 Definitions of the Most Commonly Expected Injection Site Findings

- Erythema: reddening of the skin.
- Desquamation: skin coming off in scales, often patchy or circumferential; maximum linear diameter will be recorded only if the area of involvement is a continuous patch.
- Induration: an area of skin that is thicker, firmer than usual. Will be used to include both the related terms papule / nodule (a solid raised lesion with distinct borders, <1 cm diameter) and plaque (papule-like lesion, >1 cm), since diameter will be recorded independently.
- Vesicle / Blister: a sub-epidermal collection of clear fluid
- Pustule: a sub-epidermal collection of white or yellow fluid up to 2.5 cm diameter that based on appearance is presumed to be "pus", i.e., to contain neutrophils. Commonly seen after intradermal injection of BCG or heat-killed mycobacteria, but rarely infected with pyogenic bacteria.
- Erosion: the loss of the surface of the skin; typically results in a shallow moist or crusted lesion. In the studies with SRL 172 (see Section 5.5), this process was reported as "skin breakdown".

- Ulceration: full thickness loss of epidermis, with erosion into dermal or deeper tissue; commonly crusted or with granulation.
- Crust: dried material covering an erosion or ulceration; may be white or colorless if composed of plasma or exudate, or darker if small amounts of blood are present.
- Eschar: hard dry plaque covering an ulcer, implying underlying tissue necrosis
- Abscess: a sub-epidermal collection of white or yellow fluid greater than 2.5 cm diameter that based on appearance is presumed to be "pus", i.e., to contain neutrophils. If closed, the lesion is typically fluctuant, that is yields to palpation consistent with containing fluid. Abscesses may open and drain spontaneously or may be incised and drained by the investigator. Abscesses will be classified as "Sterile" or "Infected (Pyogenic)" (see below).
  - If an abscess is incised by the investigator, a fresh culture should be obtained. Open lesions have a high likelihood of contamination and culture is generally only useful if there are other findings, such as surrounding cellulitis or acute systemic symptoms.
  - The investigator may prescribe topical or systemic antibiotics based on their judgment of the risk of current or potential pyogenic involvement. The expected pathogens would be *Staphylococcus aureus* or β-hemolytic streptococci.
- Sterile Abscess: In a recent prospective, active surveillance of BCG immunization, sterile abscess was reported in 3.6% (18/504) persons over 6 months of age [18]. In the DarDar trial, sterile abscess was observed in 0.3% of HIV-infected adults who received SRL 172 [1]. Neither study reported any pyogenic abscesses. Sterile abscesses are typically not accompanied by surrounding erythema, warmth, or marked tenderness, or by fever or regional lymphadenopathy [19].
- Infected Abscess: An abscess due to pyogenic bacteria, typically *Staphylococcus aureus*. In addition to documented positive cultures, pyogenic abscess is expected to be accompanied by at least one of the following: surrounding erythema, warmth, or marked tenderness. Infected abscess also often present with fever and regional lymphadenopathy.
- Scar: an area of fibrous tissue that replaces normal skin after injury; a natural sequela of wound repair and healing. Often associated with mild discoloration.

12.5.4 Visit Assessment of Injection Site Reactions and Other Post-Dosing Events

At each visit specified in Sections 2.9 and 12.2, the subject will have vital signs obtained, be interviewed by study personnel regarding symptoms and other events, and examined by the Investigator.

Treatment-emergent changes in vital signs will be graded using the criteria shown in Table 20-2, which are taken from FDA Guidance [20].

Subjects will be explicitly asked about systemic and local symptoms (see Sections 12.5.1 and 12.5.2, respectively), any interference with daily activities, and any treatment required. Symptoms will be graded using the functional criteria shown in Table 12-4.

Investigator will examine all of the prior injection sites and then:

- Record the presence or absence of the physical findings defined in Section 12.5.3;
- For any findings present, record the maximum linear diameter in mm;
- Pain, erythema, and induration will be graded as shown in Table 12-5, taken from FDA Guidance [20]; other ISRs characteristics will be graded using the functional criteria shown in Table 12-4.
- Considering *all* the findings present, the investigator grade the *overall* intensity (severity) of the ISR as per Table 12-4.
- This grade should be "static", that is, based upon the impact and management of the ISR at the time, without reference to previous observations for the subject.
- Subjects will be asked for permission to photograph the ISR if it is assessed as Grade 2 or higher, is accompanied by systemic symptoms, or is managed with prescription medication (e.g., systemic antibiotics or prescription analgesics). Photographs are completely optional and may be declined by the subject without impacting any other aspects of the protocol.

Ford von Reyn 5/31/15 10:25 AM
Deleted: Table 20-2

Ford von Reyn 5/31/15 10:25 AM
Deleted: Table 12-4

Ford von Reyn 5/31/15 10:25 AM
Deleted: Table 12-5

Ford von Reyn 5/31/15 10:25 AM
Deleted: Table 12-4

Ford von Reyn 5/31/15 10:25 AM
Deleted: Table 12-4

Table 12-4. Grading of Vaccine-Related Adverse Events

Characteristic	Grade 1	Grade 2	Grade 3
**Impact on ADL**	No limitations in ADL	Some limitations in age-appropriate instrumental ADL	Some limitations in self-care ADL
**Medication intervention**	None or self-medication with OTC meds	Prescription meds offered; provide relief	Requires prescription meds; relief may be partial
**Interventions other than medication**	Minimal, local, or non-invasive	Minimal, local, or non-invasive	Requires hospital facilities for <24 hr

This Table is intended to provide guidance; the investigator should use judgment in assigning an intensity grade to an ISR. In some instances a single characteristic may determine the grade; in other instances, the overall pattern may be considered more appropriate.

Table 12-5. Grading of Common Injection Site Reactions

Local Injection Site Reaction	Grade 1	Grade 2	Grade 3	Grade 4
Pain	Present but does not interfere with activity	Repeated use of non-narcotic pain reliever >24 hours ***or*** interferes with activity	Any use of narcotic pain reliever ***or*** prevents daily activity	Emergency room (ER) visit ***or*** hospitalization
Erythema[a]	2.5 – 5 cm	5.1 – 10 cm	> 10 cm	[c]
Induration/Swelling[b]	2.5 – 5 cm ***and*** does not interfere with activity	5.1 – 10 cm or interferes with activity	> 10 cm or prevents daily activity	[c]

a. Erythema (Redness) should be measured at the maximal diameter and the measurement should be graded and also recorded as a continuous variable.
b. Induration (Swelling) should be measured at the maximal diameter and the measurement will also be recorded as a continuous variable; the event should be graded using the functional scale as well as the actual measurement.
c. Note that Erythema and Induration, in and of themselves, are not "life-threatening (Grade 4)" events; however, they may progress to new events, such as exfoliative dermatitis or necrosis, that should be recorded and graded separately.

12.5.5 Subject Monitoring of Injection Site Reactions

As specified in Sections 2.9 and 12.2, between scheduled visits, subjects will be contacted by telephone twice weekly to monitor systemic and local reactions to vaccine administration. The calls will be conducted using a detailed script and data collection form to be sure that both expected and uncommon events are elicited and recorded. The process will be facilitated by providing subjects with the following:

- an electronic oral thermometer to monitor temperature daily
- paper rulers with which to measure any ISRs
- a diary on which to record temperature, systemic symptoms, and local ISRs.

Figure 20-1 shows the Subject Diary Instructions page; Figure 20-2 shows a sample record page.

Ford von Reyn 5/31/15 10:25 AM
Deleted: Figure 20-1
Ford von Reyn 5/31/15 10:25 AM
Deleted: Figure 20-2

During the telephone follow-up calls, study staff will collect the information recorded on the subject's diary / memory aid for each day since the last visit / call completed with the subject. Subjects will then be asked the following questions:

- Have you had any other reactions or events other than those indicated on the diary?
- How they are feeling today (the day of the call)?
  - Collect details of any spontaneously reported symptoms.
- Today, are you having any …. (ask specifically the symptoms noted on the dairy, unless already reported by the subject)?
- Have you taken any over-the-counter medication since the last visit/call?

- If so, for what symptoms? Which medicine? What dose? What frequency?
- Did the medication relieve your symptoms?

- Have you taken any prescription medication since the last visit/call?
  - If so, what symptoms? Which medicine? What dose? What frequency?
  - Did the medication relieve your symptoms?
- Have any of the symptoms interfered with your usual daily activities, such as exercise, playing the piano, holding a book to read?
  - Have you been unable to do any of your usual daily activities?
  - Have you been unable to attend work or classes?
- Have any of these symptoms interfered with activities related to taking care of yourself, such as dressing, cooking, brushing your teeth, going to the bathroom.
  - Have you required assistance for any of these activities?

12.6 Grading of Specific Laboratory Safety Tests for Reporting and Analysis

For specific laboratory safety tests shown in Table 20-1 all abnormal results will be graded using the criteria shown, which are taken from the "FDA Guidance for Industry. Toxicity Grading Scale for Healthy Adult and Adolescent Volunteers Enrolled in Preventive Vaccine Clinical Trials. September 2007."[20].

Ford von Reyn 5/31/15 10:25 AM
Deleted: Table 20-1

The grading will be used both in reporting AEs and in the data presentation and analysis of laboratory results. Specifically, the data listings will indicate the appropriate Grade and treatment-emergent changes in these laboratory tests will be summarized as "shift tables" using these grades (see Section 14.4). This process will assure that the final study report contains complete and consistent analyses of these laboratory safety results.

Treatment-emergent abnormal laboratory results for analytes not shown in Table 20-1 will be reported as AEs using the procedures and criteria detailed in Section 12.1.5.

Ford von Reyn 5/31/15 10:25 AM
Deleted: Table 20-1

13. DATA QUALITY ASSURANCE

13.1 Compliance

The Sponsor and the Investigator will conduct the study in accordance with:

- The protocol — as approved by applicable regulatory authorities;
- Ethical standards and procedures — as detailed in Section 15;
- "Good Clinical Practices" and "Good Manufacturing Practices" — as detailed in documents issued by the International Committee on Harmonization (ICH);
- Applicable national regulations — e.g., in the US, 21 CFR.

13.2 Training and Qualifications of Site Personnel

All site personnel involved in the study will be trained regarding the protocol and the study drug. This includes, but is not limited to, pharmacy, nursing and medical personnel involved in handling and administering the study drug, monitoring the subjects and collecting clinical data.

The Sponsor (or designee) will provide formal training sessions either off-site (e.g., Investigators Meeting) or on-site (e.g., site initiation visit). Topics covered will include, but not be limited to, background of the investigational drug, the protocol, study events, study procedures, data collection and recording, expedited and routine reporting of adverse events, and regulatory requirements. It is the responsibility of the Investigator to notify the Sponsor of any new study personnel and to work with the Sponsor to ensure that they receive adequate training.

13.3 General Procedures for Completing Data Collection

All data will be collected on electronic Tablets specifically designed for to collect both the Source data and the Clinical Database Data. This system enables including instructions for visits, source data that helps the Site to complete the protocol and data for the Clinical Database.

The system is 21 CFR Part 11 compliant and includes a full audit trail of all data entered and reviewed. There is Role-Based Access so that data containing personal health information will only be visible by the Site, the Site Monitors and auditors. The informed consent will reflect this approach.

All users of the system will receive training to assure they are able to complete the data entry on the tablet. Access to the system for the actual study will not be provided until training is complete.

13.4 Case Report Forms

The Sponsor will provide structured forms for reporting study data to a central facility holding the trial database. These case report forms (CRFs) will be electronic (i.e., "electronic data capture" — EDC). The Investigator (or qualified sub-Investigator approved by the Sponsor) will review all CRFs and indicate their concurrence by either a manual or electronic signature, as appropriate. The Sponsor will provide detailed procedures for the system used in the study.

13.5 Source Documents

Source documents are the originals of any documents used by the Investigator, hospital, or institution that verify the existence of the subject and substantiate the integrity of the data collected during the trial. Unless otherwise specified by the Sponsor, source documents will be available to support all the data recorded on the CRF and SAE forms. Source documents forms created exclusively for the purpose of this study (e.g., screening logs, study procedures worksheets) must be reviewed by the Sponsor prior to use.

Source documents may include, but are not limited to, the following:

- the informed consent form, signed and dated by the subject;
- information obtained from the subject's personal physicians or other third parties regarding the subject's medical history or prior physical condition;

- screening logs;
- recorded data and reports from automated instruments (e.g., ECGs, cardiac monitors, vital signs), including annotations of abnormal findings;
- laboratory reports (e.g., hematology, clinical chemistry, urinalysis, urine microscopy), including annotation of abnormal results;
- concomitant medication prescription and administration records;
- medical records relating to scheduled and unscheduled study visits, including, but not limited to, results of examinations, observations relating to AEs, and concomitant medications.

If the Source is not the electronic Source designed for the study, paper source will be converted to electronic format and maintained as part of the Study Record. Data from labs will be automatically uploaded into the electronic Source

In addition to the practices noted for CRFs (see Section 13.3), source documents must also meet the following requirements:

- Be prepared at the time of the events or activities described (i.e., contemporaneously);
- Indicate both the date and time recorded;
- Identify the source of all recorded information (e.g., the subject, direct observations of the recorder, lab reports, external / historical sources).

13.6 Protocol Deviations

Conduct of the study will be monitored to ensure that protocol deviations are minimized. A protocol deviation is defined as an event in which the Investigator or site personnel did not conduct the study according to the Protocol, including compliance requirements and agreements.

For protocol deviations relating to individual subjects, the event and relevant circumstances will be recorded on source documents and on the appropriate CRF; reported to the Sponsor in a timely manner; and presented in the Clinical Study Report.

Deviations that are not subject-specific (e.g., unauthorized use of an investigational agent outside the protocol, either human administration or laboratory use; non-compliant actions involving another study by site personnel also involved in both this protocol) will be reported to the Sponsor in writing and copies placed in the Trial Master File.

Deviations that can be anticipated should, if possible, be discussed with the Sponsor before being implemented.

13.7 External Review of the Study Conduct at Participating Sites

All study-related materials at the site are subject to external review to ensure the safety of the subjects, the integrity of the study data, and compliance with all applicable regulatory and oversight requirements.

There are several different classes of review:

- Monitoring — review by the Sponsor or authorized representatives, typically from the CRO coordinating the clinical conduct of the trial;
- Audits — independent review by the quality assurance department of the Sponsor or authorized representatives, potentially from an organization not involved in the clinical conduct of the study;
- Regulatory review — performed by representatives of regulatory authorities with responsibility for oversight of the trial or approval of the investigational agent. These authorities may be from the country where the site is located or from another country.

Activities during these on-site reviews may include, but are not limited to:

- Inspection of the facilities (e.g., clinical and administrative areas, pharmacy, laboratory);
- Review of the site trial master file, including documentation related to the protocol, the Investigator, and other study site personnel; correspondence to and from the IRB, the Sponsor, and their representatives;

- Review of standard operating procedures and current practices relating to clinical and pharmacy activities, data handling, the IEC oversight and the informed consent process;
- Review of source documents supporting all data collected during the study (e.g., inclusion/exclusion criteria, informed consent forms, HIPAA authorizations, adverse events records, expedited event reporting, efficacy endpoints);
- Resolution of any discrepancies noted.

Monitoring and auditing visits on behalf of the Sponsor will be scheduled with the Investigator in advance and will be conducted at a reasonable time. To facilitate these visits, the Investigator will assure that the following are available:

- appropriate space, facilities and access to all source documents (including access to computerized records either electronically or as complete print outs);
- consent forms, CRFs, SAE forms, and medical records for all screened and enrolled subjects;
- timely access to site personnel, including the Investigator, sub-Investigator(s), and other study personnel on the day of the visit to resolve any questions that arise.

Regulatory authorities may visit and review the site and/or Investigator during or after the study and may or may not notify the Investigator or the Sponsor in advance. The Investigator will fully cooperate with regulatory audits conducted at a reasonable time in a reasonable manner. The Investigator will notify the Sponsor immediately of any contact by or communication from regulatory authorities regarding the study.

13.8 Resolution of Deficiencies

The Investigator agrees to take promptly any reasonable steps requested by the Sponsor to resolve any deficiencies identified as a result of monitoring, audits, inspections, protocol deviations or review of any other study documentation. Failure to take adequate remedial action can result in suspension or termination of the study at the site.

13.9 Study Closeout

The study will be considered complete when all of the following have occurred:

- All treated subjects have completed all scheduled visits plus any unscheduled follow-up required by AEs;
- All CRFs have been completed, submitted and all queries resolved;
- The trial database has been locked.

The Sponsor or designee will then conduct a study closeout visit, which may include, but is not be limited to, any of the following:

- Review the site Trial Master File to assure all required regulatory documents are current and complete;
- Resolve any open issues from prior monitoring, audit or inspection visits;
- Review the site’s provisions for meeting the requirements for retention study records;
- Discuss possible future site audits;
- Review the Sponsor’s publication policy;
- Confirm compliance with requirements for notifying the IRB of study events, including closure;
- Collect any unused study materials for either return to the Sponsor or disposal in a manner approved by the Sponsor.

13.10 Record Retention

All study-related materials at the site (e.g., source documents, CRFs, Trial Master File) will be retained according to ICH guidelines and applicable regulations.

The study drug is being developed under a U.S. Investigational New Drug (IND) application; regulations require all study-related materials be retained for ***at least 2 years after*** one of the following events:

- approval of a New Drug Application based on this study;
- notification by the Sponsor that no further application will be filed.

The Investigator will use the following procedures regarding retained records:

- Contact the Sponsor ***before*** destructing any records pertaining to the study;
- Provide the Sponsor an opportunity to collect the records;
- Obtain written permission from the Sponsor to destroy the records;
- Notify the Sponsor if the Investigator plans to leave the institution so that arrangements can be made for the transfer of records;

Clinical and laboratory samples that are unstable may be disposed with the written approval of the Sponsor.

13.11 Data Management

A detailed Data Management Plan will be prepared separately and approved by the Sponsor.

14. STATISTICAL METHODS

The sections below indicate the overall structure and approach to the analysis of this study. A detailed Statistical Analysis Plan (SAP) incorporating these sections below will be prepared separately and approved by the Sponsor. The SAP will define populations for analysis, outline all data handling conventions, including software, and specify additional statistical methods to be used for analysis of safety, efficacy and pharmacokinetics.

14.1 Power and Sample Size

Given the exploratory nature of this study, the sample size is based not on power calculations but rather on prior Phase 1 experience indicating that cohorts of this size are the minimum required to provide sufficient data for safety, tolerability, pharmacokinetics and pharmacodynamics.

14.2 Analysis Populations

- *Safety population* – all subjects who received at least one dose of the study medication.
- *Immunogenicity populations* – all subjects with evaluable immunogenicity data, respectively, based on protocol compliance, adequate numbers of samples and successful sample assays.

Within each population, analyses will be performed comparing subjects by dose level.

14.3 Statistical Methods

Study results will be analyzed using descriptive statistics. Adverse events will be listed per dose level and analyzed by intensity and relationship to investigational product. All other variables will be analyzed descriptively per dose level. No formal statistical comparisons are planned.

14.4 Safety Assessments

The primary endpoint will be a comprehensive evaluation of AEs and/or toxicity based on:

- subject reports;
- investigator observations of the subject (history and physical examination);
- vital signs;
- safety laboratory tests, including clinical chemistry, hematology, coagulation, complement and urine analysis tests;
- need for concomitant medications.

14.5 Immunogenicity Analyses

All immunogenicity analyses will be based on subjects who received at least one dose of study vaccine. Immunogenicity will be summarized for all time points as collected and as available. No imputation for missing data will be performed. Data will be transformed as appropriate prior to analysis. Additional analyses may be performed based on subjects who complete all scheduled vaccinations, on HIV and LTBI status. Dose-finding requirements for immunogenicity will be detailed in the statistical analysis plan.

The immunogenicity of DAR-901 will be assessed primarily by determining change IFN-γ response to DAR-901 as measured by ELISA, and IFN-γ ELISpot before and after immunization,. Additional exploratory assays will be conducted as detailed below. Due to the exploratory nature of immunogenicity endpoints, the primary evaluation will be based on descriptive summaries and no formal hypothesis testing will be performed.

14.5.1 IFN-γ assay by ELISA.

Briefly, freshly isolated and ficolled PBMC are incubated in triplicate with study antigens for five days after which centrifuged cell supernatants are used for later IFN-γ level measurement using a standard IFN-γ ELISA (R&D Systems, Minneapolis, MN). Study antigens are medium alone (negative control), 1 mcg/ml *M. tuberculosis* ESAT-6, 0.5 mcg/ml *M. tuberculosis* Ag85, or 0.5 mcg/ml *M. tuberculosis* whole cell lysate (WCL).

14.5.2 ELISpot Assay

ELISpot procedures will be performed according to Aeras SOP IMM-172 Human Interferon-γ ELISpot. Following overnight rest, the coated ELISpot plates will be blocked with R10 medium. After ICS stimulation is set and the ELISpot plates have been blocked, ELISpot antigen stimulation for assessment of IFN-γ production will be performed as follows: negative control, SEB as a positive control, and experimental stimulation conditions (mycobacterial lysate, peptides, etc). Stimulation conditions are pre-diluted in R10 medium. Note that the ELISpot will be ideally stimulated with each condition in triplicate. A total of 200,000 cells per well are used for stimulation with a final volume of 200 microliters in each well. Once plated, cells will be incubated with the above solution 18-24 hours at 37°C and 5% CO_2. Following stimulation, sample wells will receive a mouse-derived anti-IFN-γ detection antibody (MabTech, Sweden) for 2-3 hours incubation at room temperature, then an anti-mouse secondary antibody conjugated with alkaline phosphatase (Fisher, USA) for another 2-3 hour incubation at room temperature. The ELISpot plates will be developed with filtered NBT/BCIP (Fisher, USA), dried, and then analyzed using a CTL C6 Core Analyzer (CTL, USA).

14.5.3 Intracellular Cytokine Staining (ICS)

Stimulation and intracellular cytokine staining will be performed according to Aeras SOP IMM-161 Stimulation and Staining Procedures for Expanded Human ICS. Antigen stimulation for assessment of cytokine production by ICS will be performed using dimethyl sulfoxide (DMSO, Sigma) as a negative control, staphylococcal enterotoxin B (SEB; 0.5 µg/mL) as the positive control, and experimental stimulation conditions (mycobacterial lysate, peptides, etc). Stimulants or controls are pre-diluted in R10 medium containing CD107a-Alexa488 and costimulatory antibodies CD28 and CD49d. Following 2 hours of incubation, GolgiStop and GolgiPlug (BD Biosciences, USA) are added to the stimulating cells and incubated for an additional 6 hours. GlogiStop and GolgiPlug are both protein transport inhibitors that help to capture cytokines intracellularly. After incubation, the cells will be washed and stained with viability dye (Life Technologies, USA) before staining with fluorochrome-conjugated antibodies to surface markers CCR7, CD4, CD8, CD14, CD19 and CD45RO, then permeabilized and stained for CD3, IFN-γ, IL-2, TNF, IL-22, IL-17A, and CD154. All samples will be stained with anti-CD107a-Alexa Fluor 488, anti-CCR7-Brilliant Violet 605, anti-CD3-ECD, anti-CD4-APC-eFluor 780, anti-CD8-Alexa Fluor 700, anti-CD14-V500, anti-CD19-V500, anti-CD45RO-Brilliant Violet 785, anti-IFN-V450, anti-TNF-PE-Cy7, anti-IL-2-PE, anti-IL-22-APC antibodies. Following incubation, cells will be washed, fixed, and analyzed by flow cytometry. For ICS data acquisition, the BD LSR II flow cytometer (BD Biosciences, US) will be set to collect up to 150,000 viable $CD3^+$ target cell events from each sample. All sample analysis will be performed with FlowJo software (TreeStar Inc., USA)

14.5.4 Mycobacterial Growth Inhibition Assay

The mycobacterial growth inhibition assay (MGIA) is employed to assess efficacy of the vaccine candidate using BCG as a correlate for *Mtb*. This assay makes use of the BD Bactec MGIT 960 instrument and its companion MGIT tubes. These tubes contain 7H9 mycobacterial growth medium, a growth supplement containing 5 antibiotics (PANTA) to prevent growth of non-mycobacterial microorganisms, and a colorimetric indicator of oxygen quenching. The instrument acts as a 37°C incubator that records incubation start time and scans each tube hourly for changes in oxygen quenching (a correlate of BCG growth within the tube). Using proprietary "growth units" developed by BD, the instrument determines the time to positivity (TTP) for each tube once the indicator meets or exceeds 75 growth units.

For the inhibition assay, cryopreserved PBMCs from trial volunteers are thawed, washed with fresh culture medium to remove residual cryoprotectant, and then rested in fresh cell culture medium at 37°C for 2-18 hours. Following rest, specimens are counted and concentration adjusted to 3.33×10^6 cells/mL. From the adjusted concentration, approximately 1×10^6 cells are transferred to a sterile, gasketed polypropylene tube. The trial specimens are subsequently infected with a known quantity of BCG (with a target TTP of 6.5 to 13 days) from a frozen BCG stock. Tubes are prepared in duplicate in order to

confirm results. The infected PBMCs are incubated for 4 days at 37°C in a tube rotisserie. At the time of BCG inoculation, two control tubes are also prepared by inoculating the same quantity of BCG from the same stock into two BD MGIT tubes, and placed in the BD BacTec MGIT 960 instrument.

Following the four day incubation, the trial specimen tubes are harvested and the contents are used to inoculate fresh BD MGIT tubes, which are then inserted into the Bactec MGIT instrument. The instrument will determine and record the time to positivity (TTP) for each specimen, including the inoculum control tubes. Once recorded, the TTP for each specimen in the experiment, as well as the inoculum control tubes, are entered into a macro created by the Jenner Institute. This macro compares the TTP of each specimen to the control tubes to determine a Δ log increase or decrease in BCG growth. The Δ log values for the specimens can then be compared to show relative efficacy.

14.5.5 LAM assay.

Antibody to mycobacterial lipoarabinomannan (LAM) will be measured in subject serum using a standard ELISA. Serum from a subject with known LAM responses will be used as positive control while a pool of serum samples from TB-naive individuals will be used to generate range of negative controls.

14.6 Identification of Study Event Days and Times

Study events will be recorded using the calendar date and (where applicable) the time to the nearest minute.

For purposes of post-study analysis (e.g., tables and listings), study days will be designated as follows:

- Day 1 is defined as the calendar day of the first injection of study drug.
- The days prior to Day 1 are designated Day –1, Day –2, etc; there is no Day 0.
- The days following the day of the first injection of study drug are designated Day 2, Day 3, etc.
- The day of the last injection of study drug is indicated by adding the suffix "L", e.g., if the last injection is administered on Day 22, it will be displayed as "Day 22L".
- The days following the last injection of study drug are designated Day 1P, Day 2P, etc.

The times of events related to dosing of study drug will be designated as minutes or hours before or after the time of dosing (i.e., the subcutaneous injection of study drug), which is designated as t = 0 (zero). Thus, 15 minutes prior to dosing is t = −15 min; 2 hour after dosing is designated t = 2 h.

14.7 Handling Missing Data

In general, missing data will not be imputed. Further details for handling of missing, duplicated or unscheduled data will be given in the Statistical Analysis Plan.

14.8 Changes in the Planned Analyses

If changes are made to the Statistical Analysis Plan, then these will be listed in the Clinical Study Report, along with an explanation as to why they occurred.

15. ETHICAL CONSIDERATIONS

15.1 Independent Ethics Committee (IEC)

Prior to initiating the study, the Investigator will submit the following to an IEC[23] for approval:

- Study protocol;
- Investigator's Brochure;
- Informed Consent Form and any other written documents to be given to the subject;
- details of any compensation to subjects;
- any other requested document(s).

The study will not commence until the IEC has issued a letter of approval signed and dated by the IEC chair or authorized person which includes the following items:

- protocol number, full title, version number and date;
- version date of the Informed Consent Form;
- version date of the applicable Investigator's Brochure;
- date the protocol and consent form were reviewed and approved by the IEC.

The Sponsor or designee will be provided copies of all correspondence between the Investigator and the IEC. In addition, prior to study initiation, the Sponsor will be provided *one* of the following to verify that the IEC was appropriately qualified to approve the protocol:

- Documentation that on the date of the approval, the IEC met all currently applicable regulatory requirements for policies and procedures (e.g., membership, quorum, and approval procedures);
- A memo listing the voting members of the IEC who were present at the meeting the protocol was approved, including their titles, occupations, and institutional affiliations.

The Investigator will submit to the IEC, at least annually, a report of the study's progress.

15.2 Ethical Conduct of the Study

The study will be conducted in accordance with:

- the current version of "Ethical Principles For Medical Research Involving Human Subjects" as adopted by the World Medical Association (WMA);[24]
- local laws and regulations for the use of investigational therapeutic agents.

15.3 Subject Information and Consent

Informed Consent Forms submitted to the IEC must be (a) based on a master document provided by the Sponsor and (b) reviewed and approved by the Sponsor prior to submission to the IEC. The Sponsor must also review and approve any changes requested by the IEC prior to the ICF being used.

Informed consent will be obtained prior to conducting any study procedures that are not part of the subject's routine medical care. During the consent process, each subject will:

- Be advised of the nature and risk of the study by the Investigator or designated study personnel;
- Be given sufficient opportunity to read the ICF, to ask any questions, and to consider whether to participate;
- Provide informed consent voluntarily.

[23] ICH E6, which specifies GCP, requires "an independent body (a review board or a committee, institutional, regional, national or supernational) …. whose responsibility it is to ensure the protection of the rights, safety, and well-being of human subjects involved in a trial…" In this protocol, the body performing this function will be referred to as the IEC (Independent Ethics Committee); in practice, many alternative designations are used, e.g., Institutional Review Board (IRB).

[24] This document, commonly referred to as the "Declaration of Helsinki", was issued in 1964 and has been amended or clarified at subsequent WMA Assemblies. Only the current document is considered official by WMA. The most recent version was approved in October 2008 (59th WMA General Assembly, Seoul, Korea).

The ICF will be signed and dated by the subject and by the person who provided the information. A copy of the signed ICF will be provided to the subject; the original will be retained by the Investigator as a source document. The informed consent process will be noted in the source documents.

The subject will be informed in a timely manner if new information becomes available that may be relevant to the subject's willingness to continue participation in the trial. Communication of this information to the subject will be noted in the source documents.

15.3.1 Obtaining Informed Consent from Subjects Who Are Not Literate

Subjects not literate in English will not be eligible for enrollment.

15.3.2 Special Informed Consent Situations Not Applicable to This Protocol

Subjects may not be enrolled if they meet ***any*** of the following conditions which require specific provisions and approvals not provided for in this protocol:

- Are not able to provide informed consent (e.g., are acutely or permanently impaired);
- Are at increased risk of coercion (e.g., prisoners, institutionalized persons);
- Are less than 18 years of age.

15.4 Protection of Subject Information

The identity and collected data of each subject ("protected health information") will be kept confidential and will be protected in accordance with applicable local regulations.

Methods to be used to protect the data will include the following:

- Each subject will be assigned a unique subject number, which will be used on the CRF in place of the subject's name.
- Computer systems for collecting and analyzing the data will have restricted access.
- In publications, aggregate data will be used wherever possible; any individual data will be redacted of unique identifying characteristics.

The informed consent process will comply with local requirements relating to (a) disclosure of the data to be collected and (b) authorization for its use. When permitted, these issues will be included in the ICF. In the event a separate form is required, the following will apply:

- The Sponsor must review and approve the separate form.
- The form will be signed and dated by, and copies provided to, the required parties.
- A completed copy of the form will be placed in the trial files with the completed ICF.

16. STUDY ADMINISTRATION

16.1 Registration of Study

The Sponsor abides by applicable US regulatory requirements and the guidelines of the International Committee of Medical Journal Editors (ICMJE) regarding registration of controlled clinical trials ("clinically directive trials").

16.2 Changes in the Conduct of the Study

After the Protocol has been approved by the governing IEC and regulatory authority, substantial changes in the conduct of the study will only be made as formal protocol revisions, which must be reviewed and approved by the Sponsor and the Investigator prior to submission to the applicable IEC and regulatory body. Changes will only be implemented after the revised protocol is approved as required.

Changes to contract information or designated study personnel (Section 17) may be handled administratively.

16.3 Confidentiality

This protocol, the applicable Investigator's Brochure, the results of the study and other related information provided by the Sponsor represent confidential and proprietary material of the Sponsor. They will be available only to the Investigator, personnel directly involved in the study, and authorized members and staff of the applicable IEC. These parties agree not to disclose these materials to others.

16.4 Financial Disclosure

In compliance with U.S. 21 CFR 54.4, any listed or identified Investigator or sub-investigator (including the spouse and any dependent children of said individuals) directly involved in the treatment or evaluation of research patients will disclose the following information for the time period during which the Investigator is participating in the study and for 1 year following completion of the study:

- Any financial arrangement between Dartmouth College and the Investigator in which the value of the compensation to the Investigator for conducting the study could be impacted by the outcome of the study.
- Payments (exclusive of the costs of conducting this or other clinical studies) by Dartmouth College totaling >$10,000, including, but not limited to, grants to fund ongoing research, compensation in the form of equipment, retainer for ongoing consultation, or honoraria.
- Any proprietary interest held by the Investigator in the product being evaluated.

16.5 Communication (Publication) Policies

Dartmouth College recognizes the importance of communicating the results of scientific studies, including clinical trials, and, therefore, encourages their publication in reputable scientific journals and presentation at seminars or conferences. Dartmouth also has legitimate responsibilities, including, but not limited to, protecting confidential information about its proprietary products and obtaining patent protection for its intellectual property.

Therefore, the following procedures apply to any communication (including written, oral, or electronic; manuscript, abstract, other publication, or presentation) of results or information arising from this study (including any ancillary studies involving trial subjects) to any third parties:

- The proposed communication will be prepared in collaboration with the Sponsor.
- The final proposed version must be submitted to Dartmouth for review and comment at least 30 days prior to presentation, submission for publication or other dissemination.
- In the event Dartmouth reasonably determines that a proposed communication contains confidential or patentable material, they may require *either* of the following:
  - The material be removed from the communication;

- The communication be delayed for up to 60 additional days to permit filing the appropriate intellectual property protection.

These procedures apply regardless of whether the study is completed as planned or is terminated prematurely for any reason.

The first publication from this study is expected to be a summary of all protocol results, jointly produced by the Sponsor and the participating Investigators.

16.5.1 Authorship and Acknowledgement

All publications will give Dartmouth College and/or their personnel appropriate credit (i.e., authorship or acknowledgement) for any direct contribution made by them.

Authorship will be decided jointly by the Investigators and the Sponsor. Manuscripts will conform to the Uniform Requirements for Manuscripts Submitted to Biomedical Journals, including, but not limited to, the standards for authorship contained therein.

17. CONTACT INFORMATION

Contacts for Expedited Reporting (see Section 12.4):

Contacts

Brenda Haynes, RN
Clinical Trial Manager
Phone: +1 603 650 8840
e-mail: brenda.c.haynes@hitchcock.org

Peter Wright MD
Medical Monitor
Phone: +1 603 650 8840
email: peter.f.wright@dartmouth.edu

Principal Investigator

C. Fordham von Reyn MD
Infectious Disease and International Health
Dartmouth-Hitchcock Medical Center
One Medical Center Drive
Lebanon NH 03756
Tel 603 650 7167
fvr@dartmouth.edu

Dose Review Committee

John F. Modlin MD
Deputy Director for Research
Polio
Gates Foundation
Phone: +1 206 726 7196
john.modlin@gatesfoundation.org

Daniel Hoft MD PhD
Director, Division of Infectious Disease
St. Louis University
Phone: +1 314 977 5500
hoftdf@slu.edu

Robert D. Arbeit MD
Infectious Disease
Tufts University Schoool of Medicine
Phone: +1 671-710-5466
robert.arbeit@gmail.com

18. REFERENCES

1 von Reyn CF, Mtei L, Arbeit RD, Waddell R, Cole B, Mackenzie T, et al. Prevention of tuberculosis in Bacille Calmette-Guerin-primed, HIV-infected adults boosted with an inactivated whole-cell mycobacterial vaccine. AIDS. 2010 Mar 13;24(5):675-85.

2 Stanford JL. Improving on BCG. APMIS 1991:99:103-113.

3 von Reyn CF, Arbeit RD, Yeaman G, Waddell RD, Marsh BJ, Morin P, et al. Immunization of healthy adult subjects in the United States with inactivated Mycobacterium vaccae administered in a three-dose series. Clin Infect Dis. 1997;24:843-8.

4 von Reyn CF, Marsh BJ, Waddell R, Lein AD, Tvaroha S, Morin P, et al. Cellular immune responses to mycobacteria in healthy and human immunodeficiency virus positive subjects in the United States after a five dose schedule of Mycobacterium vaccae vaccine. Clin Infect Dis. 1998;27:1517-20.

5 Marsh BJ, von Reyn CF, Arbeit RD, Morin P. Immunization of HIV-infected adults with a three-dose series of inactivated Mycobacterium vaccae. Am J Med Sci. 1997;313:377-83.

6 Johnson D, Waddell RD, Pelton SI, Jaeger AS, Modlin JF, Yogev R, et al. Randomized trial of intradermal Mycobacterium vaccae or intradermal hepatitis B immunisation in children with HIV infection. Vaccine. 1999;17:2583-7.

7 Waddell RD, Chintu C, Lein D, Zumla A, Karagas M, Baboo KS, et al. Safety and immunogenicity of a 5 dose series of inactivated Mycobacterium vaccae vaccination for the prevention of HIV-associated tuberculosis. Clin Infect Dis. 2000;30 (Suppl 3):S309-15.

8 Vuola J, Ristola M, Cole B, Järviluoma A, Tvaroha SM, Rönkkö T, et al. Immunogenicity of an inactivated mycobacterial vaccine for the prevention of HIV-associated tuberculosis: a randomized, controlled trial. AIDS. 2003;17:2351-5.

9 Kagina BM, Abel B, Scriba TJ, Hughes EJ, Keyser A, Soares A, et al. Specific T cell frequency and cytokine expression profile do not correlate with protection against tuberculosis after bacillus Calmette-Guerin vaccination of newborns. Am J Respir Crit Care Med 2010,182:1073-1079.

10 Lahey T, Arbeit RD, Bakari M, Horsburgh CR, Matee M, Waddell R, et al. Immunogenicity of a protective whole cell mycobacterial vaccine in HIV-infected adults: a phase III study in Tanzania. Vaccine 2010,28:7652-7658.

11 Lahey T, Sheth S, Matee M, Arbeit R, Horsburgh CR, Mtei L, et al. Interferon gamma responses to mycobacterial antigens protect against subsequent HIV-associated tuberculosis. The Journal of infectious diseases 2010,202:1265-1272.

12 Lalor MK, Floyd S, Gorak-Stolinska P, Ben-Smith A, Weir RE, Smith SG, et al. BCG vaccination induces different cytokine profiles following infant BCG vaccination in the UK and Malawi. J Infect Dis 2011,204:1075-1085.

13 Minassian AM, Rowland R, Beveridge NE, Poulton ID, Satti I, Harris S, et al. A Phase I study evaluating the safety and immunogenicity of MVA85A, a candidate TB vaccine, in HIV-infected adults. BMJ Open 2011,1:e000223.

14 Flesch I, Kaufmann SH. Mycobacterial growth inhibition by interferon-gamma-activated bone marrow macrophages and differential susceptibility among strains of Mycobacterium tuberculosis. J Immunol 1987,138:4408-4413.

15 Kolibab K, Parra M, Yang AL, Perera LP, Derrick SC, Morris SL. A practical in vitro growth inhibition assay for the evaluation of TB vaccines. Vaccine 2009,28:317-322.

16 Hoft DF, Worku S, Kampmann B, Whalen CC, Ellner JJ, Hirsch CS, et al. Investigation of the relationships between immune-mediated inhibition of mycobacterial growth and other potential surrogate markers of protective Mycobacterium tuberculosis immunity. J Infect Dis 2002,186:1448-1457.

17 Common Terminology Criteria for Adverse Events (CTCAE). Version 4.02. U.S. Department of Health and Human Services, National Institutes of Health, National Cancer Institute. NIH Publication No. 03-5410, Revised September 2009.

18 Turnbull FM, McIntyre PV, Achat HM, et al. National Study of Adverse Reactions after Vaccination with Bacille Calmette-Guerin. Clin Infect Dis 2002;34:447–53.

19 “Definition and Application of Terms for Vaccine Pharmacovigilance”. Report of the CIOMS/WHO Working Group. Geneva, 2012.

20 FDA Guidance for Industry. Toxicity Grading Scale for Healthy Adult and Adolescent Volunteers Enrolled in Preventive Vaccine Clinical Trials. September 2007.

19. INVESTIGATOR AGREEMENT

I have read the foregoing protocol (version 4.1, 30 March 2015) and agree to the following:

- The protocol contains all necessary details for carrying out this study.
- I will conduct the study as detailed in the protocol and will abide by all its provisions.
- I will conduct the study in compliance with ICH Guidelines for Good Clinical Practice, the requirements of the IEC and all applicable government regulations.
- I will train and supervise all individuals delegated to assist me in conducting this study, including providing copies of the protocol and all pertinent information and discussing the material with them to ensure they are fully informed regarding the investigational drug, the protocol and their responsibilities and obligations.
- I will use only the current informed consent form approved by the Sponsor (or their designee) and by the IRB/IEC responsible for this study.
- I will fulfill all requirements for submitting pertinent information to the IEC and to the Sponsor, including reportable serious adverse events.
- I will complete all case report forms, including resolution of queries, in a timely manner.
- I will provide the Sponsor (or their designee) with access to any source documents from which case report form information may have been derived.
- I will provide the Sponsor with complete, signed statements of financial disclosure as required.
- I understand that the information in this protocol and the referenced Investigator's Brochure is confidential and that its disclosure to any third parties (other than those approving or conducting the study) is prohibited. I will take the necessary precautions to protect this information from loss, inadvertent disclosure or access by third parties.

December 30, 2013

Signature of Principal Investigator *Date*

**Principal Investigator (*print* name)**	C. Fordham von Reyn MD
**title**	Professor of Medicine
**address**	Dartmouth Hitchcock Medical Center<br>One Medical Center Drive<br>Lebanon, NH 03756
**Principal Site of Investigation (name of facility)**	Dartmouth Hitchcock Medical Center
**facility address *(if different from above)***	
